# Effects of Mind–Body Interventions on Immune and Neuroendocrine Functions: A Systematic Review and Meta-Analysis of Randomized Controlled Trials

**DOI:** 10.3390/healthcare13080952

**Published:** 2025-04-21

**Authors:** Shih-Ching Lee, Ping-Han Tsai, Kuang-Hui Yu, Tien-Ming Chan

**Affiliations:** 1 Division of Rheumatology, Allergy and Immunology, Department of Internal Medicine, Chang Gung Memorial Hospital, Taoyuan 333, Taiwan; 2 Graduate Institute of Biomedical Electronics and Bioinformatics, National Taiwan University, Taipei 106, Taiwan; 3 Division of Rheumatology, Allergy and Immunology, New Taipei Municipal Tucheng Hospital, New Taipei City 236, Taiwan; 4 Medical College, Chang Gung University, Taoyuan 333, Taiwan

**Keywords:** mindfulness, immune, neuroendocrine, qigong, yoga, meditation

## Abstract

**Objective**: Chronic stress affects the immune system via the hypothalamic–pituitary–adrenal (HPA) axis and autonomic system. Chronic inflammation is a risk factor for cardiovascular diseases, cancer onset and progression, susceptibility to infection, and cognitive impairment. Mind–body interventions (MBIs) could affect the immune and neuroendocrine systems, and we aimed to assess the correlations among these systems through a meta-analysis. **Methods**: RCTs were identified by searching three databases: PubMed, Embase, and Scopus. Of the 1697 studies identified, 89 were included in this study. Risk of bias was examined using the Cochrane risk-of-bias assessment tool. Data were pooled using a random-effects model, and SMDs were calculated. I^2^ statistics and Egger’s test were used to assess the significance of the asymmetry. Influence diagnostics were used to assess whether pooled effects were disproportionately dependent on any single study. The trim-and-fill method was applied to all identified asymmetric instances. Meta-regression was used to examine the moderating effect of MBI efficacy on biomarkers. **Results**: MBIs generally decreased the levels of inflammatory factors, such as the CRP, IL-6, TNF-α, IL-1, IL-8, IL-17, ESR, and cortisol, and increased IL-10, IFN-γ, IL-1ra, BDNF, and secretory IgA. In a subgroup analysis of the CNS and cancer, qigong and yoga showed increased BDNF and IL-6, respectively. Notably, IL-10 was increased in inflammatory diseases, and IFN-γ was increased in viral infections. **Conclusions**: This study revealed MBIs decrease inflammatory cytokine and increase anti-inflammatory, antiviral, and immune-activating factors. These results suggest the MBIs including gentle physical exercise may be beneficial for neuropsychiatric disorders or tumors. **Prospero registration number**: CRD42024507646.

## 1. Introduction

Mind–body interventions (MBIs) including mindfulness-based therapies are behavioral health interventions and mind–body practices with a focus on cultivating mind–body awareness and attitudinal qualities of kindness, compassion, joy, and equanimity [1]. MBIs include breathing exercises, meditation, and physical movements. Mindfulness-based therapies are approaches integrating mindfulness into daily life using static or dynamic methods to achieve mental and physical adjustment. Static practices include mindfulness-based stress reduction, mindfulness-based cognitive therapy, acceptance and commitment therapy, and meditation [2,3,4,5]. Dynamic practices include breathing and gentle physical exercises, such as tai chi chuan, tai chi chih, and yoga [6,7,8,9].

The immune, nervous, and endocrine systems are interconnected [10]. Chronic stress affects the immune system via the hypothalamic–pituitary–adrenal (HPA) axis and autonomic system (increased sympathetic activity and decreased parasympathetic tone). Subsequent production of proinflammatory cytokines further stimulates the HPA axis, leading to increased cortisol secretion to combat the stress [5]. Acute stress can strengthen immunity and promote protection during infection; however, chronic stress causes cortisol increase and dysregulates or inhibits immune function [11]. Chronic stress leads to chronic inflammation and disease, increased susceptibility to infections, and increased risk of cancer onset and progression [12]. Patients with chronic conditions, such as heart failure and cancer, exhibit an increased risk of mild cognitive impairment (MCI) and subsequent progression to dementia [13,14,15]. In addition to cognitive symptoms, patients with MCI often exhibit neuropsychiatric symptoms, such as depression, anxiety, apathy, and irritability [16]. A meta-analysis revealed that older adults with high circulating IL-6 levels are 1.5-times more likely to experience cognitive decline than young individuals [17]. During stress or injury, many systems are activated and transmit messages to each other [18]. Therefore, any imbalance or lack of coordination among these systems leads to immune dysregulation and disease development.

To date, most meta-analyses have focused on the immune system, with only a few studies investigating the nervous and endocrine systems [2,19]. One previous meta-analysis presented that MBIs including mindfulness-based therapy, tai chi, and yoga may reduce markers of inflammation and influence virus-specific immune responses to vaccination [20]. Another meta-analysis mentioned that mind–body interventions influence both psychological and physiological regulation, as well as the body’s reactivity to stressors, thereby decreasing the risk and progression of stress-related diseases [19]. Therefore, in this study, we aimed to identify the specific correlations among these systems and MBIs to determine the mechanisms by which different MBIs affect different health conditions.

## 2. Methods

### 2.1. Study Selection and Data Extraction

Based on the eligibility criteria outlined in the Preferred Reporting Items for Systematic Reviews and Meta-analysis (PRISMA) guidelines [21], studies reporting the effects of MBIs on at least one of the following biomarkers were selected for this meta-analysis: immune function received C-reactive protein (CRP), interleukin (IL)-6, IL-1, IL-1 receptor antagonist (IL-1ra), tumor necrosis factor (TNF)-α, IL-8, IL-10, IL-17, interferon (IFN)-γ, nuclear factor (NF)-κB, salivary immunoglobulin A (sIgA), and neuroendocrine function included brain-derived neurotrophic factor (BDNF) and cortisol. Inclusion criteria were as follows: (1) articles written in English, (2) randomized controlled trial (RCT) design, (3) including a measure of at least one of the target biomarkers, (4) published in a peer-reviewed journal, and (5) including MBIs. Exclusion criteria were as follows: (1) articles with “unpublished” status, (2) non-peer reviewed articles, (3) non-RCT design, and (4) not including a measure of at least one target biomarker. The full search protocol (registration number: CRD42024507646) can be accessed on the International Prospective Register of Systematic Reviews (http://www.crd.york.ac.uk/PROSPERO).

Three databases (PubMed, Embase, and Scopus) were searched for articles published between January, 2000 and January, 2024. Predetermined search terms related to MBI (meditation OR qigong OR yoga OR mindfulness OR mindful OR tai chi), markers (inflammatory OR cytokine OR immunity OR immune OR BDNF OR brain-derived neurotrophic factor), and design type (randomized controlled trial OR RCT) were used (Supplementary: prospero search protocol).

Two authors (SL and TC) independently conducted the electronic search. Disagreements were resolved by a third author (KY). All participant information and design data were extracted separately by the two authors to reduce the likelihood of bias. When multiple articles were published in a single study, the most relevant publication was used and supplemented with data from other relevant publications, as required. The authors of these studies were contacted when the pertinent information was unavailable in the published version.

The following data were extracted from each study by the PICO (Population, Intervention, Comparator, Outcome), which was used to define the eligibility criteria for selecting suitable studies [22]. (1) P (Population): Participants in any clinical or experimental context. No restrictions based on age, sex, or health condition were applied; (2) I (Intervention): Studies involving mind–body interventions (such as mindfulness-based stress reduction, mindfulness-based cognitive therapy, acceptance and commitment therapy, tai chi, and yoga). Refs. [2,3,4,5,6,7,8,9] and any session and duration; (3) C (Comparator): Studies involving control type (such as active control, waitlist, and treatment as usual). However, studies that compared the effects of different MBIs were excluded; (4) O (Outcome): The measurement of biomarker levels and statistics (mean, standard deviation [SD], and effect size). Most biomarkers were obtained from serum samples, except for IgA, which was obtained from saliva samples, and cortisol, which was obtained from serum, hair, and saliva samples. As hair cortisol accumulates continuously during hair growth, it has no set specific sampling time. For the serum and saliva samples, we used either the average value provided in the original study or the value from the morning collection.

### 2.2. Risk of Bias Within Individual Studies

We examined the risk of bias in the included studies according to the following six key domains using the Cochrane risk-of-bias assessment tool (Rob 2.0) [23]: (a) randomization, (b) deviation from intervention, (c) missing data, (d) measurement of outcome, (e) selection of reported results, and (f) overall risk of bias. Each potential source of bias was graded as high, low, or some concerns.

### 2.3. Summary Measures

Standardized mean difference (SMD) was calculated using primary effect size statistics. Effect sizes were calculated exclusively from the means and SDs. Pooled effect sizes were calculated separately for each biomarker at baseline and post-intervention and for the differences between the intervention and control effects.

### 2.4. Synthesis of Results

All meta-analyses and plots were estimated using the metafor package in R 4.3.0 [24]. SMD, corresponding *p* values, and 95% confidence intervals (95% CIs) were calculated. All meta-analyses were specified as random-effects models using a restricted maximum likelihood estimator. A random-effects model accounted for within- and between-study variations. Heterogeneity across the studies was assessed using I^2^ statistics. An I^2^ statistic > 50% indicated substantial heterogeneity [25]. Subgroup analyses were conducted to detect substantial heterogeneity. Based on MBI intervention type, medical condition, and control intervention type, we categorized the studies into three groups. The MBI interventions included static, qigong, and yoga. For medical conditions, the categories were central nervous system (CNS), medical disease (MD), and cancer. The control interventions included active control (AC), waitlist (WL), and treatment as usual (TAU). The static group received acceptance and compassion therapy, cognitive behavioral therapy, mindfulness-based and awareness therapy, and meditation. The qigong group included tai chi chuan, tai chi chih, and qigong. The CNS group included healthy participants experiencing stress and loneliness, patients with cognitive impairment, Parkinson’s disease, anxiety, depression, schizophrenia, or fibromyalgia. The AC group included treatment approaches other than MBI and did not simply continue with the original treatment alone, such as group counseling, health education program, psychosocial therapy, and relaxation exercise, etc. The WL group initially maintained their original lifestyle or treatment approach until the research intervention was completed, after which they joined the study treatment program. As for the TAU group, they continued maintaining their original lifestyle and treatment approach throughout the entire study.

### 2.5. Risk of Bias Across Studies

When three or more studies were identified, publication bias was assessed through visual inspection of funnel plots, where asymmetry in the distribution of effect size to standard error was suggestive of publication bias [24]. Influence diagnostics were used to assess whether pooled effects were disproportionately dependent on a single study [26]. Egger’s test was used to assess the significance of asymmetry [27]. The trim-and-fill method was applied to any identified instances of asymmetry, and the effect sizes were recalculated [28]. Meta-regression was used to examine whether the intervention method and medical conditions moderated the MBI efficacy.

## 3. Results

### 3.1. Study Selection and Characteristics

**Figure 1:**
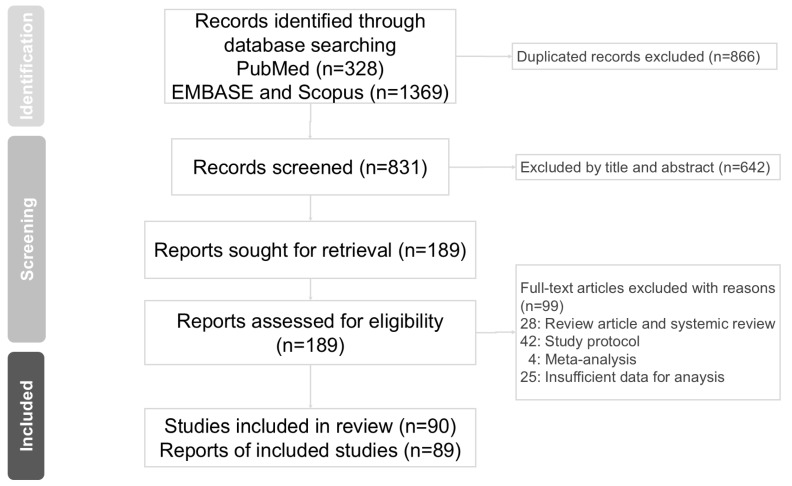
PRISMA flow diagram for studies retrieved through the electronic search and the selection processes.

In this study, we identified 1697 articles from online databases. After excluding 831 duplicated articles, 189 articles were assessed for eligibility. Finally, 89 articles [3,4,5,6,7,8,9,12,13,14,15,16,29,30,31,32,33,34,35,36,37,38,39,40,41,42,43,44,45,46,47,48,49,50,51,52,53,54,55,56,57,58,59,60,61,62,63,64,65,66,67,68,69,70,71,72,73,74,75,76,77,78,79,80,81,82,83,84,85,86,87,88,89,90,91,92,93,94,95,96,97,98,99,100,101,102,103,104,105] were assessed for suitability according to the inclusion criteria for this meta-analysis. The PRISMA flow diagram for studies retrieved through electronic searches and the selection process for study inclusion are shown in Figure 1. Characteristics of the 89 qualifying studies are presented in Table 1. The quality of the included studies was assessed using the Cochrane risk-of-bias assessment tool (Rob 2.0) [23] (Table 2). The number of studies for each biomarker varied, with 47 for CRP, 57 for IL-6, 26 for TNF-α, 10 for IL-1, 4 for IL-1ra, 13 for IL-8, 11 for IL-10, 4 for IL-17, 7 for IFN-γ, 3 for ESR, 2 for NF-κB, 11 for BDNF, 20 for cortisol, and 3 for sIgA (Table 3; Supplementary Figures S1 and S2, Supplementary Table S1).

### 3.2. Statistical Pooling of Outcomes and Meta-Analysis

A meta-analysis was conducted for each biomarker post-intervention, and the difference between the MBI and control groups was assessed. Post-intervention, MBIs slightly affected the levels of CRP (SMD = −0.12; 95% CI, −0.23 to −0.01) and IL-1ra (SMD = 0.02; 95% CI, −0.15 to 0.19), moderately affected the levels of IL-6 (SMD = −0.24; 95% CI, −0.57 to 0.08), IFN-γ (SMD = 0.32; 95% CI, −0.10 to 0.74), TNF-α (SMD = −0.37; 95% CI, −0.73 to −0.01), BDNF (SMD = 0.30; 95% CI, −0.25 to 0.85), IL-1 (SMD = −0.40; 95% CI, −0.72 to −0.08), IL-8 (SMD = −0.24; 95% CI, −0.56 to 0.08), IL-17 (SMD = −0.30; 95% CI, −0.88 to 0.28), IL-10 (SMD = 0.38; 95% CI, −0.21 to 0.97), and cortisol (SMD = −0.33; 95% CI, −0.55 to −0.12), and significantly affected the levels of sIgA (SMD = 0.59; 95% CI, −0.21 to 1.40) (Table 3; Supplementary Figure S1).

Analysis of the differences between the post-intervention effect of MBI and post-intervention effect of control groups revealed that MBIs slightly affected the levels of CRP (SMD = −0.13; 95% CI, −0.21 to −0.05), IL-6 (SMD = −0.11; 95% CI, −0.30 to 0.08), IL-8 (SMD = −0.13; 95% CI, −0.29 to 0.03), IL-1ra (SMD = −0.02; 95% CI, −0.23 to 0.19), and cortisol (SMD = −0.16; 95% CI, −0.34 to 0.03), moderately affected the levels of TNF-α (SMD = −0.24; 95% CI, −0.42 to −0.06), BDNF (SMD = 0.29; 95% CI, −0.19 to 0.78), IL-1 (SMD = −0.24; 95% CI, −0.37 to −0.11), IL-10 (SMD = 0.27; 95% CI, −0.06 to 0.61), IFN-γ (SMD = 0.46; 95% CI, −0.06 to 0.99), and sIgA (SMD = 0.39; 95% CI, −0.23 to 1.01), and significantly affected the levels of IL-17 (SMD = −0.75; 95% CI, −1.74 to 0.23) (Table 3; Supplementary Figure S3).

### 3.3. Subgroup Analysis

The I^2^ statistic showed potential heterogeneity for publication bias in IL-6, TNF-α, IL-1, IL-8, IL-10, IFN-γ, cortisol, and BDNF levels post-intervention (Table 3). Therefore, subgroup analysis was conducted.

**Figure 2:**
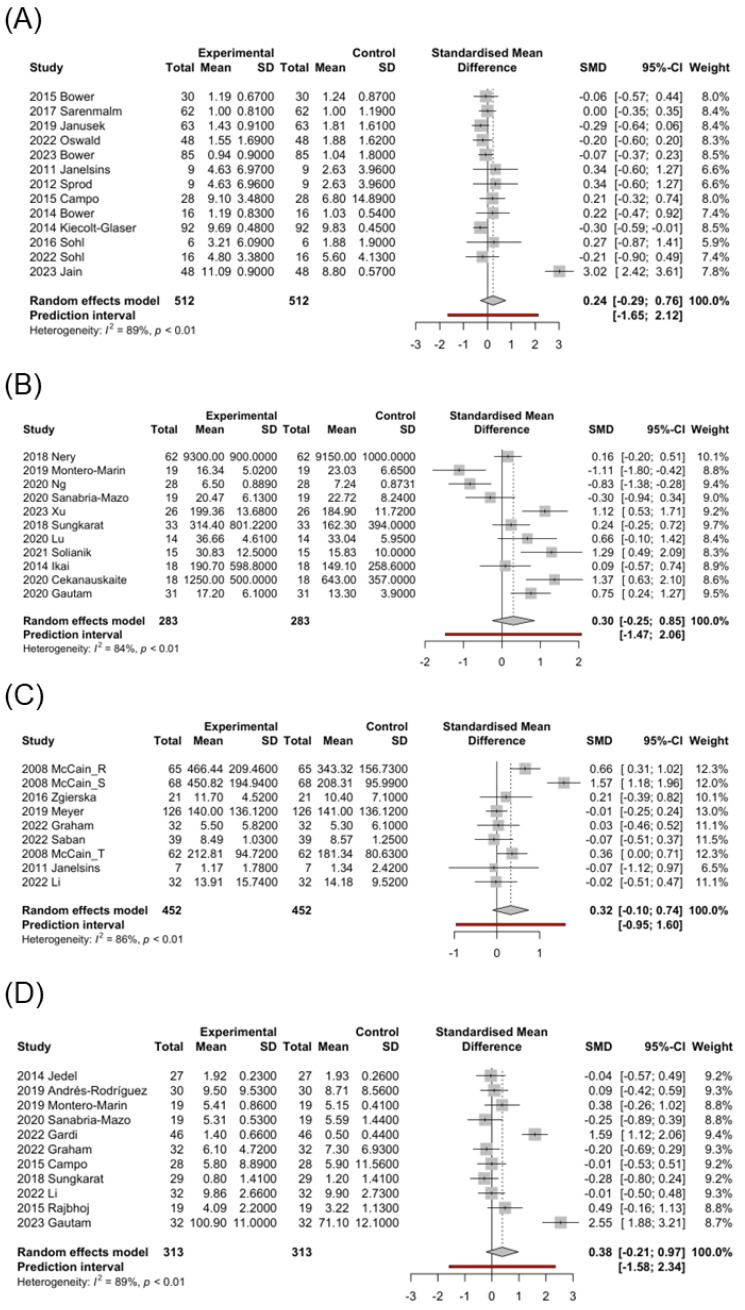
Forest plot of the standardized mean difference of mind–body interventions effect in biomarkers. (**A**) IL-6-cancer [6,8,9,38,42,44,47,49,56,91,94,96,100], (**B**) BDNF [3,15,41,51,53,61,63,66,68,78,105], (**C**) IFN-γ [5,8,30,57,83,86,92], (**D**) IL-10 [3,6,40,45,53,54,68,82,83,86,99]. Control: pre-MBI effect. Experimental: post-MBI effect.

In the MBI post-intervention studies, most groups showed a mild-to-moderate decrease in IL-6 levels, with only the cancer group showing a moderate increase in IL-6 levels (SMD = 0.24; 95% CI, −0.29 to 0.76; Figure 2A). Therefore, the cancer group was further analyzed by intervention type. Qigong (SMD = 0.29; 95% CI, 0.10 to 0.47) and yoga (SMD = 0.61; 95% CI, −1.14 to 2.36) exerted moderate-to-high increasing effects on IL-6 levels, whereas static exerted a mild decreasing effect on IL-6 levels (SMD = −0.12; 95% CI, −0.27 to 0.02). However, the control group exhibited a mild-to-moderate decrease in IL-6 levels (Table 3). We further analyzed the control post-intervention studies in the cancer group: the AC (SMD = 0.07; 95% CI, −0.30 to 0.44) exerted a mild increasing effect, and the TAU (SMD = −0.64; 95% CI, −3.08 to 1.80) exerted an obvious decreasing effect on IL-6 (Table 3).

In the MBI post-intervention on IL-6 levels in the CNS group, all groups showed a mild-to-moderate decrease. However, in the control post-intervention studies in the CNS group, the AC (SMD = 0.03; 95% CI, −0.10 to 0.16) and WL (SMD = 0.05; 95% CI, −0.11 to 0.20) exerted a mild increasing effect, and the TAU (SMD = −0.13; 95% CI, −0.48 to 0.22) exerted a moderate decreasing effect on IL-6.

In the MBI post-intervention studies in TNF-α levels, the intervention groups for static (SMD = −0.09; 95% CI, −0.34 to 0.17) and qigong (SMD = −0.01; 95% CI, −0.24 to 0.22) exhibited a mild decrease, but the group for yoga exhibited a significant decrease (SMD = −0.92; 95% CI, −1.88 to 0.03). However, when compared separately with the control group, all exhibited a mild-to-moderate decrease. In the medical condition subgroups, a significant decrease in TNF-α levels was observed in the cancer (SMD = −0.66; 95% CI, −2.42 to 1.11) and MD (SMD = −0.66; 95% CI, −1.61 to 0.30) groups, but only a slight decrease was observed in the CNS group (SMD = −0.10; 95% CI, −0.34 to 0.13) (Table 3). In the control post-intervention studies in TNF-α levels, the intervention groups for AC (SMD = −0.06; 95% CI, −0.25 to 0.13) and WL (SMD = −0.03; 95% CI, −0.67 to 0.62) exhibited a mild decrease, but the group for TAU exhibited a mild increase (SMD = 0.05; 95% CI, −0.17 to 0.27).

In the MBI post-intervention studies, a significant increase in BDNF levels was observed in the qigong (SMD = 0.70; 95% CI, −0.62 to 2.02) and yoga (SMD = 0.73; 95% CI, −0.83 to 2.29) groups, whereas a mild decrease in BDNF levels was observed in the static group (SMD = −0.18; 95% CI, −1.26 to 0.91) (Table 3). Interestingly, a mild-to-moderate increase in BDNF levels was observed in the control group. However, when comparing the post-intervention effect of MBI with the control group, the qigong (SMD = 0.68; 95% CI, −1.03 to 2.38) and yoga (SMD = 0.65; 95% CI, −0.93 to 2.24) groups exhibited a high difference, whereas the static group exhibited a slight decrease (SMD = −0.14; 95% CI, −0.92 to 0.65) (Table 3). In the control post-intervention studies, a significant increase in BDNF levels was observed in the AC (SMD = 0.59; 95% CI, −0.31 to 1.48) group, whereas a mild decrease was observed in the TAU group (SMD = −0.07; 95% CI, −0.44 to 0.30).

### 3.4. Risk of Bias Across Studies

**Figure 3:**
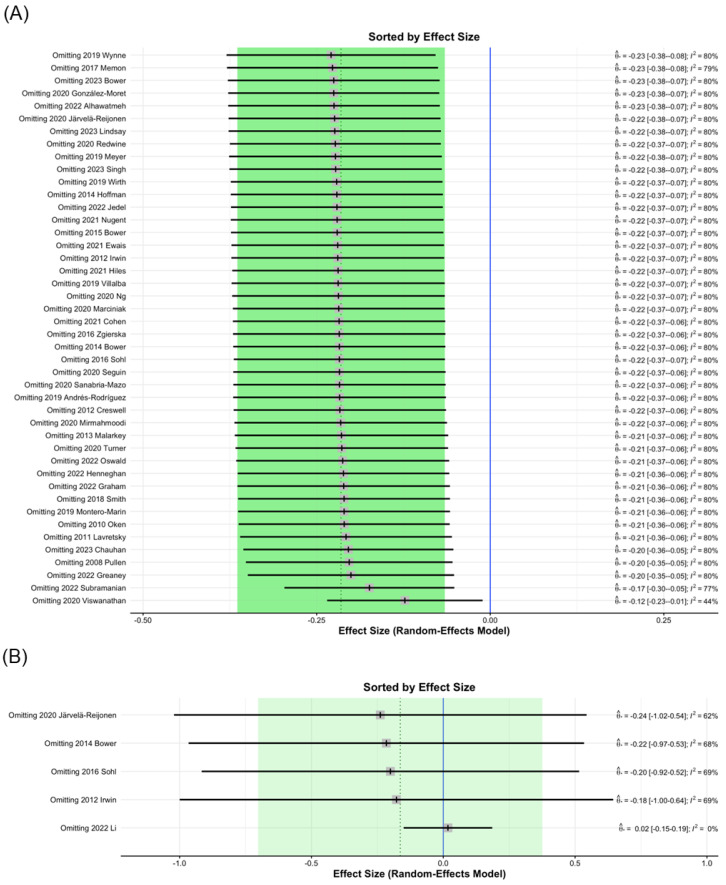
Sorting plot of influence diagnostics of biomarkers. (**A**) CRP [3,4,5,7,9,12,13,15,16,35,36,37,40,44,47,48,52,54,57,58,59,60,64,65,67,68,69,70,71,72,73,74,75,79,83,84,85,87,91,95,96,97,101,104], (**B**) IL-1ra [9,37,47,65,86].

Influence diagnostics identified 2020 Viswanathan [71] as a study that, when excluded from analysis, contributed to significant changes in the MBI post-intervention study I^2^ values of CRP from 80% to 44% (Figure 3A); therefore, the data of 2020 Viswanathan was removed. This finding may be attributed to the relatively large number of participants. In the analysis of IL-1ra levels, the 2022 Li study [86], which was excluded from analysis, contributed to significant changes in I^2^ values from 60% to 0% (Figure 3B). This result may be due to the testing being conducted six months after MBI treatment.

Results from the I^2^ statistic show the potential for publication bias for CRP, IL-6, TNF-α, IL-10, IFN-γ, BDNF, and cortisol levels at post-intervention (Table 3; Supplementary Figure S4). The trim-and-fill method was used to estimate the effect sizes of potentially suppressed studies [28]. This did not alter the parameter estimates for IL-6, IL-10, BDNF, and cortisol levels, as no supplementary studies were included (Table 4; Supplementary Figure S5). However, the trim-and-fill method results increased the I^2^ statistic of the CRP (post-intervention) from 44% to 65%. Heterogeneity at post-intervention was moderate-to-high for CRP, IL-6, IL-6-CNS, IL-6-cancer, TNF-α, IL-10, IFN-γ, BDNF, and cortisol (Table 4). However, in the subgroup analysis, the number of studies may be smaller, which could lead to small-study effects-related heterogeneity.

### 3.5. Meta-Regression

Next, meta-regression was performed to account for between-study sources of heterogeneity using study-specific characteristics. In trim-and-fill analysis, the potentially missing studies for subgroups of CRP, IL-6-CNS, TNF-α, and IFN-γ were noted. However, the subgroup meta-regression based on intervention types did not reveal statistically significant effects, indicating that the observed heterogeneity may primarily stem from publication bias rather than differences in intervention methods (Table 4). Moderators of the intervention type showed a significant association with IL-6 levels in the cancer subgroup (Q = 37.52, *p* < 0.001; Table 4). This may also reflect the impact of the intervention method on cytokine IL-6 in the cancer subgroup.

## 4. Discussion

Bidirectional communication is observed between the neuroendocrine and immune systems, which is essential to maintain physiological homeostasis and good health. The hormonal and neuropeptide mediators that connect the endocrine, central nervous, and immune systems form distinct interaction pathways, such as the hypothalamic–pituitary–adrenal (HPA) axis and the autonomic nervous system [18]. Chronic stress may cause immune and endocrine system dysregulation, then chronic inflammation and pathological outcomes are further exacerbated. MBIs can regulate psychological and physiological reactivity to stressors, decrease sympathetic tone, and increase parasympathetic activity, thereby reducing the likelihood and progression of stress-related disease [19]. MBIs affect the physiological markers integral to immune function, such as those involved in inflammation (like CRP and IL-6) and improve the response to infection [106]. Here, MBIs generally decreased the levels of inflammatory factors, such as CRP, IL-6, TNF-α, IL-1, IL-8, IL-17, ESR, and cortisol, and increased the levels of anti-inflammatory, antiviral, and immune-activating factors, such as IL-10, IFN-γ, IL-1ra, BDNF, and sIgA.

Biomarkers of stress, inflammation, and neuroplasticity are implicated in MCI [15]. Some biomarkers, such as CRP, IL-6, and IL-1β, related to aging or diseases, such as heart failure and cancer, are associated with MCI [13,107]. BDNF is a neurotrophin essential for neurogenesis and maintenance of neuronal plasticity, and its expression is influenced by epigenetic changes and upstream regulators, such as pro-inflammatory cytokines [107,108]. In a study by Devasahayam et al. [109], no differences were reported in BDNF levels at rest between patients with multiple sclerosis (MS) and controls; however, IL-6 levels were significantly higher in patients with MS. In the MS group, increased BDNF/IL-6 ratio was associated with fast walking speed exercise. Here, under the influence of MBIs, the qigong (SMD = 0.70; 95% CI, −0.62 to 2.02) and yoga (SMD = 0.73; 95% CI, −0.83 to 2.29) groups showed significantly increased BDNF levels, whereas the static group exhibited no significant difference or a slight decrease in BDNF levels (SMD = −0.18; 95% CI, −1.26 to 0.91) (Table 3). Interestingly, the control group showed a mild overall increase in BDNF levels, and the subgroup of AC (SMD = 0.59; 95% CI, −0.31 to 1.48) exhibited a high increase, possibly because patients in the AC group received supportive treatment including relaxation exercise, group counseling, and cognitive training. This implies that a combination of physical and gentle exercise, along with active psychological support, positively affects the BDNF levels (Figure 2B).

Here, in the subgroup analysis of IL-6, the CNS subgroup exhibited consistently decreased IL-6 levels (SMD = −0.19; 95% CI, −0.34 to −0.03; Supplementary Figure S1C), whereas the control group showed increasing IL-6 levels (SMD = 0.03; 95% CI, −0.08 to 0.15) (Table 3). Additionally, when we further analyzed this CNS subgroup according to different types of MBIs individually, we found that IL-6 levels were always decreased regardless of the type of MBI. However, in the control post-intervention studies in the CNS group on IL-6, the AC (SMD = 0.03; 95% CI, −0.10 to 0.16) and WL (SMD = 0.05; 95% CI, −0.11 to 0.20) exerted a mild increasing effect, and the TAU (SMD = −0.13; 95% CI, −0.48 to 0.22) exerted a moderate decreasing effect on IL-6. This may imply that MBI can reduce pro-inflammatory cytokine IL-6 in patients with neurological and psychosomatic symptoms, whereas other exercise methods without MBI may not necessarily have the same effect.

Additionally, IL-6 levels were increased in the cancer subgroup (SMD = 0.24; 95% CI, −0.29 to 0.76; Figure 2A). To further understand the influence of MBIs on this subgroup, we conducted individual analyses of different types of MBIs in the cancer subgroup. Both the qigong (SMD = 0.29; 95% CI, 0.10 to 0.47) and yoga (SMD = 0.61; 95% CI, −1.14 to 2.36) groups exhibited increased IL-6 levels, and only the static group showed a slight decrease in IL-6 levels (SMD = −0.12; 95% CI, −0.27 to 0.02). In contrast, the control group exhibited decreased IL-6 levels (Table 3). In the subgroup analysis, the AC (SMD = 0.07; 95% CI, −0.30 to 0.44) exerted a mild increasing effect, and the TAU (SMD = −0.64; 95% CI, −3.08 to 1.80) exerted an obvious decreasing effect on IL-6. This shows that exercise can indeed increase IL-6 levels in cancer patients, but MBI combined with gentle exercise has an even more significant effect in this participant group.

IL-6 is a cytokine abundantly expressed in the tumor microenvironment of various tumor types [110]. Direct stimulation of tumor cells via IL-6 leads to increased cell proliferation and invasiveness. IL-6 is produced by multiple cell types in the tumor microenvironment, including tumor-infiltrating immune cells, stromal cells, and tumor cells themselves. Paracrine or autocrine IL-6 signaling prompts stromal and immune cells to secrete signaling molecules, such as the vascular endothelial growth factor (VEGF) for angiogenesis or pro-inflammatory cytokine IL-1β [111]. As no increase in IL-6 levels was observed in the static group in this study, the increase in IL-6 levels may not be due to the impact of the tumor.

Some studies have reported the anti-inflammatory effects of exercise [10,112]. At rest, approximately 30% of circulating IL-6 levels are attributed to the adipose tissue, with only approximately 10% attributed to adipocytes, and the remainder are mostly attributed to adipose tissue-resident macrophages. Other sources of circulating IL-6 include blood leukocytes (predominantly monocytes), brain, and liver [10]. The plasma level of IL-6 increases exponentially with exercise duration and returns to resting levels within 1 h of exercise [8,113,114]. In our analysis, the qigong and yoga groups showed a significant increase in IL-6 levels. In contrast, the control group showed a decrease in IL-6 levels. In the study by Campo et al. [6] on 28 cancer survivors, IL-6 levels were significantly increased from the baseline 6.8 (SD 14.89) pg/mL to 1-week post-intervention 9.1 (SD 3.48) pg/mL after 12 weeks of tai chi training. This indicates that treatment combining MBIs with gentle physical exercise continuously increases the IL-6 levels in patients with cancer. Regular exercise reduces the resting levels of intratumoral IL-6 correlating with a reduced tumor size in breast cancer-bearing mice [115]. Exercise-induced acute release of IL-6 promotes antitumor adaptive immunity by inducing the migration of cytotoxic T cells to tumor-draining lymph nodes and tumor vasculature and stimulating lymphocyte trafficking [116,117].

IFN-γ is the only type II interferon produced by natural killer and T cells that is an important antiviral cytokine and activator of macrophages [118]. It promotes T cell activation, Th1 differentiation, and production of pro-inflammatory cytokines (particularly TNF) [119]. Here, patients receiving MBIs showed a moderate increase in IFN-γ levels (SMD = 0.32; 95% CI, −0.10 to 0.74; Figure 2C), whereas the control group exhibited decreased IFN-γ levels (SMD = −0.17; 95% CI, −0.54 to 0.20) (Table 3). In the study by McCain et al. [30] on 62 patients undergoing treatment for HIV infection, IFN-γ levels were significantly increased from 181.34 (SE 10.24) pg/mL to 212.81 (SE 12.03) pg/mL after 10 weeks of tai chi training but significantly decreased from 599.51 (SE 38.66) pg/mL to 366.94 (SE 23.31) pg/mL in the control group, showing a marked difference. This suggests that IFN-γ levels are increased by MBI therapy, initiating an immune response to combat viral infections.

IL-10 is a cytokine with potent anti-inflammatory properties that plays key roles in immune and inflammatory responses [120]. In this analysis, MBIs moderately increased the IL-10 levels (SMD = 0.38; 95% CI, −0.21 to 0.97; Figure 2D). Subgroup analysis showed the significant impact on patients with inflammatory diseases, such as rheumatoid arthritis [99] and ulcerative colitis [40], but no noticeable effects on patients with cancer [6] (Table 3). This suggests that MBIs increase the anti-inflammatory IL-10 levels in inflammatory conditions. However, this effect is not as pronounced in non-inflammatory conditions.

Physiological responses to stress can be evaluated using cortisol and sIgA levels [39]. Here, stress-prone populations, such as young students or nurses, exhibited decreased cortisol levels (SMD = −0.33; 95% CI, −0.55 to −0.12; Supplementary Figure S1J) and increased sIgA levels (SMD = 0.59; 95% CI, −0.21 to 1.40; Supplementary Figure S1K) after their participation in the research project. This trend can be observed regardless of whether they received MBI therapy. However, patients who underwent MBI therapy exhibited moderate-to-high changes in these biomarker levels.

This study has some limitations. First, although we observed that MBIs increase the circulatory IL-6 levels in cancer, whether they exert inhibitory effects on tumors or prevent their recurrence warrants further cohort studies. Additionally, whether and for how long changes in these biomarkers are sustained following interventions remain unclear. In the analysis results, some small effect sizes can be observed. However, the long-term cumulative effect of MBI on these biomarkers may still have a significant impact. Third, whether IL-6 serves as a primary assessment tool for outcome measures in cancer patients undergoing MBI therapy remains unclear. Future research should focus on longitudinal studies to determine whether these changes in biomarkers lead to meaningful clinical outcomes.

## 5. Conclusions

We found that MBIs generally decrease the inflammatory cytokine levels and increase the anti-inflammatory and antiviral cytokine levels. In the subgroup analysis of CNS, qigong and yoga increased the levels of BDNF but decreased IL-6; however, in the subgroup analysis of cancer, the levels of IL-6 were increased. These effects respectively imply neuroprotection and tumor suppression. Additionally, MBI may elevate the IFN-γ and sIgA levels during the condition of virus infection and under stress. In inflammatory diseases, such as rheumatoid arthritis and ulcerative colitis, MBIs significantly increase the levels of anti-inflammatory cytokine IL-10.

Disease and stress cause an imbalance in the immune and neuroendocrine systems. MBIs may restore the balance among these systems, and help the body from external stress, infections, and internal tumors. The MBIs including gentle physical exercise may be beneficial for neuropsychiatric disorders and tumors.

## Figures and Tables

**Table 1:** Characteristics of the included studies.

First Author Year	Medical Condition	MBI (Duration, Session)	Control (Duration, Session)	Biomarker	MBI (n)	Ctrl (n)
2006 Chen [29]	Osteoporosis	Qigong (12 wk, 8 session)	Ctrl (12 wk)	IL-6	44	43
2008 McCain [30]	HIV stress	Tai Chi (10 wk, 10 session)	WL (10 wk)	IFN-γ, salivary cortisol	62	57
		RLXN (10 wk, 10 session)			65	
		SPRT (10 wk, 10 session)			68	
2008 Pullen [7]	Heart failure	Yoga (Hatha and Pranayama) (8 wk, 16 session)	TAU (8 wk)	CRP, IL-6	9	10
2009 Banasik [31]	Breast cancer	Yoga (8 wk, 16 session)	TAU (8 wk)	salivary cortisol	7	7
2010 Fan [32]	Healthy student	IBMT (4 wk, every night)	RT (4 wk, every night)	salivary IgA	17	18
2010 Oh [33]	Cancer	Qigong (10 wk, 20 session)	TAU (10 wk)	CRP	79	83
2010 Oken [4]	Alzheimer’s care givers	MBCT (6 wk, every day)	PTC (6 wk, every day)	CRP, IL-6, TNF-α, salivary cortisol	10	10
2010 Pullen [34]	Heart failure	Yoga (Asana, Pranayama) (8 wk, 16 session)	TAU (8 wk)	CRP, IL-6	21	19
2011 Janelsins [8]	Breast cancer	TCC (12 wk, 36 session)	PST (12 wk, 36 session)	IL-6, IFN-γ	9	10
2011 Lavretsky [35]	Depression	TCC (10 wk, 10 session)	HE (10 wk, 10 session)	CRP	33	35
2012 Creswell [36]	Lonely elderly	MBSR (8 wk, 8 session)	WL (8 wk)	CRP, IL-6	20	20
2012 Irwin [37]	Elderly	TCC (16 wk, 48 session)	HE (16 wk, 48 session)	CRP, IL-6, IL-1ra	46	37
2012 Oh [14]	Cancer	Qigong (10 wk, 20 session)	TAU (10 wk)	CRP	23	31
2012 Sprod [38]	Breast cancer	TCC (12 wk, 36 session)	SST (12 wk, 36 session)	IL-6, IL-8, serum cortisol	9	10
2013 Chan [39]	Stressed nurse	Qigong (10 wk, 20 session)	Ctrl (10 wk)	salivary cortisol and IgA	18	16
2013 Malarkey [12]	Stressed faculty	Mindful (8 wk, 8 session)	LE (8 wk, 8 session)	CRP, IL-6, salivary cortisol	84	85
2014 Bower [9]	Breast cancer	Yoga (Lyengar system) (12 wk, session unmentioned)	Ctrl (12 wk, session unmentioned)	CRP, IL-6, IL-1ra	16	15
2014 Jedel [40]	Ulcerative colitis	MBSR (8 wk, 6 days/wk)	Ctrl (8 wk)	CRP, IL-6, IL-8, IL-10	27	28
2014 Ikai [41]	Schizophrenia	Yoga (Hatha) (8 wk, 8 session)	TAU (8 wk)	BDNF	18	18
2014 Kiecolt-Glaser [42]	Breast cancer	Yoga (Hatha) (12 wk, 24 session)	WL (12 wk)	IL-6, IL-1, TNF-α	92	80
2015 Black [43]	Sleep disturbance	MAP (6 wk, 6 session)	SHE (6 wk, 6 session)	NF-κB	24	25
2015 Bower [44]	Breast cancer	MAP (6 wk, 6 session)	WL (6 wk)	CRP, IL-6	30	22
2015 Campo [6]	Cancer elderly	TCC (12 wk, 36 session)	HE (12 wk, 36 session)	IL-6, IL-10, TNF-α	28	24
2015 Rajbhoj [45]	Heathy participant	Yoga (12 wk, 6 days/wk)	TAU (12 wk)	IL-1, IL-10	19	18
2016 Creswell [46]	Stress adult	MBSR (3 day)	GC (3 day)	IL-6	18	17
2016 Sohl [47]	GI cancer	Yoga (8 wk, 4 session)	AC (8 wk, 4 session)	CRP, IL-6, IL-1ra, TNF-α	6	5
2016 Zgierska [5]	Low back pain	Meditation (8 wk, 8 session)	WL (8 wk)	CRP, IL-6, IL-1, IFN-γ, TNF-α	21	14
2017 Memon [48]	Depression	MBSR (8 wk, session unmentioned)	CBT (8 wk, session unmentioned)	CRP, IL-8	81	85
2017 Sarenmalm [49]	Breast cancer	MBSR (8 wk, 8 session)	Ctrl (8 wk)	IL-6, IL-8	62	52
2018 Hoge [50]	Anxiety	Mindful (8 wk, 8 session)	SME (8 wk, 8 sesion)	IL-6, TNF-α, serum Cortisol	42	28
2018 Nery [51]	Infertility woman	MBP (8 wk, 8 session)	Ctrl (8 wk)	BDNF, hair cortisol	62	37
2018 Smith [52]	Obesity	Mindful (6 wk, 6 session)	GC (6 wk, 6 session)	CRP, IL-6	18	18
2018 Sungkarat [53]	MCI	Tai Chi (3 wk, 9 session) (assess at 6 mo)	Ctrl (3 wk)	IL-10, TNF-α, BDNF	29	27
2019 Andrés-Rodríguez [54]	Fibromyalgia	MBSR (8 wk, 8 session)	TAU (8 wk)	CRP, IL-6, IL-10	30	27
2019 Cheung [55]	Stressed women	Qigong (22 wk, 27 session)	WL (22 wk)	IL-6, TNF	136	135
2019 Janusek [56]	Breast cancer	MBSR (8 wk, 8 session)	AC (8 wk, 8 session)	IL-6, TNF-α	63	61
2019 Meyer [57]	Healthy	MBSR (8 wk, 8 session)	WL (8 wk)	CRP, IL-6, IFN-γ	126	130
2019 Montero-Marin [3]	Fibromyalgia	ABCT (8 wk, 8 session)	RT (8 wk, 8 session)	CRP, IL-6, IL-10, TNF-α, BDNF	19	15
2019 Villalba [58]	Stressed adult	MAT (8 wk) (2 wk smartphone intervention)	Ctrl (8 wk)	CRP	58	36
2019 Wirth [59]	Cancer	MBSR (4 wk, 4 session)	BC (4 wk, every day)	CRP	19	17
2019 Wynne [60]	IBD	ACT (8 wk, 8 session)	Ctrl (8 wk)	CRP	37	42
2020 Cekanauskaite [61]	Elderly	Yoga (Asana, Himalayan) (10 wk, 20 session)	Ctrl (10 wk)	BDNF	18	15
2020 Ganesan [62]	RA	Yoga (Asana, Pranayama) (12 wk, 36 session)	Ctrl (12 wk)	IL-6, IL-1, TNF-α, serum cortisol	68	75
2020 Gautam [63]	RA	Yoga (Asana, Pranayama, Dhyana, Savasana) (8 wk, 40 session)	Ctrl (8 wk)	IL-6, IL-17, TNF-α, BDNF	31	31
2020 González-Moret [64]	IBD	Mindfulness (8 wk, 4 session) (assess at 6 mo)	TAU (8 wk)	CRP, hair cortisol	37	20
2020 Järvelä-Reijonen [65]	Mental distressed	ACT (8 wk, 6 session)	Ctrl (8 wk)	CRP, IL-1ra, serum cortisol	57	58
2020 Lu [66]	Depression	Qigong (12 wk, 24 session)	CT (12 wk, 24 session)	BDNF, salivary cortisol	14	16
2020 Marciniak [16]	MCI	MBSR (8 wk, 8 session)	CT (8 wk, 8 session)	CRP, IL-6, TNF-α	12	8
2020 Mirmahmoodi [67]	Breast cancer	MBSR (8 wk, 8 session)	TAU (8 wk)	CRP, serum cortisol	22	22
2020 Ng [15]	MCI	MAP (12 wk, 12 session)	HEP (12 wk, 12 session)	CRP, IL-6, IL-1, BDNF, salivary cortisol	28	27
2020 Redwine [13]	Heart failure, MCI	Tai Chi (16 wk, 32 session)	TAU (16 wk)	CRP, IL-6, TNF-α	18	18
2020 Sanabria-Mazo [68]	Fibromyalgia	MAIR (8 wk, 8 session)	RT (8 wk, 8 session)	CRP, IL-6, IL-10, TNF-α, BDNF	19	15
2020 Seguin [69]	Chronic pain	Yoga (Hatha) (12 wk, 24 session)	Ctrl (12 wk)	CRP	19	18
2020 Turner [70]	Stressed student	MBP (8 wk, session unmentioned)	TAU (8 wk)	CRP, TNF-α, IL-8, serum cortisol	22	25
2020 Viswanathan [71]	Type2 DM	Yoga (Yogasana) (12 wk, 5 days/wk)	SPE (12 wk, 5 days/wk)	CRP, IL-6, TNF-α	150	150
2021 Cohen [72]	Early life stress	MBSR (4 wk, 8 session)	Ctrl (4 wk)	CRP, IL-6, salivary cortisol	21	17
2021 Ewais [73]	IBD	MBCT (8 wk, 8 session)	TAU (8 wk)	CRP, ESR, IL-6	33	31
2021 Hiles [74]	Asthma	Yoga (16 wk, 16 session)	AC (16 wk, 8 session)	CRP	13	8
2021 Nugent [75]	MDD	Yoga (Hatha) (10 wk, 20 session)	HE (10 wk, 10 session)	CRP, IL-6, TNF-α	48	39
2021 Oliveira [76]	Stressed teachers	MBP (8 wk, 8 session)	NEP (8 wk, 8 session)	IL-6, IL-8	21	20
2021 Qi [77]	Elderly	Qigong (12 wk, 18 session)	SPE (12 wk, 18 session)	IL-6	22	26
2021 Solianik [78]	Stressed elderly	Tai Chi (10 wk, 5 session)	Ctrl (10 wk)	BDNF	15	15
2022 Alhawatmeh [79]	Stressed student	Meditation (5 wk, 5 session)	Ctrl (5 wk, 5 session)	CRP, serum cortisol	54	54
2022 Chanta [80]	Allergic rhinitis	Yoga (Hatha) (8 wk, 24 session)	TAU (8 wk)	IL-6	13	14
2022 Diez [81]	Chronic back pain	MBSR (8 wk, 9 session)	Ctrl (8 wk)	IL-6, IL-1, IL-17, TNF-α	30	32
2022 Gardi [82]	Healthy	MBP (3 day)	AC (3 day)	IL6, IL8, IL10, salivary cortisol	46	44
2022 Graham [83]	Nurse	MBSR (6 wk, 6 session)	Music (6 wk, 5 min/day)	CRP, IL-6, IL-8, IL-10, TNF-α, IFN-γ	32	29
2022 Greaney [84]	Breast cancer	Yoga (12–20 wk, 3 times/wk)	Ctrl (12–20 wk)	CRP	13	12
2022 Henneghan [85]	CRCI	Meditation (8 wk, 12 min/daily)	Music (8 wk, 12 min/daily)	CRP	13	12
2022 Li [86]	Parkinson’s disease	Tai Chi (6 mo, session unmentioned)	Ctrl (6 mo)	IL-6, IL-1, IL-1ra, IL-8, IL-10, IL-17, IFN-γ, TNF-α	32	32
2022 Jedel [87]	Ulcerative colitis	Mindful (8 wk, 8 session)	AC (8 wk, 8 session)	CRP, IL-6, IL-8	20	23
2022 Lindsay [88]	Lonely elderly	MBSR (8 wk, 8 session)	HEP (8 wk, 8 session)	IL-6	89	93
2022 Martínez-Borrás [89]	Stressed work	MSCBI (6 wk, 6 session)	WSMI (6 wk, 3 session	salivary IgA	13	11
2022 Ng [90]	Depression and insomnia	IBMS (8 wk, 8 session)	WL (8 wk)	IL-1, IL-6	93	93
		Qigong (8 wk, 3 times/wk)			95	93
2022 Oswald [91]	Cancer	MBSR (8 wk, 8 session)	WL (8 wk)	CRP, IL-6	48	49
2022 Saban [92]	Stressed women	MBSR (8 wk, 8 session)	HEP (8 wk, 8 session)	IL-6, IFN-γ, salivary cortisol	39	34
2022 Sharma [93]	Obesity	YBLI (12 wk, 6 days/wk)	PE (12 wk, 5 days/wk)	IL-6, TNF-α, NF-κB	34	31
2022 Sohl [94]	GI cancer	Yoga (8 wk, 4 session)	AC (8 wk, 4 session)	IL-6, TNF-α	16	13
2022 Subramanian [95]	COVID-19	Meditation (4 wk, 8 session)	Relax (4 wk, 8 session)	CRP, serum cortisol	22	22
2023 Bower [96]	Breast cancer	MAP (6 wk, 6 session)	TAU (6 wk, 6 session)	CRP, IL-6	85	81
2023 Chauhan [97]	Varicose vein	Yoga (Asana, Pranayama, Dhyana) (4 wk, 5 days/wk)	PE (4 wk, 5 days/wk)	CRP	23	23
2023 Dua [98]	COVID-19	Yoga (Pranayama, Gayatri mantra) (2 wk, daily)	TAU (2 wk)	CRP, ESR, IL-6	9	9
2023 Gautam [99]	RA	Yoga (Asanas, Pranayama, Dhyna, Savasna) (8 wk, 5 times/wk)	TAU (8 wk)	IL-6, IL-10, IL-17	32	32
2023 Jain [100]	Breast cancer	Yoga (Asanas) (16 wk, 5 days/wk)	Ctrl (16 wk)	IL-6, IL-8	48	48
2023 Lindsay [101]	Lonely elderly	MBSR (8 wk, 8 session)	HEP (8 wk, 8 session)	CRP, IL-6	92	95
2023 Liu [102]	COPD	Tai Chi (8 wk, 24 session)	Ctrl (8 wk)	IL-6, IL-8, TNF-α	26	26
2023 Mullapudi [103]	Schizophrenia	Yoga (Asanas, Pranayama) (6 mo) (20 session in 1st month, then 3 days/wk for 5 months)	TAU (6 mo)	TNF-α	21	20
2023 Singh [104]	AS	Yoga (Asanas, Pranayama, Dhyhana) (12 wk, 24 session)	TAU (12 wk)	CRP, ESR	57	52
2023 Xu [105]	MDD	MBCT (8 wk, 8 session)	GC (8 wk, 8 session)	IL-6, IL-1, IL-8, TNF-α, BDNF	26	22

Medical condition: AS: Ankylosing spondylitis; CRCI: cancer-related cognitive impairment; GI cancer: gastrointestinal cancer; IBD: inflammatory bowel disease; MCI: mild cognitive impairment; Mind–body intervention: ABCT: attachment-based compassion therapy; ACT: acceptance and commitment therapy; CBT: Cognitive Behavioral Therapy; IBMS: Integrative Body–Mind–Spirit; IBMT: Integrative Body–Mind Training; MAIR: mindfulness plus amygdala and insula retraining; MAP: Mindful Awareness Practices; MAT: monitor and acceptance theory; MBCT: Mindfulness-Based Cognitive Therapy; MBP: Mindfulness-Based Program; MBSR: Mindfulness-Based Stress Reduction; MSCBI: Mindfulness and Self-Compassion-Based Intervention; RLXN: Cognitive–Behavioral Relaxation Training; SPRT: Spiritual Growth; TCC: Tai Chi Chuan or Tai Chi Chih; YBLI: yoga-based lifestyle intervention; Control method: AC: Attention Control, Active Control; BC: Breathing Control; Ctrl: control; CT: Cognitive Training; GC: group counseling; HE: Health Education; HEP: health education program; LE: Lifestyle Education; NEP: Neuroeducation Program; PE: Physical Exercise; PST: Psychosocial Therapy; PTC: Power Tools for Caregivers; RT: Relaxation Training or Relaxation Therapy; SST: Standard Support Therapy; SHE: Sleep Hygiene Education; SME: Stress Management Education; SPE: simple physical exercise; TAU: treatment as usual; WL: waitlist control; WSMI: Workplace Stress Management Intervention; Biomarker Neuroendocrine function; BDNF: Brain-derived Neurotrophic Factor Serum and salivary cortisol immune function; CRP: C-reactive protein IFN-γ: interferon-γ; IL-1ra: interleukin-1 receptor antagonist; IL-6: interleukin-6; IL-8: interleukin-8; IL-10: interleukin-10; IL-17: interleukin-17; NF-κB: nuclear factor-κB; salivary IgA: salivary immunoglobulin A; TNF-α: tumor necrosis factor-α.

**Table 2:** Risk of bias for each study. Domains, D1: Bias arising from the randomization process; D2: Bias due to deviations from intended interventions; D3: Bias due to missing outcome data; D4: Bias in measurement of the outcome; D5: Bias in selection of the reported result; D6: Overall risk of bias.

Report	D1	D2	D3	D4	D5	Overall RoB
2006 Chen [29]	Low	Low	Low	Low	Low	Low
2008 McCain [30]	Low	Low	Low	Low	Low	Low
2008 Pullen [7]	Low	Low	Low	Low	Low	Low
2009 Banasik [31]	Low	Low	Low	Low	Low	Low
2010 Fan [32]	Low	Low	Low	Low	Low	Low
2010 Oh [33]	Low	Low	Low	Low	Low	Low
2010 Oken [4]	Low	Low	Low	Low	Low	Low
2010 Pullen [34]	Low	Low	Low	Low	Low	Low
2011 Janelsins [8]	Low	Low	Low	Low	Low	Low
2011 Lavretsky [35]	Low	Low	Low	Low	Low	Low
2012 Creswell [36]	Low	Low	Low	Low	Low	Low
2012 Irwin [37]	Low	Low	Low	Low	Low	Low
2012 Oh [14]	Low	Low	Low	Low	Low	Low
2012 Sprod [38]	Low	Low	Low	Low	Low	Low
2013 Chan [39]	Low	Low	Low	Low	Low	Low
2013 Malarkey [12]	Low	Low	Low	Low	Low	Low
2014 Bower [9]	Low	Low	Low	Low	Low	Low
2014 Jedel [40]	Low	Low	Low	Low	Low	Low
2014 Ikai [41]	Low	Low	Low	Low	Low	Low
2014 Kiecolt-Glaser [42]	Low	Low	Low	Low	Low	Low
2015 Black [43]	Low	Low	Low	Low	Low	Low
2015 Bower [44]	Low	Low	Low	Low	Low	Low
2015 Campo [6]	Low	Low	Low	Low	Low	Low
2015 Rajbhoj [45]	Low	Low	Low	Low	Low	Low
2016 Creswell [46]	Low	Low	Low	Low	Low	Low
2016 Sohl [47]	Low	Low	Low	Low	Low	Low
2016 Zgierska [5]	SC	Low	Low	SC	Low	SC
2017 Sarenmalm [49]	Low	Low	Low	Low	Low	Low
2017 Memon [48]	Low	Low	Low	Low	Low	Low
2018 Hoge [50]	Low	Low	Low	Low	Low	Low
2018 Nery [51]	Low	Low	Low	SC	Low	SC
2018 Smith [52]	SC	Low	Low	Low	Low	SC
2018 Sungkarat [53]	Low	Low	Low	Low	Low	Low
2019 Andrés-Rodríguez [54]	Low	Low	Low	Low	Low	Low
2019 Cheung [55]	Low	Low	Low	Low	Low	Low
2019 Janusek [56]	Low	Low	Low	Low	Low	Low
2019 Meyer [57]	Low	Low	Low	Low	Low	Low
2019 Montero-Marin [3]	Low	Low	Low	Low	Low	Low
2019 Villalba [58]	Low	Low	Low	Low	Low	Low
2019 Wirth [59]	Low	Low	Low	Low	Low	Low
2019 Wynne [60]	Low	Low	Low	Low	Low	Low
2020 Cekanauskaite [61]	Low	Low	Low	SC	Low	SC
2020 Ganesan [62]	Low	Low	Low	Low	Low	Low
2020 Gautam [63]	Low	Low	Low	Low	Low	Low
2020 González-Moret [64]	Low	Low	Low	Low	Low	Low
2020 Järvelä-Reijonen [65]	Low	Low	Low	Low	Low	Low
2020 Lu [66]	Low	Low	Low	Low	Low	Low
2020 Marciniak [16]	SC	Low	Low	Low	Low	SC
2020 Mirmahmoodi [67]	Low	Low	Low	Low	Low	Low
2020 Ng [15]	Low	Low	Low	Low	Low	Low
2020 Redwine [13]	Low	Low	Low	Low	Low	Low
2020 Sanabria-Mazo [68]	Low	Low	Low	Low	Low	Low
2020 Seguin [69]	Low	Low	Low	Low	Low	Low
2020 Turner [70]	Low	Low	Low	Low	Low	Low
2020 Viswanathan [71]	Low	Low	Low	Low	Low	Low
2021 Cohen [72]	Low	Low	Low	Low	Low	Low
2021 Ewais [73]	Low	Low	Low	Low	Low	Low
2021 Hiles [74]	Low	Low	Low	Low	Low	Low
2021 Nugent [75]	Low	Low	Low	Low	Low	Low
2021 Oliveira [76]	Low	Low	Low	SC	Low	SC
2021 Qi [77]	SC	Low	Low	Low	Low	SC
2021 Solianik [78]	Low	Low	Low	SC	Low	SC
2022 Alhawatmeh [79]	Low	Low	Low	Low	Low	Low
2022 Chanta [80]	Low	Low	Low	SC	Low	SC
2022 Diez [81]	Low	Low	Low	Low	Low	Low
2022 Gardi [82]	Low	Low	Low	Low	Low	Low
2022 Graham [83]	Low	Low	Low	SC	Low	SC
2022 Greaney [84]	Low	Low	Low	SC	Low	SC
2022 Henneghan [85]	Low	Low	Low	SC	Low	SC
2022 Li [86]	Low	Low	Low	SC	Low	SC
2022 Jedel [87]	Low	Low	Low	Low	Low	Low
2022 Lindsay [88]	SC	Low	Low	Low	Low	SC
2022 Martínez-Borrás [89]	Low	Low	Low	Low	Low	Low
2022 Ng [90]	Low	Low	Low	Low	Low	Low
2022 Oswald [91]	Low	Low	Low	Low	Low	Low
2022 Saban [92]	Low	Low	Low	Low	Low	Low
2022 Sharma [93]	Low	Low	Low	Low	Low	Low
2022 Sohl [94]	Low	Low	Low	Low	Low	Low
2022 Subramanian [95]	Low	Low	Low	Low	Low	Low
2023 Bower [96]	Low	Low	Low	Low	Low	Low
2023 Chauhan [97]	Low	Low	Low	Low	Low	Low
2023 Dua [98]	Low	Low	Low	Low	Low	Low
2023 Gautam [99]	Low	Low	Low	Low	Low	Low
2023 Jain [100]	Low	Low	Low	Low	Low	Low
2023 Lindsay [101]	Low	Low	Low	Low	Low	Low
2023 Liu [102]	Low	Low	Low	SC	Low	SC
2023 Mullapudi [103]	Low	Low	Low	SC	Low	SC
2023 Singh [104]	Low	Low	Low	Low	Low	Low
2023 Xu [105]	Low	Low	Low	Low	Low	Low

Low: low risk of bias; SC: some concerns; High: high risk of bias.

**Table 3:** Meta-analysis of MBI post-intervention effect on biomarkers.

		N	MBI			Control				MBI/Control Difference
			SMD (95% CI)	I^2^	*p*	SMD (95% CI)		N	SMD (95% CI)	SMD (95% CI)
CRP		47	−0.12 (−0.23 to −0.01)	44.0%	<0.01	−0.02 (−0.11 to 0.06)				−0.13 (−0.21 to −0.05)
	Static	31	−0.10 (−0.23 to 0.04)	47.7%		−0.02 (−0.13 to 0.09)	AC	21	−3.73 (−11.68 to 4.22)	−0.11 (−0.19 to −0.02)
	Qigong	3	−0.17 (−0.99 to 0.65)	37.6%		−0.01 (−0.42 to 0.40)	WL	5	−0.01 (−0.08 to 0.06)	−0.11 (−0.49 to 0.26)
	Yoga	9	−0.24 (−0.56 to 0.07)	37.6%		−0.05 (−0.25 to 0.15)	TAU	18	−0.05 (−0.20 to 0.09)	−0.25 (−0.55 to 0.03)
	Cancer	8	−0.14 (−0.41 to 0.13)	22.6%		−0.01 (−0.11 to 0.10)				−0.19 (−0.36 to −0.02)
	CNS	22	−0.16 (−0.34 to 0.02)	58.6%		−0.02 (−0.14 to 0.11)				−0.06 (−0.13 to 0.01)
	MD	13	−0.06 (−0.26 to 0.14)	20.0%		−0.06 (−0.28 to 0.17)				−0.23 (−0.46 to 0.01)
IL-6		57	−0.24 (−0.57 to 0.08)	93.0%	<0.01	−0.17 (−0.51 to 0.17)				−0.11 (−0.30 to 0.08)
	Static	29	−0.13 (−0.29 to 0.02)	54.2%		0.20 (−0.08 to 0.12)	AC	28	−0.26 (−0.92 to 0.39)	−0.14 (−0.27 to −0.02)
	Qigong	10	−0.09 (−0.37 to 0.20)	45.9%		0.04 (−0.13 to 0.21)	WL	8	0.02 (−0.12 to 0.16)	−0.11 (−0.42 to 0.19)
	Yoga	14	−0.59 (−1.90 to 0.71)	97.9%		−0.72 (−2.09 to 0.65)	TAU	17	0.02 (−0.12 to 0.16)	−0.05 (−0.73 to 0.63)
	CNS	25	−0.19 (−0.34 to −0.03)	59.0%	<0.01	0.03 (−0.08 to 0.15)				−0.15 (−0.28 to −0.03)
	MD	15	−0.71 (−1.81 to −0.04)	97.2%		−0.50 (−1.71 to 0.71)				−0.27 (−0.61 to 0.08)
	Cancer	13	0.24 (−0.29 to 0.76)	89.1%	<0.01	−0.19 (−0.72 to 0.34)				0.25 (−0.46 to 0.95)
IL-6-Cancer	Static	5	−0.12 (−0.27 to 0.02)	0%		−0.01 (−0.23 to 0.23)	AC	6	0.07 (−0.30 to 0.44)	−0.11 (−0.23 to 0.01)
	Qigong	3	0.29 (0.10 to 0.47)	0%		−0.12 (−0.58 to 0.33)	WL	3	−0.06 (−0.72 to 0.60)	0.33 (0.29 to 0.36)
	Yoga	5	0.61 (−1.14 to 2.36)	96.0%		−0.44 (−2.31 to 1.43)	TAU	4	−0.64 (−3.08 to 1.80)	0.64 (−1.84 to 3.13)
IL-6-CNS	Static	18	−0.16 (−0.38 to 0.05)	64.6%		−0.01 (−0.11 to 0.10)	AC	18	0.03 (−0.10 to 0.16)	−0.12 (−0.28 to 0.05)
	Qigong	5	−0.27 (−0.56 to 0.02)	19.7%		0.04 (−0.11 to 0.20)	WL	4	0.05 (−0.11 to 0.20)	−0.21 (−0.51 to 0.10)
	Yoga	2	−0.21 (−3.15 to 2.72)	69.0%		0.17 (−2.56 to 2.91)	TAU	3	−0.13 (−0.48 to 0.22)	−0.29 (−4.53 to 3.95)
TNF-α		26	−0.37 (−0.73 to −0.01)	94.0%	<0.01	−0.02 (−0.14 to 0.10)				−0.24 (−0.42 to −0.06)
	Static	11	−0.09 (−0.34 to 0.17)	34.6%		0.13 (−0.04 to 0.30)	AC	14	−0.06 (−0.25 to 0.13)	−0.15 (−0.34 to 0.03)
	Qigong	5	−0.01 (−0.24 to 0.22)	0%		0.06 (−0.11 to 0.23)	WL	3	−0.03 (−0.67 to 0.62)	−0.04 (−0.15 to 0.07)
	Yoga	9	−0.92 (−1.88 to 0.03)	97.1%		−0.22 (−0.45 to 0.02)	TAU	8	0.05 (−0.17 to 0.27)	−0.46 (−0.95 to 0.03)
	Cancer	5	−0.66 (−2.42 to 1.11)	97.2%		NA				−0.18 (−0.46 to 0.11)
	CNS	13	−0.10 (−0.34 to 0.13)	38.3%		NA				−0.12 (−0.27 to 0.03)
	MD	7	−0.66 (−1.61 to 0.30)	96.7%		NA				−0.45 (−1.01 to 0.12)
BDNF		11	0.30 (−0.25 to 0.85)	84.0%	<0.01	0.15 (−0.22 to 0.51)				0.29 (−0.19 to 0.78)
	Static	5	−0.18 (−1.26 to 0.91)	73.0%		0.24 (−0.79 to 1.27)	AC	4	0.59 (−0.31 to 1.48)	−0.14 (−0.92 to 0.65)
	Qigong	3	0.70 (−0.62 to 2.02)	78.8 %		0.12 (−0.28 to 0.51)	TAU	7	−0.07 (−0.44 to 0.30)	0.68 (−1.03 to 2.38)
	Yoga	3	0.73 (−0.83 to 2.29)	73.6%		0.03 (−0.28 to 0.34)				0.65 (−0.93 to 2.24)
IL-1		10	−0.40 (−0.72 to −0.08)	61.0%	<0.01	0.39 (−0.45 to 1.23)				−0.24 (−0.37 to −0.11)
	Static	3	−0.15 (−0.59 to 0.29)	43.7%		0.05 (−0.14 to 0.24)	AC	2	0.04 (−6.46 to 6.53)	−0.14 (−0.83 to 0.55)
	Qigong	3	−0.49 (−2.32 to 1.34)	0%		0.09 (−1.20 to 1.37)	WL	3	1.09 (−3.46 to 5.63)	−0.32 (−0.58 to −0.06)
	Yoga	3	−0.57 (−1.05 to −0.09)	0%		1.02 (−3.67 to 5.71)	TAU	4	0.04 (−0.27 to 0.35)	−0.24 (−0.53 to 0.06)
IL-8		13	−0.24 (−0.56 to 0.08)	72.0%	<0.01	0.07 (−0.23 to 0.38)				−0.13 (−0.29 to 0.03)
	Static	9	−0.23 (−0.63 to 0.18)	72.9%		−0.01 (−0.22 to 0.21)	AC	7	−0.12 (−0.38 to 0.15)	−0.14 (−0.37 to 0.09)
	Qigong	3	0.03 (−0.16 to 0.23)	0%		0.69 (−10.66 to 12.05)	TAU	5	0.32 (−0.42 to 1.06)	−0.09 (−0.79 to 0.61)
	Yoga	1	−0.80 (−1.71 to 0.11)	NA		−0.09 (−0.78 to 0.60)				−0.19 (−0.68 to 0.29)
IL-17		4	−0.30 (−0.88 to 0.28)	50.0%	0.11	0.52 (−0.13 to 1.16)				−0.75 (−1.74 to 0.23)
	Static	1	0.05 (−0.46 to 0.56)	NA		0.09 (−0.40 to 0.58)				−0.02 (−1.12 to 1.09)
	Qigong	1	−0.02 (−0.51 to 0.47)	NA		1.07 (0.55 to 1.60)				−0.76 (−1.87 to 0.35)
	Yoga	2	−0.61 (−0.92 to −0.31)	76.9%		0.46 (0.41 to 0.51)				−1.13 (−6.28 to 4.02)
IFN-γ		9	0.32 (−0.10 to 0.74)	86.1%	0.12	−0.17 (−0.54 to 0.20)	AC	3	0.05 (−0.21 to 0.31)	0.46 (−0.06 to 0.99)
	Static	6	0.41 (−0.26 to 1.08)	90.8%		0.01 (−0.08 to 0.08)	WL	5	−0.60 (−1.24 to 0.04)	0.51 (−0.28 to 1.29)
	Qigong	3	0.12 (−0.48 to 0.72)	0%		−0.47 (−1.89 to 0.95)	TAU	1	−0.14 (−1.04 to 0.76)	0.37 (−1.25 to 1.98)
IL-10		11	0.38 (−0.21 to 0.97)	89.0%	<0.01	−0.02 (−0.15 to 0.12)				0.27 (−0.06 to 0.61)
	Static	6	0.27 (−0.46 to 1.01)	75.7%		0.07 (−0.11 to 0.26)	AC	5	0.16 (−0.01 to 0.32)	0.19 (−0.34 to 0.74)
	Qigong	3	−0.10 (−0.48 to 0.28)	0%		−0.11 (−0.71 to 0.50)	TAU	6	−0.15 (−0.28 to −0.03)	0.04 (−0.15 to 0.22)
	Yoga	2	1.51 (−11.57 to 14.59)	57.5%		−0.17 (−1.15 to 0.82)				0.91 (−3.29 to 5.11)
	Cancer	1	−0.01 (−1.71 to 1.68)	NA		0.15 (−0.42 to 0.71)				−0.05 (−1.09 to 0.98)
	CNS	8	0.23 (−0.30 to 0.75)	83.7%		−0.02 (−0.21 to 0.17)				0.23 (−0.15 to 0.60)
	MD	2	1.22 (−15.21 to 17.64)	97.2%		−0.08 (−0.44 to 0.28)				0.61 (−7.11 to 8.32)
IL1-ra		4	0.02 (−0.15 to 0.19)	0%	0.76	0.02 (−0.18 to 0.22)				−0.02 (−0.23 to 0.19)
ESR		3	−0.16 (−0.71 to 0.38)	0%	0.77	−0.47 (−7.63 to 6.68)				−0.01 (−0.71 to 0.69)
NF-κB		2	−0.57 (−8.07 to 6.92)	89.0%	<0.01	−0.78 (−6.40 to 4.84)				0.09 (−2.59 to 2.77)
Cortisol		22	−0.33 (−0.55 to −0.12)	75.3%	<0.01	−0.18 (−0.34 to −0.02)				−0.16 (−0.34 to 0.03)
	Static	16	−0.35 (−0.62 to −0.09)	78.0%		−0.22 (−0.44 to 0.01)	AC	9	−0.15 (−0.40 to 0.09)	−0.12 (−0.29 to 0.04)
	Qigong	4	−0.12 (−0.91 to 0.67)	57.5%		−0.02 (−0.39 to 0.34)	WL	1	−0.01 (−0.01 to −0.01)	−0.33 (−1.87 to 1.21)
	Yoga	2	−0.54 (−3.03 to 1.94)	0%		−0.22 (−1.14 to 0.70)	TAU	10	−0.23 (−0.51 to 0.05)	−0.24 (−1.22 to 0.75)
sIgA		3	0.59 (−0.21 to 1.40)	0%	0.45	0.13 (−0.25 to 0.52)				0.39 (−0.23 to 1.01)

NA: not available; N: study number; SMD: standardized mean difference; 95% CI: 95% confidence interval; CNS: central nervous system; MD: medical disease; AC: active control; WL: waitlist control; TAU: treatment as usual.

**Table 4:** Risk of bias across studies and subgroup meta-regression of MBI effect on biomarkers at post-intervention.

	Test for Heterogeneity				Subgroup Meta-Regression					
	Egger’s Test		Trim-and-Fill		Intervention Type			Medical Condition		
	t	*p*	I^2^	Added Study n	Q	*df*	*p*	Q	*df*	*p*
CRP	3.56	0.001	65.8%	16	0.98	2	0.614	0.59	2	0.745
IL-6	0.624	0.535	92.8%	0	0.71	2	0.701	4.34	2	0.114
IL-6-cancer	−1.375	0.197	89.1%	0	37.52	2	<0.001			
IL-6-CNS	−1.533	0.139	70.6%	3	0.57	2	0.752			
TNF-α	−1.077	0.293	94.0%	9	4.79	2	0.091	2.49	2	0.288
IL-10	0.712	0.494	88.8%	0	3.86	2	0.145	0.66	2	0.719
IFN-γ	−0.025	0.981	84.0%	2	0.95	1	0.329			
BDNF	−0.529	0.610	83.9%	0	3.81	2	0.149			
Cortisol	−0.647	0.525	75.3%	0	1.84	2	0.399			

CRP: C-reactive protein; IL-6: interleukin-6; TNF-α: tumor necrosis factor-α; IL-10: interleukin-10; IFN-γ: interferon-γ; BDNF: brain-derived neurotrophic factor; *df*: degrees of freedom.

## Data Availability

Not applicable.

## Supplementary Materials

The following supporting information can be downloaded at: https://www.mdpi.com/article/10.3390/healthcare13080952/s1, Figure S1. Forest plot of the standardized mean difference of mind-body interventions effect on biomarkers. (A) CRP, (B) IL-6, (C) IL-6-CNS, (D) TNF-α, (E) IL-1, (F) IL-8, (G) IL-17, (H) IL-1ra, (I) ESR, (J) Cortisol, (K) sIgA. Control: pre-MBI effect. Experimental: post-MBI effect. Figure S2. Forest plot of the standardized mean difference of control interventions effect on biomarkers. (A) CRP, (B) IL-6, (C) BDNF, (D) TNF-α, (E) IL-1, (F) IL-8, (G) IL-17, (H) IL-1ra, (I) ESR, (J) Cortisol, (K) sIgA. Control: pre-intervention effect. Experimental: post-intervention effect. Figure S3. Forest plot of the standardized mean difference of postintervention effect between mind-body interventions and controls on biomarkers. (A) CRP, (B) IL-6, (C) BDNF, (D) TNF-α, (E) IL-1, (F) IL-8, (G) IL-17, (H) IL-1ra, (I) ESR, (J) Cortisol, (K) sIgA. Control: Control group. Experimental: MBI group. Figure S4. Funnel plot for publication bias of the standardized mean difference of mind-body interventions effect on biomarkers. (A) CRP, (B) IL-6, (C) TNF-α, (D) IL-1, (E) IL-8, (F) Cortisol. Figure S5. Funnel plot of the Trim-and-fill analysis of mind-body interventions effect on biomarkers. (A) CRP, (B) IL-6, (C) IL-6-CNS, (D) IL-6-cancer, (E) TNF-α, (F) IL-10, (G) IFN-γ, (H) BDNF, (I) Cortisol. Table S1. Statistical information between groups for each biomarker.

## Abbreviations

BDNF, brain-derived neurotrophic factor; CNS, central nervous system; CRP, C-reactive protein; HPA, hypothalamic–pituitary–adrenal; MBI, mindfulness-based intervention; MCI, mild cognitive impairment; RCT, randomized controlled trial; SMD, standardized mean difference; TNF-α, tumor necrosis factor-alpha.
