# Supplementary material for: Effects of Mind–Body Interventions on Immune and Neuroendocrine Functions: A Systematic Review and Meta-Analysis of Randomized Controlled Trials
Source: Healthcare (Basel). 2025 Apr 21;13(8):952. doi: 10.3390/healthcare13080952 (PMC12027091; doi:10.3390/healthcare13080952)
Supplement: Supplementary file 1 [file healthcare-13-00952-s001.zip › healthcare-3485063-supplementary.pdf]

Supplementary Fig. S1 Forest plot of the standardized mean difference of mind-body interventions effect on biomarkers. (A) CRP, (B) IL-6, (C) IL-6-CNS, (D) TNF-α, (E) IL-1, (F) IL-8, (G) IL-17, (H) IL-1ra, (I) ESR, (J) Cortisol, (K) sIgA.

Control: pre-MBI effect. Experimental: post-MBI effect.

(A) CRP

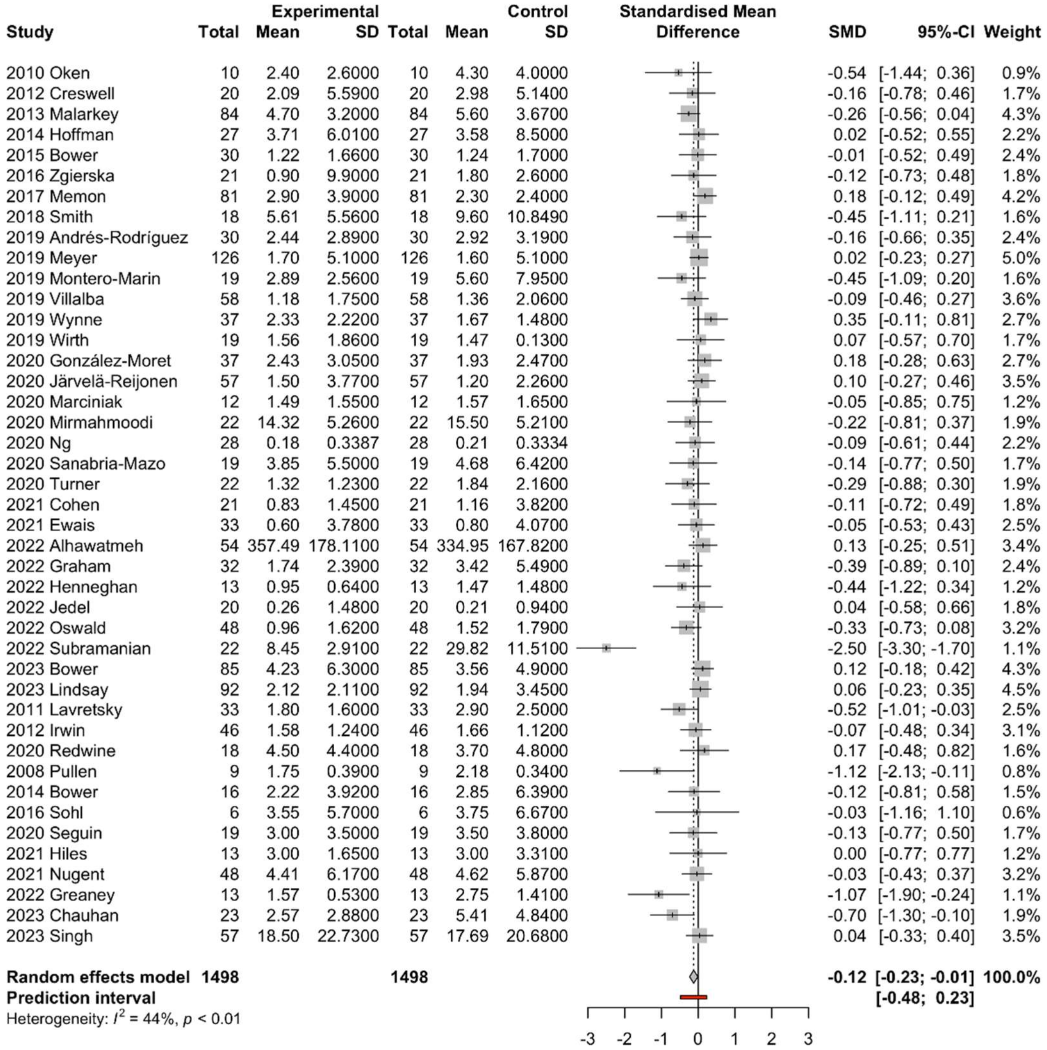

(B) IL-6

Study	Experimental Total	Experimental Mean	Experimental SD	Control Total	Control Mean	Control SD	SMD	95%-CI	Weight
2010 Oken	10	2.00	1.2000	10	2.20	1.2000	-0.16	[-1.04; 0.72]	1.7%
2014 Hoffman	27	1.99	0.0900	27	2.00	0.0600	-0.13	[-0.66; 0.41]	1.9%
2015 Bower	30	1.19	0.6700	30	1.24	0.8700	-0.06	[-0.57; 0.44]	1.9%
2016 Creswell	18	1.45	1.3600	18	1.87	1.3200	-0.31	[-0.96; 0.35]	1.8%
2016 Zgierska	21	2.20	7.0100	21	3.00	3.5000	-0.14	[-0.75; 0.46]	1.9%
2017 Sarenmalm	62	1.00	0.8100	62	1.00	1.1900	0.00	[-0.35; 0.35]	2.0%
2018 Hoge	42	120.00	114.0000	42	157.00	145.0000	-0.28	[-0.71; 0.15]	1.9%
2018 Smith	18	2.45	0.9200	18	2.93	1.6550	-0.35	[-1.01; 0.31]	1.8%
2019 Andrés-Rodríguez	30	4.16	4.3600	30	4.09	4.2200	0.02	[-0.49; 0.52]	1.9%
2019 Janusek	63	1.43	0.9100	63	1.81	1.6100	-0.29	[-0.64; 0.06]	2.0%
2019 Meyer	126	1.60	3.9700	126	1.60	3.4000	0.00	[-0.25; 0.25]	2.0%
2019 Montero-Marin	19	2.39	0.7000	19	2.90	1.5600	-0.41	[-1.06; 0.23]	1.9%
2020 Marciniak	12	2.00	1.3100	12	1.57	0.6500	0.40	[-0.41; 1.21]	1.8%
2020 Ng	28	0.46	0.5715	28	0.34	0.5715	0.20	[-0.33; 0.72]	1.9%
2020 Sanabria-Mazo	19	3.44	1.1200	19	3.35	2.6300	0.04	[-0.59; 0.68]	1.9%
2021 Cohen	21	0.46	0.3200	21	0.47	0.4800	-0.02	[-0.63; 0.58]	1.9%
2021 Ewais	33	2.23	2.2800	33	1.02	0.4800	0.73	[0.23; 1.22]	1.9%
2021 Oliveira	21	2.74	0.5800	21	3.87	0.9300	-1.43	[-2.12; -0.75]	1.8%
2022 Diez	30	18.82	28.3800	30	18.54	29.1400	0.01	[-0.50; 0.52]	1.9%
2022 Gardi	46	2.12	3.0400	46	3.06	3.4100	-0.29	[-0.70; 0.12]	1.9%
2022 Graham	32	5.10	7.4900	32	4.40	6.1000	0.10	[-0.39; 0.59]	1.9%
2022 Jedel	20	2.09	1.8300	20	1.97	1.2100	0.08	[-0.54; 0.70]	1.9%
2022 Lindsay	89	92.84	41.4200	89	78.18	37.3600	0.37	[0.07; 0.67]	2.0%
2022 Ng	93	1.05	0.6100	93	1.24	0.7200	-0.28	[-0.57; 0.01]	2.0%
2022 Oswald	48	1.55	1.6900	48	1.88	1.6200	-0.20	[-0.60; 0.20]	1.9%
2022 Saban	39	8.89	1.0000	39	9.17	1.0100	-0.28	[-0.72; 0.17]	1.9%
2023 Bower	85	0.94	0.9000	85	1.04	1.8000	-0.07	[-0.37; 0.23]	2.0%
2023 Lindsay	92	2.86	2.8800	92	2.71	2.5900	0.05	[-0.23; 0.34]	2.0%
2023 Xu	26	49.18	6.2600	26	55.50	3.7300	-1.21	[-1.80; -0.61]	1.9%
2006 Chen	44	100.64	47.3500	44	138.66	78.6300	-0.58	[-1.01; -0.15]	1.9%
2011 Janelsins	9	4.63	6.9700	9	2.63	3.9600	0.34	[-0.60; 1.27]	1.7%
2012 Irwin	46	2.07	1.1400	46	2.25	1.4300	-0.14	[-0.55; 0.27]	1.9%
2012 Sprod	9	4.63	6.9600	9	2.63	3.9600	0.34	[-0.60; 1.27]	1.7%
2015 Campo	28	9.10	3.4800	28	6.80	14.8900	0.21	[-0.32; 0.74]	1.9%
2019 Cheung	136	0.12	1.1200	136	0.25	1.0600	-0.12	[-0.36; 0.12]	2.0%
2020 Redwine	18	2.00	1.4000	18	1.50	0.8000	0.43	[-0.23; 1.09]	1.8%
2021 Qi	22	-0.55	0.3600	22	-0.31	0.3100	-0.70	[-1.31; -0.09]	1.9%
2022 Huang	32	2.30	1.0600	32	2.45	1.5100	-0.11	[-0.60; 0.38]	1.9%
2022 Ng	95	1.04	0.5600	95	1.29	0.6600	-0.41	[-0.69; -0.12]	2.0%
2008 Pullen	9	13.60	4.5000	9	17.50	5.9000	-0.71	[-1.67; 0.25]	1.7%
2013 Malarkey	84	1.91	16.0400	84	1.90	2.7500	0.00	[-0.30; 0.30]	2.0%
2014 Bower	16	1.19	0.8300	16	1.03	0.5400	0.22	[-0.47; 0.92]	1.8%
2014 Kiecolt-Glaser	92	9.69	0.4800	92	9.83	0.4500	-0.30	[-0.59; -0.01]	2.0%
2016 Sohl	6	3.21	6.0900	6	1.88	1.9000	0.27	[-0.87; 1.41]	1.6%
2020 Ganesan	68	60.42	62.8200	68	114.91	66.9800	-0.83	[-1.19; -0.48]	2.0%
2020 Gautam	31	2.40	1.7000	31	3.50	1.3000	-0.72	[-1.23; -0.20]	1.9%
2020 Viswanathan	150	13.30	1.2000	150	22.60	1.2000	-7.73	[-8.39; -7.07]	1.8%
2021 Nugent	48	1.95	1.6700	48	2.82	2.0400	-0.46	[-0.87; -0.06]	1.9%
2022 Chanta	13	17.92	9.5800	13	15.42	11.6600	0.23	[-0.54; 1.00]	1.8%
2022 Sohl	16	4.80	3.3800	16	5.60	4.1300	-0.21	[-0.90; 0.49]	1.8%
2022 Sharma	34	2.30	0.2600	34	2.40	0.2800	-0.37	[-0.85; 0.11]	1.9%
2023 Gautam	32	2.40	1.6000	32	3.40	1.2000	-0.70	[-1.20; -0.19]	1.9%
2023 Jain	48	11.09	0.9000	48	8.80	0.5700	3.02	[2.42; 3.61]	1.9%
**Random effects model**	**2286**			**2286**			**-0.24**	**[-0.57; 0.08]**	**100.0%**
**Prediction interval**								**[-2.57; 2.08]**	

Heterogeneity: $I^2 = 93\%$, $p < 0.01$

Standardised Mean Difference

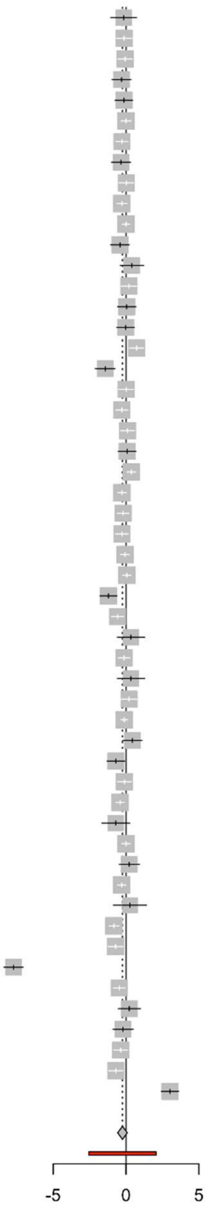

(C) IL-6-CNS

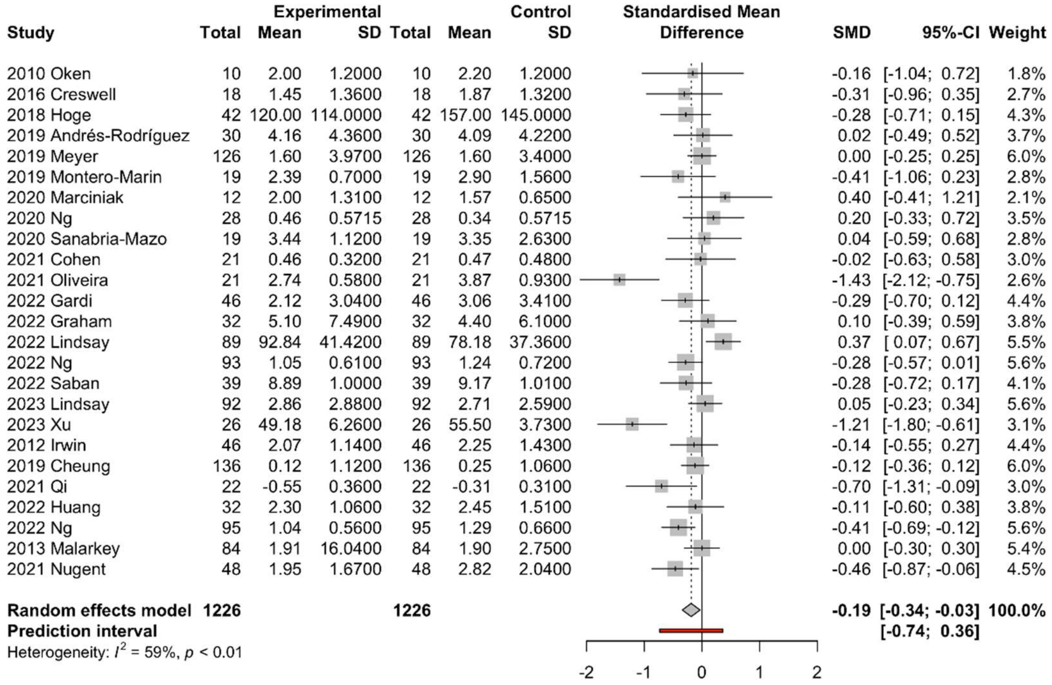

(D) TNF-α

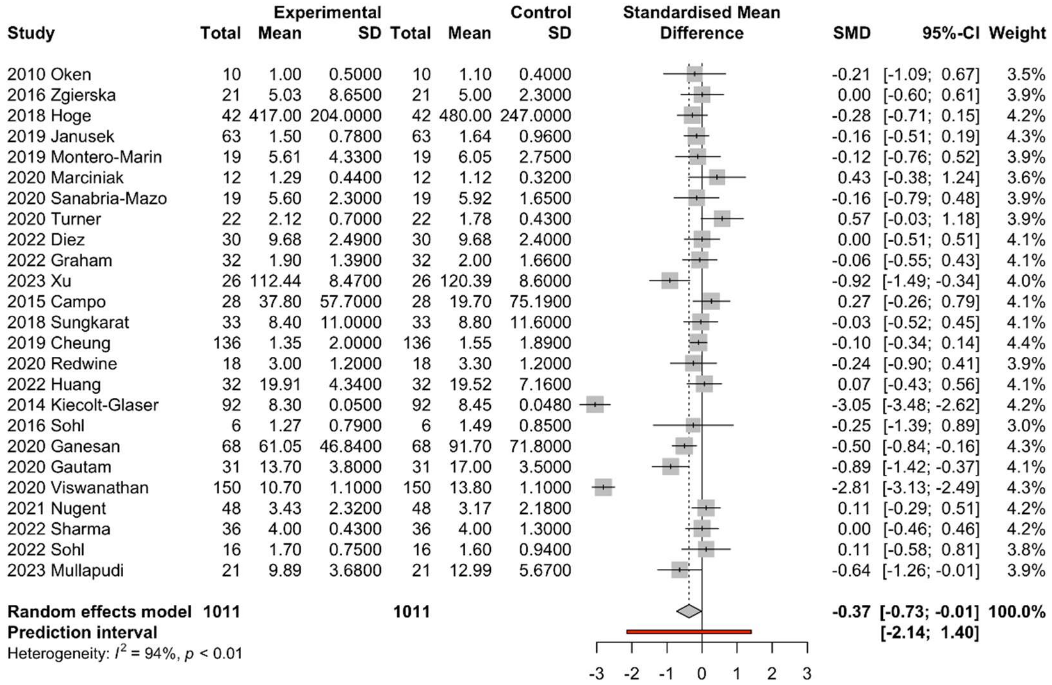

(E) IL-1

Study	Experimental Total	Experimental Mean	Experimental SD	Control Total	Control Mean	Control SD	SMD	95%-CI	Weight
2016 Zgierska	21	0.90	9.9000	21	0.60	1.5000	0.04	[-0.56; 0.65]	8.9%
2022 Diez	30	2.73	1.3900	30	2.88	1.3700	-0.11	[-0.61; 0.40]	10.5%
2022 Ng	93	0.07	0.1500	93	0.13	0.2300	-0.31	[-0.60; -0.02]	14.6%
2020 Ng	28	1.77	0.5503	28	1.65	0.5344	0.21	[-0.32; 0.73]	10.2%
2023 Xu	26	122.10	6.1200	26	128.44	3.0800	-1.29	[-1.89; -0.69]	9.0%
2022 Huang	32	2.31	1.0700	32	2.78	0.8500	-0.48	[-0.98; 0.02]	10.7%
2014 Kiecolt-Glaser	92	8.34	0.0860	92	8.39	0.0830	-0.59	[-0.88; -0.29]	14.5%
2015 Rajbhoj	19	0.55	0.1600	19	0.75	0.3000	-0.81	[-1.48; -0.15]	8.1%
2020 Ganesan	68	6.02	5.3100	68	11.97	20.4000	-0.40	[-0.74; -0.06]	13.6%
**Random effects model**	**409**			**409**			**-0.40**	**[-0.72; -0.08]**	**100.0%**
**Prediction interval**								**[-1.19; 0.38]**	

Heterogeneity: $I^2 = 61\%$, $p < 0.01$

Standardised Mean Difference

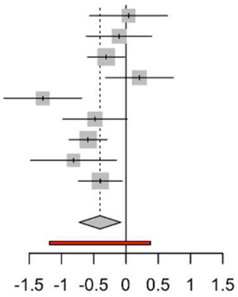

(F) IL-8

Study	Experimental Total	Experimental Mean	Experimental SD	Control Total	Control Mean	Control SD	SMD	95%-CI	Weight
2014 Hoffman	27	1.34	0.1800	27	1.34	0.1800	0.00	[-0.53; 0.53]	8.3%
2017 Memon	77	1.30	0.7000	77	1.30	0.6000	0.00	[-0.32; 0.32]	10.3%
2017 Sarenmalm	62	12.00	4.4400	62	11.50	3.1900	0.13	[-0.22; 0.48]	10.0%
2020 Turner	22	9.35	3.8600	22	8.43	3.2900	0.25	[-0.34; 0.85]	7.7%
2021 Oliveira	21	6.08	2.5000	21	8.17	3.8400	-0.63	[-1.25; -0.01]	7.5%
2022 Gardi	46	283.15	329.4200	46	459.55	437.2700	-0.45	[-0.87; -0.04]	9.4%
2022 Graham	32	4.30	3.8800	32	3.80	3.0500	0.14	[-0.35; 0.63]	8.7%
2022 Jedel	20	4.26	2.1000	20	5.03	4.7400	-0.21	[-0.83; 0.42]	7.5%
2023 Xu	26	121.03	5.8100	26	128.35	3.4100	-1.51	[-2.14; -0.89]	7.5%
2012 Sprod	9	9.69	6.1500	9	9.37	5.4000	0.05	[-0.87; 0.98]	5.2%
2022 Huang	32	10.35	9.1500	32	9.36	65.3500	0.02	[-0.47; 0.51]	8.7%
2023 Jain	48	17.53	0.5300	48	17.93	0.4600	-0.80	[-1.22; -0.38]	9.4%
**Random effects model**	**422**			**422**			**-0.24**	**[-0.56; 0.08]**	**100.0%**
**Prediction interval**								**[-1.22; 0.74]**	

Heterogeneity: $I^2 = 72\%$, $p < 0.01$

Standardised Mean Difference

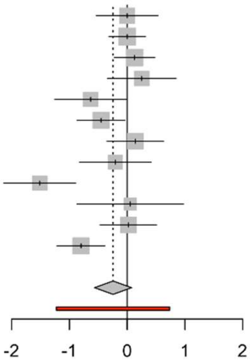

(G) IL-17

Study	Experimental Total	Experimental Mean	Experimental SD	Control Total	Control Mean	Control SD	SMD	95%-CI	Weight
2022 Diez	30	19.34	9.5000	30	18.89	8.3400	0.05	[-0.46; 0.56]	24.8%
2022 Huang	32	12.97	3.9300	32	13.06	4.7000	-0.02	[-0.51; 0.47]	25.6%
2020 Gautam	31	176.60	30.7000	31	195.00	31.0000	-0.59	[-1.10; -0.08]	24.6%
2023 Gautam	32	174.90	25.4000	32	191.40	25.8000	-0.64	[-1.14; -0.13]	25.0%
**Random effects model**	**125**			**125**			**-0.30**	**[-0.88; 0.28]**	**100.0%**
**Prediction interval**								**[-1.65; 1.06]**	

Heterogeneity: $I^2 = 50\%$, $p = 0.11$

Standardised Mean Difference

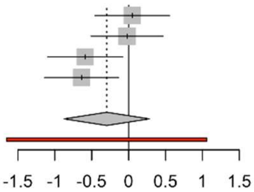

(H) IL-1ra

Study	Experimental Total	Experimental Mean	Experimental SD	Control Total	Control Mean	Control SD	SMD	95%-CI	Weight
2020 Järvelä-Reijonen	57	383.60	453.4000	57	356.50	368.2000	0.07	[-0.30; 0.43]	45.6%
2012 Irwin	46	238.00	144.0000	46	254.00	187.0000	-0.10	[-0.50; 0.31]	36.8%
2014 Bower	16	230.90	96.0000	16	220.90	95.0000	0.10	[-0.59; 0.80]	12.8%
2016 Sohl	6	334.18	249.6100	6	287.42	154.6700	0.21	[-0.93; 1.34]	4.8%
**Random effects model**	**125**			**125**			**0.02**	**[-0.15; 0.19]**	**100.0%**
**Prediction interval**								**[-0.53; 0.56]**	

Heterogeneity: $I^2 = 0\%$, $p = 0.91$

Standardised Mean Difference

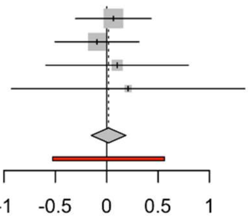

(I) ESR

Study	Experimental Total	Experimental Mean	Experimental SD	Control Total	Control Mean	Control SD	SMD	95%-CI	Weight
2021 Ewais	33	4.00	10.3700	33	6.00	7.4100	-0.22	[-0.70; 0.26]	36.6%
2023 Singh	57	34.45	25.8400	57	38.27	31.9900	-0.13	[-0.50; 0.24]	63.4%
**Random effects model**	**90**			**90**			**-0.16**	**[-0.71; 0.38]**	**100.0%**

Heterogeneity: $I^2 = 0\%$, $p = 0.77$

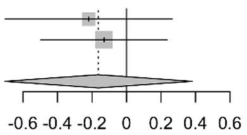

(J) Cortisol

Study	Experimental Total	Experimental Mean	Experimental SD	Control Total	Control Mean	Control SD	SMD	95%-CI	Weight
2008 McCain_R	65	0.44	1.6900	65	0.31	0.5900	0.10	[-0.24; 0.44]	5.5%
2008 McCain_S	68	0.22	1.6800	68	0.36	0.6200	-0.12	[-0.45; 0.22]	5.5%
2010 Oken	10	2.70	4.1000	10	4.00	4.6000	-0.29	[-1.17; 0.60]	3.0%
2013 Malarkey	84	0.11	24.3800	84	0.10	0.0900	0.00	[-0.30; 0.30]	5.7%
2018 Hoge	42	761.00	290.0000	42	913.00	252.0000	-0.55	[-0.99; -0.12]	5.0%
2018 Nery	62	0.40	0.2000	62	0.44	0.2500	-0.18	[-0.53; 0.18]	5.4%
2020 González-Moret	37	1.34	1.0700	37	1.75	1.1900	-0.36	[-0.82; 0.10]	4.9%
2020 Järvelä-Reijonen	57	109.70	39.2000	57	115.60	45.9000	-0.14	[-0.50; 0.23]	5.4%
2020 Mirmahmoodi	22	18.27	4.2800	22	18.77	4.0500	-0.12	[-0.71; 0.47]	4.2%
2020 Ng	28	-1.00	0.2646	28	-1.01	0.3281	0.03	[-0.50; 0.55]	4.6%
2020 Turner	22	324.55	201.8200	22	313.65	123.4800	0.06	[-0.53; 0.66]	4.2%
2021 Cohen	21	0.05	1.1600	21	-0.22	0.8800	0.26	[-0.35; 0.86]	4.1%
2022 Alhawatmeh	54	430.90	169.8000	54	641.24	234.3900	-1.02	[-1.42; -0.62]	5.2%
2022 Gardi	46	2.06	0.6900	46	3.24	0.8200	-1.54	[-2.01; -1.08]	4.9%
2022 Saban	39	0.07	0.0400	39	0.10	0.0400	-0.74	[-1.20; -0.28]	4.9%
2022 Subramanian	22	295.11	241.1200	22	505.63	120.0400	-1.09	[-1.72; -0.45]	4.0%
2008 McCain	62	0.65	1.5700	62	0.35	0.6100	0.25	[-0.10; 0.60]	5.4%
2012 Sprod	9	23.50	11.3100	9	21.52	8.7300	0.19	[-0.74; 1.11]	2.8%
2013 Chan	18	3.30	4.6300	18	4.30	4.8300	-0.21	[-0.86; 0.45]	3.9%
2020 Lu	14	107.24	19.6300	14	126.31	23.3900	-0.86	[-1.64; -0.08]	3.4%
2009 Banasik	7	2.33	0.9900	7	2.52	0.1100	-0.25	[-1.31; 0.80]	2.4%
2020 Ganesan	68	88.80	61.8000	68	132.85	67.9200	-0.67	[-1.02; -0.33]	5.5%
**Random effects model**	**857**			**857**			**-0.33**	**[-0.55; -0.12]**	**100.0%**
**Prediction interval**								**[-1.22; 0.56]**	

Heterogeneity: $I^2 = 75\%$, $p < 0.01$

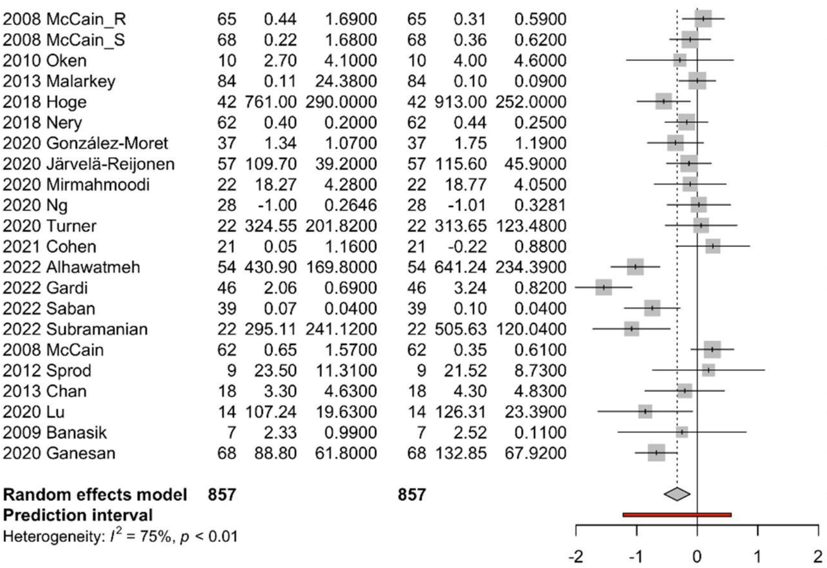

(K) sIgA

Study	Experimental Total	Experimental Mean	Experimental SD	Control Total	Control Mean	Control SD	SMD	95%-CI	Weight
2010 Fan	17	311.39	114.4800	17	262.16	88.2400	0.47	[-0.21; 1.15]	36.4%
2022 Martínez-Borrás	13	13.70	7.1400	13	7.44	3.8900	1.05	[0.23; 1.88]	24.7%
2013 Chan	18	72.90	73.0000	18	43.80	63.1400	0.42	[-0.24; 1.08]	38.8%
**Random effects model**	**48**			**48**			**0.59**	**[-0.21; 1.40]**	**100.0%**
**Prediction interval**								**[-2.08; 3.27]**	

Heterogeneity: $I^2 = 0\%$, $p = 0.45$

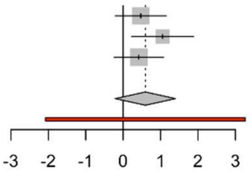

Supplementary Fig. S2 Forest plot of the standardized mean difference of control interventions effect on biomarkers. (A) CRP, (B) IL-6, (C) BDNF, (D) TNF-α, (E) IL-1, (F) IL-8, (G) IL-17, (H) IL-1ra, (I) ESR, (J) Cortisol, (K) sIgA.

Control: pre-intervention effect. Experimental: post-intervention effect.

(A) CRP

Study	Experimental Total	Experimental Mean	Experimental SD	Control Total	Control Mean	Control SD	SMD	95%-CI	Weight
2010 Oken	10	2.00	1.9000	10	3.10	4.1000	-0.33	[-1.21; 0.55]	0.7%
2012 Creswell	20	3.06	5.2300	20	3.42	5.2300	-0.07	[-0.69; 0.55]	1.4%
2013 Malarkey	85	5.67	3.6000	85	5.30	3.6900	0.10	[-0.20; 0.40]	6.1%
2014 Hoffman	28	5.45	7.8700	28	4.37	5.5700	0.16	[-0.37; 0.68]	2.0%
2015 Bower	22	1.32	1.7500	22	1.45	1.8100	-0.07	[-0.66; 0.52]	1.6%
2016 Zgierska	14	0.60	6.6600	14	0.50	2.6200	0.02	[-0.72; 0.76]	1.0%
2017 Memon	85	2.10	2.4000	85	1.90	1.9000	0.09	[-0.21; 0.39]	6.1%
2018 Smith	18	5.62	2.2600	18	5.74	5.1110	-0.03	[-0.68; 0.62]	1.3%
2019 Andrés-Rodríguez	27	2.27	2.3500	27	2.44	2.7200	-0.07	[-0.60; 0.47]	1.9%
2019 Meyer	130	1.60	5.1900	130	1.50	4.6100	0.02	[-0.22; 0.26]	9.3%
2019 Montero-Marin	15	4.07	4.1900	15	3.46	4.4200	0.14	[-0.58; 0.85]	1.1%
2019 Villalba	36	0.99	1.6800	36	1.05	1.6800	-0.04	[-0.50; 0.43]	2.6%
2019 Wynne	42	3.00	3.7000	42	2.67	2.9600	0.10	[-0.33; 0.53]	3.0%
2019 Wirth	17	1.86	0.4400	17	1.88	0.6400	-0.04	[-0.71; 0.64]	1.2%
2020 González-Moret	20	5.25	7.7300	20	2.46	3.8100	0.45	[-0.18; 1.08]	1.4%
2020 Järvelä-Reijonen	58	1.40	3.0400	58	1.50	3.8000	-0.03	[-0.39; 0.34]	4.2%
2020 Marciniak	8	1.15	1.6200	8	1.72	1.6600	-0.33	[-1.32; 0.66]	0.6%
2020 Mirmahmoodi	22	11.50	6.2800	22	12.09	6.2400	-0.09	[-0.68; 0.50]	1.6%
2020 Ng	27	0.20	0.3377	27	0.22	0.3274	-0.06	[-0.59; 0.47]	1.9%
2020 Sanabria-Mazo	15	4.00	4.1200	15	3.54	4.3600	0.11	[-0.61; 0.82]	1.1%
2020 Turner	25	1.78	2.2400	25	2.15	1.8900	-0.18	[-0.73; 0.38]	1.8%
2021 Cohen	17	0.99	1.3200	17	2.39	5.7500	-0.33	[-1.00; 0.35]	1.2%
2021 Ewais	31	0.30	0.5400	31	2.00	2.3700	-0.98	[-1.51; -0.45]	2.0%
2022 Alhawatmeh	54	415.30	195.5000	54	367.89	191.9600	0.24	[-0.14; 0.62]	3.8%
2022 Graham	29	4.67	7.8300	29	4.10	5.3400	0.08	[-0.43; 0.60]	2.1%
2022 Henneghan	12	1.16	1.1800	12	1.00	0.9700	0.14	[-0.66; 0.94]	0.9%
2022 Jedel	23	0.28	0.6200	23	0.45	1.7300	-0.13	[-0.71; 0.45]	1.6%
2022 Oswald	49	1.87	2.4000	49	1.75	2.3000	0.05	[-0.35; 0.45]	3.5%
2022 Subramanian	22	11.36	3.7900	22	25.00	9.5600	-1.84	[-2.56; -1.13]	1.1%
2023 Bower	81	3.92	5.0000	81	4.00	5.7000	-0.01	[-0.32; 0.29]	5.8%
2023 Lindsay	95	2.75	3.6100	95	2.54	3.4100	0.06	[-0.22; 0.34]	6.8%
2011 Lavretsky	35	2.20	2.1000	35	2.60	2.3000	-0.18	[-0.65; 0.29]	2.5%
2012 Irwin	37	1.75	1.8800	37	1.56	1.6900	0.11	[-0.35; 0.56]	2.6%
2020 Redwine	18	2.20	2.4000	18	2.00	3.0000	0.07	[-0.58; 0.73]	1.3%
2008 Pullen	10	1.90	0.4000	10	2.00	0.2300	-0.29	[-1.18; 0.59]	0.7%
2014 Bower	15	2.38	3.5900	15	1.52	0.2800	0.33	[-0.39; 1.05]	1.1%
2016 Sohl	5	5.60	23.3300	5	5.20	3.5600	0.02	[-1.22; 1.26]	0.4%
2020 Seguin	18	2.20	2.3000	18	2.80	3.8000	-0.19	[-0.84; 0.47]	1.3%
2021 Hiles	8	4.00	5.9800	8	1.00	10.7700	0.33	[-0.66; 1.31]	0.6%
2021 Nugent	39	3.70	4.9000	39	4.65	7.6900	-0.15	[-0.59; 0.30]	2.8%
2022 Greaney	12	2.52	1.8400	12	3.00	1.2600	-0.29	[-1.10; 0.51]	0.8%
2023 Chauhan	23	5.17	4.2300	23	3.59	3.5000	0.40	[-0.18; 0.98]	1.6%
2023 Singh	52	14.53	11.3100	52	16.87	11.8300	-0.20	[-0.59; 0.18]	3.7%
**Random effects model**	**1409**			**1409**			**-0.02**	**[-0.11; 0.06]**	**100.0%**
**Prediction interval**								**[-0.10; 0.05]**	

Heterogeneity: $I^2 = 21\%$, $p = 0.12$

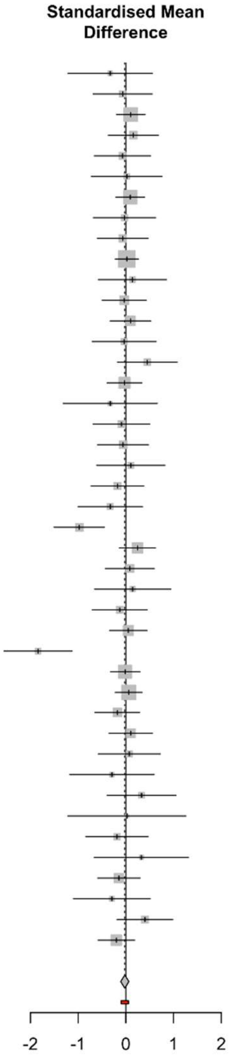

(B) IL-6

Study	Experimental Total	Experimental Mean	Experimental SD	Control Total	Control Mean	Control SD	SMD	95%-CI	Weight
2010 Oken	10	2.80	4.0000	10	1.50	1.3000	0.42	[-0.47; 1.31]	1.8%
2014 Hoffman	28	1.96	0.0800	28	1.98	0.0800	-0.25	[-0.77; 0.28]	1.9%
2015 Bower	22	1.32	0.6300	22	1.16	0.5800	0.26	[-0.33; 0.85]	1.9%
2016 Creswell	17	1.41	1.2400	17	1.17	1.3200	0.18	[-0.49; 0.86]	1.9%
2016 Zgierska	14	2.10	6.1700	14	1.80	1.3000	0.07	[-0.68; 0.81]	1.8%
2017 Sarenmalm	52	1.20	0.9600	52	1.10	1.4800	0.08	[-0.30; 0.46]	2.0%
2018 Hoge	28	96.00	70.0000	28	63.00	47.0000	0.55	[0.01; 1.08]	1.9%
2018 Smith	18	2.45	0.6750	18	2.15	1.2610	0.28	[-0.37; 0.94]	1.9%
2019 Andrés-Rodríguez	27	8.15	14.9800	27	7.88	12.3600	0.02	[-0.51; 0.55]	1.9%
2019 Janusek	61	1.68	1.0500	61	1.85	1.5000	-0.13	[-0.49; 0.22]	2.0%
2019 Meyer	131	1.50	2.8900	131	1.70	4.0500	-0.06	[-0.30; 0.19]	2.0%
2019 Montero-Marin	15	3.13	1.6000	15	3.18	1.1900	-0.03	[-0.75; 0.68]	1.8%
2020 Marciniak	8	2.48	2.8600	8	2.40	1.8200	0.03	[-0.95; 1.01]	1.7%
2020 Ng	27	0.41	0.5924	27	0.39	0.5352	0.03	[-0.51; 0.56]	1.9%
2020 Sanabria-Mazo	15	3.14	1.8700	15	3.04	1.1800	0.06	[-0.65; 0.78]	1.8%
2021 Cohen	17	0.66	0.6500	17	0.83	1.1900	-0.17	[-0.85; 0.50]	1.9%
2021 Ewais	31	2.46	1.8500	31	1.59	1.3100	0.54	[0.03; 1.04]	1.9%
2021 Oliveira	20	3.33	0.3700	20	3.25	0.5600	0.17	[-0.46; 0.79]	1.9%
2022 Diez	32	10.96	27.9900	32	11.24	29.5900	-0.01	[-0.50; 0.48]	1.9%
2022 Gardi	44	3.05	1.7000	44	3.16	1.9700	-0.06	[-0.48; 0.36]	1.9%
2022 Graham	29	3.10	3.9400	29	2.90	3.1500	0.06	[-0.46; 0.57]	1.9%
2022 Jedel	23	2.15	1.8200	23	2.54	2.7300	-0.17	[-0.74; 0.41]	1.9%
2022 Lindsay	93	82.84	39.8300	93	84.41	36.8400	-0.04	[-0.33; 0.25]	2.0%
2022 Ng	93	1.30	0.8600	93	1.19	0.6600	0.14	[-0.14; 0.43]	2.0%
2022 Oswald	49	1.79	1.7800	49	2.18	1.8500	-0.21	[-0.61; 0.18]	1.9%
2022 Saban	34	8.84	0.7400	34	8.99	1.1400	-0.15	[-0.63; 0.32]	1.9%
2023 Bower	81	0.81	0.6000	81	0.80	0.6000	0.02	[-0.29; 0.32]	2.0%
2023 Lindsay	95	3.50	2.8300	95	3.69	2.6300	-0.07	[-0.35; 0.22]	2.0%
2023 Xu	22	52.72	3.1300	22	55.54	2.8900	-0.92	[-1.54; -0.30]	1.9%
2006 Chen	43	140.56	56.2700	43	114.72	37.8100	0.53	[0.10; 0.96]	1.9%
2011 Janelsins	10	2.42	1.4700	10	2.44	1.7900	-0.01	[-0.89; 0.86]	1.8%
2012 Irwin	37	1.95	1.4300	37	1.87	1.0900	0.06	[-0.39; 0.52]	1.9%
2012 Sprod	10	2.42	1.7400	10	2.44	1.7700	-0.01	[-0.89; 0.87]	1.8%
2015 Campo	24	5.30	3.5600	24	6.80	5.4100	-0.32	[-0.89; 0.25]	1.9%
2019 Cheung	135	0.13	1.1200	135	0.12	1.0600	0.01	[-0.23; 0.25]	2.0%
2020 Redwine	18	1.30	0.7000	18	1.30	0.7000	0.00	[-0.65; 0.65]	1.9%
2021 Qi	26	-0.53	0.2200	26	-0.59	0.4000	0.18	[-0.36; 0.73]	1.9%
2022 Huang	32	1.86	1.0400	32	2.19	1.6200	-0.24	[-0.73; 0.25]	1.9%
2022 Ng	93	1.30	0.8600	93	1.19	0.6600	0.14	[-0.14; 0.43]	2.0%
2008 Pullen	10	18.10	2.0000	10	18.00	2.0000	0.05	[-0.83; 0.92]	1.8%
2013 Malarkey	85	1.76	1.0100	85	1.70	2.7700	0.03	[-0.27; 0.33]	2.0%
2014 Bower	15	1.64	1.2200	15	1.37	0.6900	0.27	[-0.45; 0.98]	1.8%
2014 Kiecolt-Glaser	80	9.83	0.0520	80	9.84	0.0470	-0.20	[-0.51; 0.11]	2.0%
2016 Sohl	5	4.59	4.6100	5	2.17	4.6700	0.47	[-0.80; 1.74]	1.6%
2020 Ganesan	75	79.35	84.2500	75	112.69	62.7200	-0.45	[-0.77; -0.12]	2.0%
2020 Gautam	31	3.70	1.5000	31	3.80	1.1000	-0.08	[-0.57; 0.42]	1.9%
2020 Viswanathan	150	12.30	1.2000	150	22.50	1.2000	-8.48	[-9.20; -7.76]	1.8%
2021 Nugent	39	3.79	3.9100	39	2.22	2.1000	0.50	[0.04; 0.95]	1.9%
2022 Chanta	14	12.92	7.9100	14	10.83	9.1700	0.24	[-0.51; 0.98]	1.8%
2022 Sohl	13	6.90	4.4700	13	4.60	2.9800	0.59	[-0.20; 1.37]	1.8%
2022 Sharma	31	2.30	0.2500	31	2.30	0.2700	0.00	[-0.50; 0.50]	1.9%
2023 Gautam	32	3.70	1.4000	32	3.70	1.1000	0.00	[-0.49; 0.49]	1.9%
2023 Jain	48	8.28	0.6400	48	9.96	0.4700	-2.97	[-3.55; -2.38]	1.9%
**Random effects model**	**2192**			**2192**			**-0.17**	**[-0.51; 0.17]**	**100.0%**
**Prediction interval**								**[-2.59; 2.25]**	

Heterogeneity: $I^2 = 92\%$, $p < 0.01$

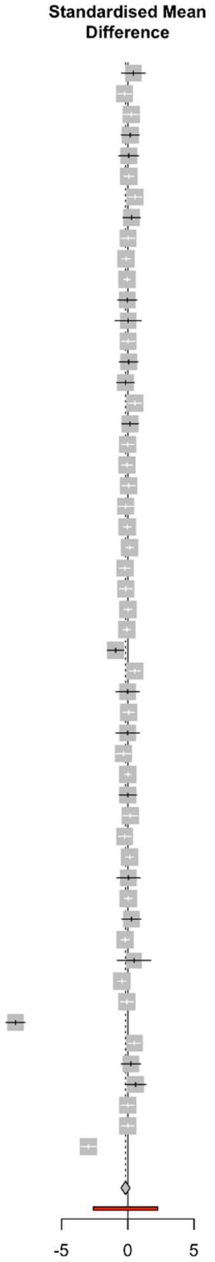

(C) BDNF

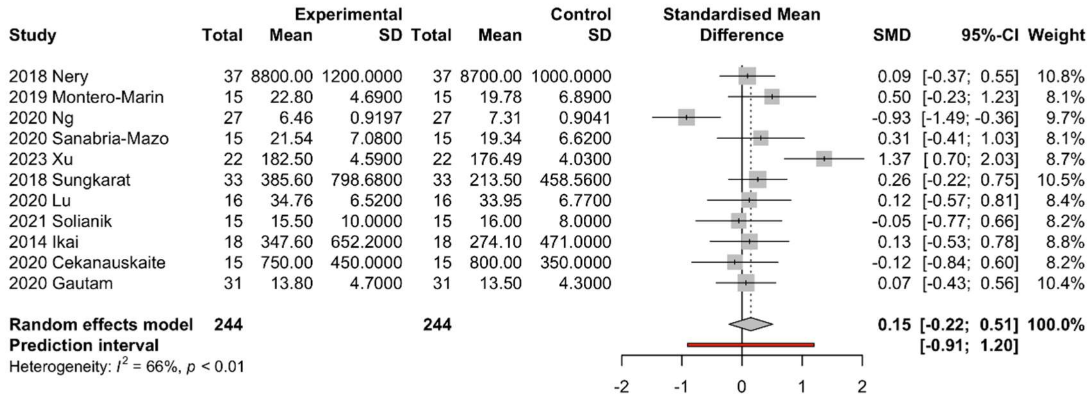

(D) TNF-α

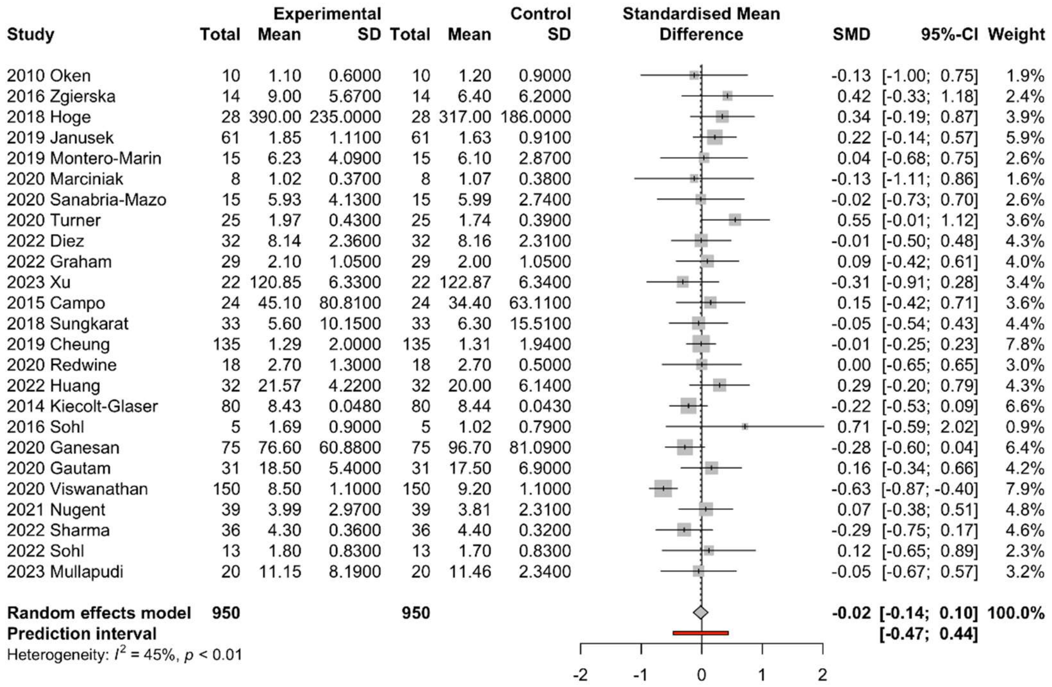

(E) IL-1

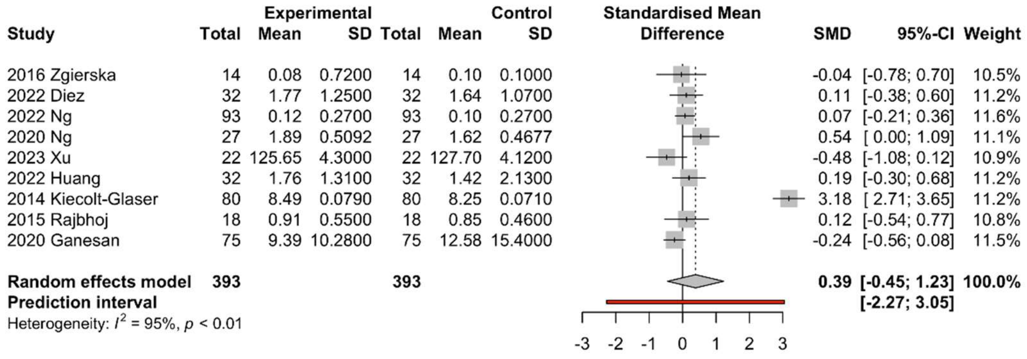

(F) IL-8

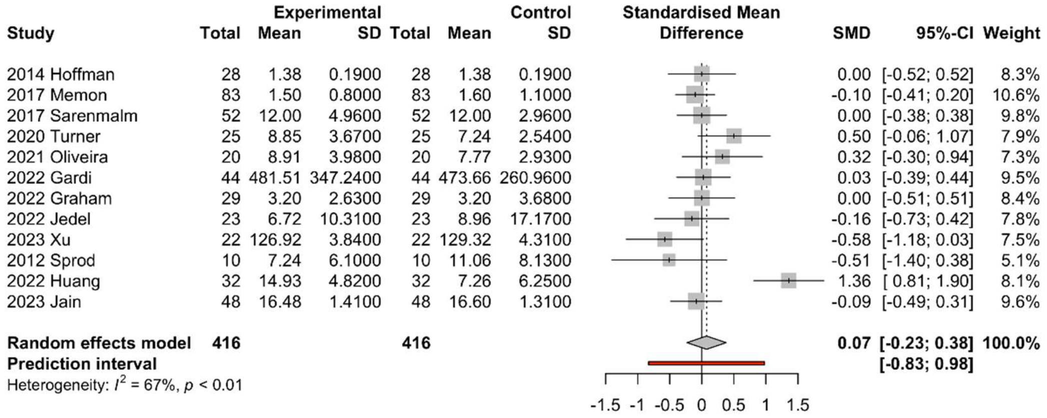

(G) IL-17

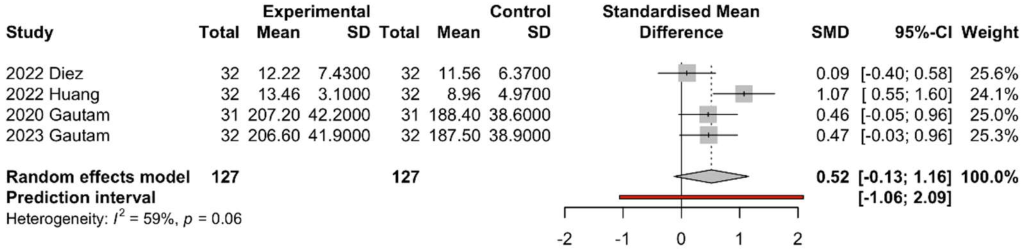

(H) IL-1ra

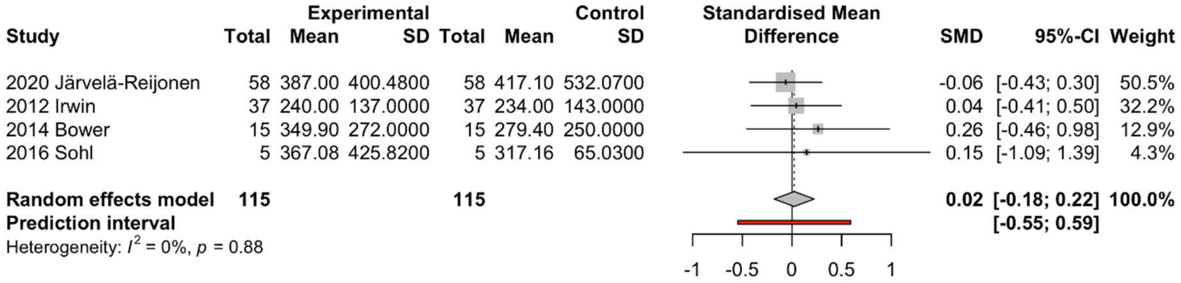

(I) ESR

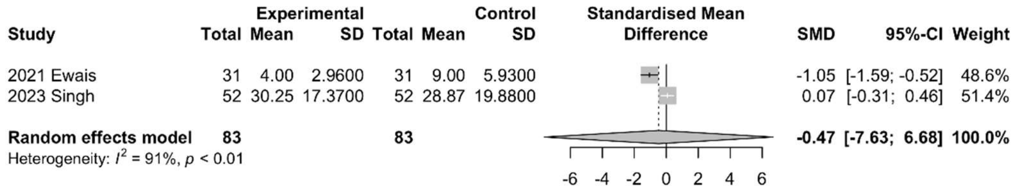

(J) Cortisol

Study	Experimental Total	Experimental Mean	Experimental SD	Control Total	Control Mean	Control SD	Standardised Mean Difference	SMD	95%-CI	Weight
2010 Oken	10	0.70	0.4000	10	1.20	0.9000		-0.69	[-1.60; 0.22]	2.3%
2013 Malarkey	85	0.12	0.0900	85	0.15	0.2800		-0.14	[-0.44; 0.16]	8.6%
2018 Hoge	28	951.00	430.0000	28	1040.00	383.0000		-0.22	[-0.74; 0.31]	5.0%
2018 Nery	37	0.63	0.0600	37	0.75	0.1300		-1.17	[-1.67; -0.68]	5.4%
2020 González-Moret	20	1.94	2.0100	20	2.18	1.9200		-0.12	[-0.74; 0.50]	4.0%
2020 Järvelä-Reijonen	58	116.10	41.4000	58	108.90	35.8000		0.18	[-0.18; 0.55]	7.4%
2020 Mirmahmoodi	22	16.68	3.9800	22	16.45	3.7800		0.06	[-0.53; 0.65]	4.3%
2020 Ng	27	-0.87	0.2858	27	-1.00	0.2598		0.44	[-0.10; 0.98]	4.9%
2020 Turner	25	289.32	168.9700	25	353.96	199.1500		-0.34	[-0.90; 0.21]	4.7%
2021 Cohen	17	-0.08	0.7600	17	0.27	1.1000		-0.36	[-1.04; 0.32]	3.6%
2022 Alhawatmeh	54	589.10	224.0000	54	629.35	236.6500		-0.17	[-0.55; 0.20]	7.1%
2022 Gardi	44	3.05	0.9000	44	3.27	1.0300		-0.23	[-0.64; 0.19]	6.5%
2022 Saban	34	0.11	0.0900	34	0.14	0.2900		-0.14	[-0.61; 0.34]	5.6%
2022 Subramanian	22	310.11	277.0700	22	444.75	175.5200		-0.57	[-1.17; 0.03]	4.2%
2008 McCain	57	0.27	1.7200	57	0.28	0.6100		-0.01	[-0.38; 0.36]	7.3%
2012 Sprod	10	28.69	7.9700	10	26.04	6.5800		0.35	[-0.54; 1.23]	2.4%
2013 Chan	16	4.30	4.5000	16	4.40	6.1900		-0.02	[-0.71; 0.67]	3.5%
2020 Lu	16	109.65	19.5800	16	116.07	19.2600		-0.32	[-1.02; 0.38]	3.4%
2009 Banasik	7	2.45	0.2900	7	2.56	0.2700		-0.37	[-1.43; 0.69]	1.7%
2020 Ganesan	75	119.70	70.3700	75	134.61	89.0200		-0.18	[-0.51; 0.14]	8.2%
**Random effects model**	**664**			**664**				**-0.18**	**[-0.34; -0.02]**	**100.0%**
**Prediction interval**									**[-0.66; 0.29]**	

Heterogeneity: I^2 = 39%, p = 0.04

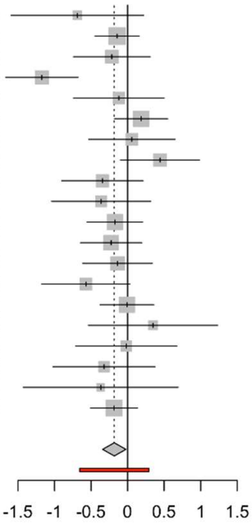

(K)sIgA

Study	Experimental Total	Experimental Mean	Experimental SD	Control Total	Control Mean	Control SD	Standardised Mean Difference	SMD	95%-CI	Weight
2010 Fan	18	245.52	79.1500	18	242.69	78.8300		0.04	[-0.62; 0.69]	40.2%
2022 Martínez-Borrás	11	8.30	3.2400	11	7.07	3.4600		0.35	[-0.49; 1.20]	24.1%
2013 Chan	16	67.40	88.9500	16	58.90	82.2000		0.10	[-0.60; 0.79]	35.7%
**Random effects model**	**45**			**45**				**0.13**	**[-0.25; 0.52]**	**100.0%**
**Prediction interval**									**[-2.55; 2.82]**	

Heterogeneity: I^2 = 0%, p = 0.84

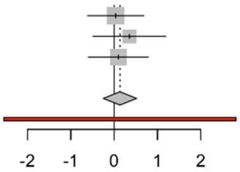

Supplementary Fig. S3 Forest plot of the standardized mean difference of post-intervention effect between mind-body interventions and controls on biomarkers. (A) CRP, (B) IL-6, (C) BDNF, (D) TNF-α, (E) IL-1, (F) IL-8, (G) IL-17, (H) IL-1ra, (I) ESR, (J) Cortisol, (K) sIgA.

Control: Control group. Experimental: MBI group.

(A) CRP

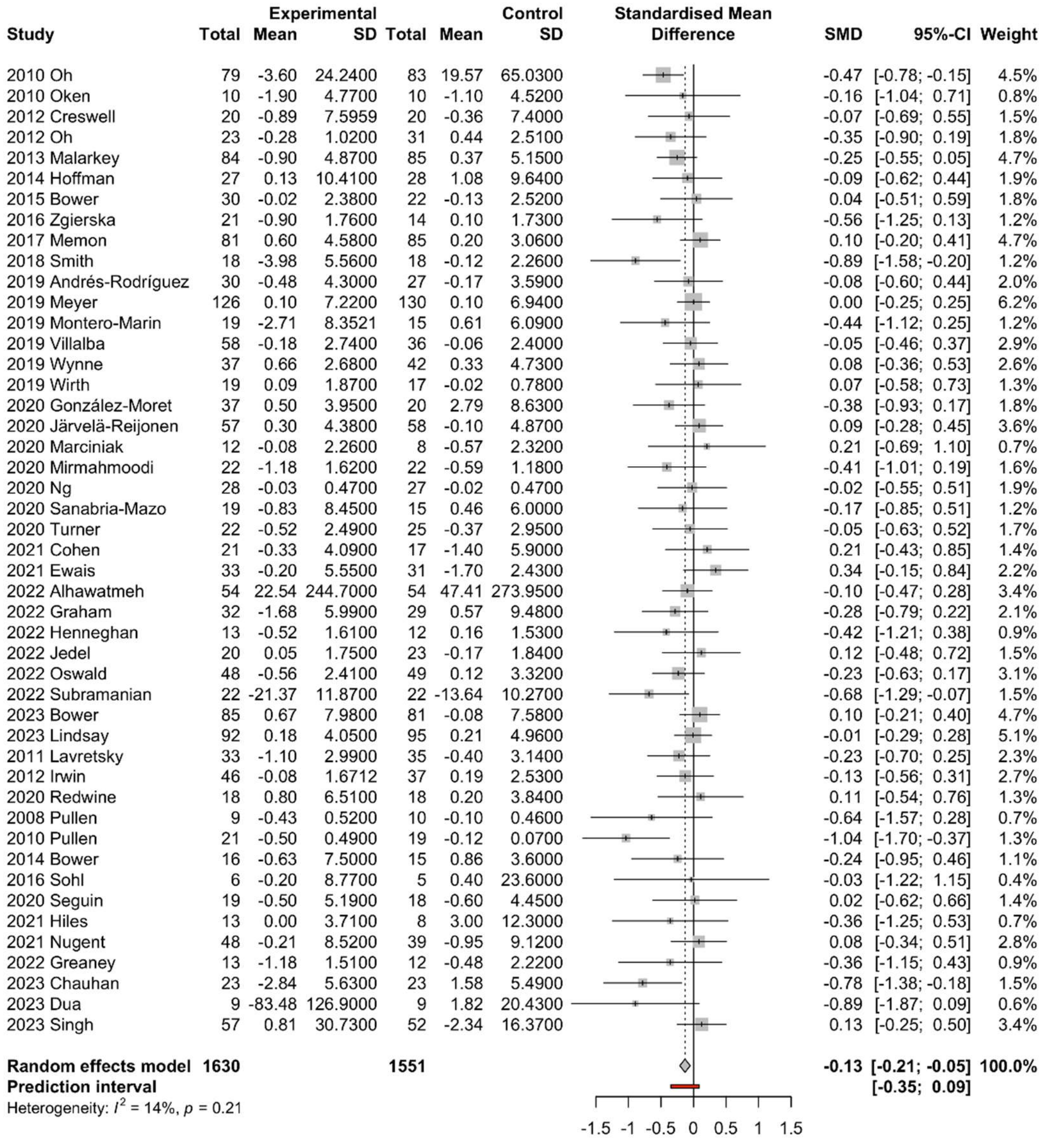

(B) IL-6

Study	Experimental Total	Experimental Mean	Experimental SD	Control Total	Control Mean	Control SD	SMD	95%-CI	Weight
2010 Oken	10	-0.20	1.7000	10	1.30	4.2000	-0.45	[-1.34; 0.44]	1.4%
2012 Creswell	20	0.14	4.4600	20	-0.38	4.4900	0.11	[-0.51; 0.73]	1.7%
2014 Hoffman	27	-0.01	0.1100	28	-0.02	0.1100	0.09	[-0.44; 0.62]	1.8%
2015 Bower	30	-0.05	1.1000	22	0.16	0.8600	-0.21	[-0.76; 0.35]	1.8%
2016 Creswell	18	-0.42	1.8900	17	0.24	1.8100	-0.35	[-1.02; 0.32]	1.6%
2016 Zgierska	21	-0.80	2.2000	14	0.30	1.9100	-0.51	[-1.20; 0.17]	1.6%
2017 Sarenmalm	52	-0.20	1.2800	52	0.10	1.7600	-0.19	[-0.58; 0.19]	1.9%
2018 Hoge	42	-37.00	156.0000	28	33.00	62.0000	-0.54	[-1.03; -0.06]	1.8%
2018 Smith	18	-0.48	0.9200	18	0.29	0.6700	-0.94	[-1.63; -0.25]	1.6%
2019 Andrés-Rodríguez	30	0.07	6.0700	27	0.27	19.4200	-0.01	[-0.53; 0.51]	1.8%
2019 Janusek	63	-0.38	1.8500	61	-0.17	1.8300	-0.11	[-0.47; 0.24]	2.0%
2019 Meyer	126	0.00	5.2300	131	-0.20	4.9800	0.04	[-0.21; 0.28]	2.1%
2019 Montero-Marin	19	-0.51	1.7100	15	-0.05	1.9900	-0.24	[-0.92; 0.44]	1.6%
2020 Marciniak	12	0.43	1.4600	8	0.08	3.3900	0.14	[-0.76; 1.04]	1.4%
2020 Ng	28	0.12	0.8100	27	0.01	0.7981	0.12	[-0.41; 0.65]	1.8%
2020 Sanabria-Mazo	19	0.09	2.8600	15	0.10	2.2100	-0.00	[-0.68; 0.67]	1.6%
2021 Cohen	21	-0.01	0.5800	17	-0.17	1.3600	0.16	[-0.48; 0.80]	1.7%
2021 Ewais	33	1.21	2.3300	31	0.87	2.2700	0.15	[-0.34; 0.64]	1.8%
2021 Oliveira	21	-1.13	1.1000	20	0.08	0.6700	-1.30	[-1.97; -0.62]	1.6%
2022 Diez	30	0.28	40.6800	32	-0.28	40.8100	0.01	[-0.48; 0.51]	1.8%
2022 Gardi	46	-0.94	4.5700	44	-0.11	2.6000	-0.22	[-0.63; 0.19]	1.9%
2022 Graham	32	0.70	9.6600	29	0.20	5.0500	0.06	[-0.44; 0.57]	1.8%
2022 Jedel	20	0.12	2.2000	23	-0.39	3.2900	0.18	[-0.42; 0.78]	1.7%
2022 Lindsay	89	14.66	55.7800	93	-1.57	54.2500	0.29	[0.00; 0.59]	2.0%
2022 Ng	93	-0.19	0.9400	93	0.11	1.0800	-0.30	[-0.58; -0.01]	2.0%
2022 Oswald	48	-0.33	2.3400	49	-0.39	2.5600	0.02	[-0.37; 0.42]	1.9%
2022 Saban	39	-0.28	1.4200	34	-0.15	1.3600	-0.09	[-0.55; 0.37]	1.9%
2023 Bower	85	-0.10	2.0100	81	0.01	0.8500	-0.07	[-0.37; 0.23]	2.0%
2023 Lindsay	92	0.15	3.8700	95	-0.19	3.8600	0.09	[-0.20; 0.37]	2.0%
2023 Xu	26	-6.32	7.2900	22	-2.82	4.2600	-0.56	[-1.14; 0.02]	1.7%
2006 Chen	44	-38.02	58.5500	43	25.83	56.0500	-1.10	[-1.56; -0.65]	1.9%
2011 Janelsins	9	2.00	8.0200	10	-0.02	2.3200	0.34	[-0.57; 1.24]	1.4%
2012 Irwin	46	-0.18	1.8285	37	0.08	1.8000	-0.14	[-0.58; 0.29]	1.9%
2012 Sprod	9	2.00	8.0100	10	-0.02	2.4800	0.33	[-0.57; 1.24]	1.4%
2015 Campo	28	2.30	15.2900	24	-1.50	6.4800	0.31	[-0.24; 0.86]	1.8%
2019 Cheung	136	-0.13	1.5400	135	0.01	1.5400	-0.09	[-0.33; 0.15]	2.1%
2020 Redwine	18	0.50	1.6100	18	0.00	0.9900	0.37	[-0.29; 1.03]	1.7%
2021 Qi	22	-0.24	0.4800	26	0.06	0.4600	-0.63	[-1.21; -0.05]	1.7%
2022 Huang	32	-0.15	1.8400	32	-0.33	1.9300	0.09	[-0.40; 0.58]	1.8%
2022 Ng	95	-0.25	0.8700	93	0.11	1.0800	-0.37	[-0.65; -0.08]	2.0%
2023 Liu	26	-0.39	1.9900	26	-0.29	1.9900	-0.05	[-0.59; 0.49]	1.8%
2008 Pullen	9	-3.90	7.4200	10	0.10	2.8300	-0.70	[-1.63; 0.24]	1.3%
2010 Pullen	21	-3.57	1.9700	19	0.59	1.5200	-2.30	[-3.12; -1.49]	1.5%
2013 Malarkey	84	0.01	16.2700	85	0.06	2.9500	-0.00	[-0.31; 0.30]	2.0%
2014 Bower	16	0.16	0.9900	15	0.27	1.4000	-0.09	[-0.79; 0.62]	1.6%
2014 Kiecolt-Glaser	92	-0.14	0.6600	80	-0.01	0.6300	-0.20	[-0.50; 0.10]	2.0%
2016 Sohl	6	1.33	6.3800	5	2.42	6.5600	-0.15	[-1.34; 1.04]	1.1%
2020 Ganesan	68	-54.49	91.8300	75	-33.34	105.0300	-0.21	[-0.54; 0.12]	2.0%
2020 Gautam	31	-1.00	1.1000	31	-0.10	1.4000	-0.71	[-1.22; -0.19]	1.8%
2020 Viswanathan	150	-9.30	1.7000	150	-10.20	1.7000	0.53	[0.30; 0.76]	2.1%
2021 Nugent	48	-0.87	2.6400	39	1.57	4.4400	-0.68	[-1.11; -0.24]	1.9%
2022 Chanta	13	2.50	15.0900	14	2.09	12.1100	0.03	[-0.73; 0.78]	1.5%
2022 Sohl	16	-0.80	5.3300	13	2.30	5.3700	-0.56	[-1.31; 0.18]	1.6%
2022 Sharma	34	-0.10	0.3800	31	0.00	0.3700	-0.26	[-0.75; 0.23]	1.8%
2023 Dua	9	-4.85	5.4100	9	-20.77	22.9600	0.91	[-0.07; 1.89]	1.3%
2023 Gautam	32	-1.00	2.0000	32	0.00	1.7800	-0.52	[-1.02; -0.02]	1.8%
2023 Jain	48	2.29	1.0700	48	-1.68	0.7900	4.19	[3.46; 4.91]	1.6%
**Random effects model**	**2352**			**2266**			**-0.11**	**[-0.30; 0.08]**	**100.0%**
**Prediction interval**								**[-1.37; 1.15]**	

Heterogeneity: $I^2 = 81\%$, $p < 0.01$

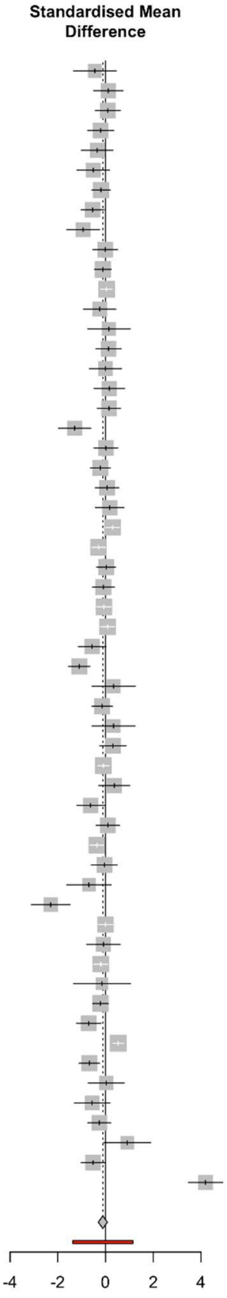

(C) BDNF

Study	Experimental Total	Experimental Mean	Experimental SD	Control Total	Control Mean	Control SD	SMD	95%-CI	Weight
2018 Nery	62	150.00	1345.3600	37	100.00	1562.0500	0.03	[-0.37; 0.44]	10.4%
2019 Montero-Marin	19	-6.69	8.3300	15	3.02	8.3347	-1.14	[-1.87; -0.40]	8.4%
2020 Ng	28	-0.74	1.2500	27	-0.86	1.2900	0.09	[-0.44; 0.62]	9.7%
2020 Sanabria-Mazo	19	-2.25	10.2700	15	2.20	9.6900	-0.43	[-1.12; 0.25]	8.7%
2023 Xu	26	14.46	18.0100	22	6.01	6.1100	0.60	[0.02; 1.18]	9.4%
2018 Sungkarat	33	152.10	892.8500	33	172.10	920.9600	-0.02	[-0.50; 0.46]	10.0%
2020 Lu	14	3.62	2.2800	16	0.80	2.2800	1.20	[0.42; 1.99]	8.1%
2021 Solianik	15	15.00	16.0100	15	-0.50	12.8100	1.04	[0.27; 1.81]	8.2%
2014 Ikai	18	41.60	377.0000	18	73.40	346.0000	-0.09	[-0.74; 0.57]	8.9%
2020 Cekanauskaite	18	607.00	631.3400	15	-50.00	570.0900	1.06	[0.32; 1.80]	8.4%
2020 Gautam	31	3.80	4.5000	31	0.20	2.4000	0.99	[0.46; 1.51]	9.7%
**Random effects model**	**283**			**244**			**0.29**	**[-0.19; 0.78]**	**100.0%**
**Prediction interval**								**[-1.20; 1.78]**	

Heterogeneity: $I^2 = 77\%$, $p < 0.01$

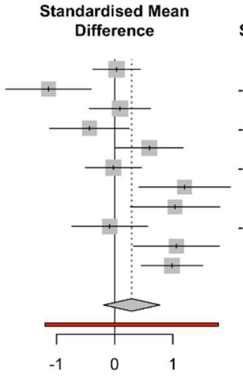

(D) TNF-α

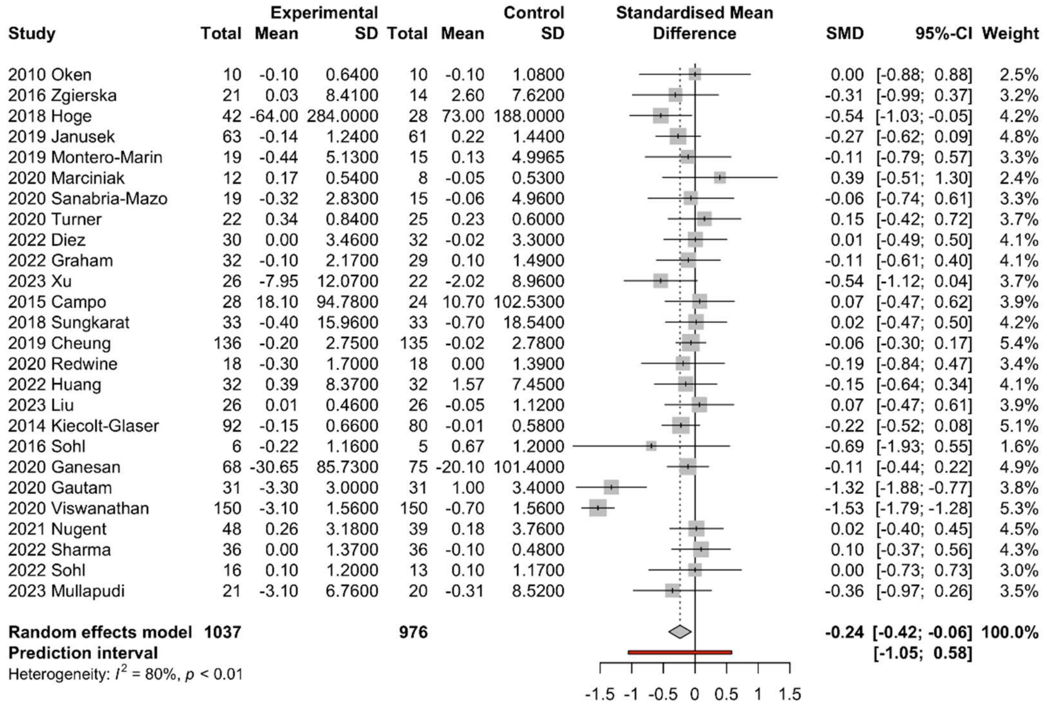

(E) IL-1

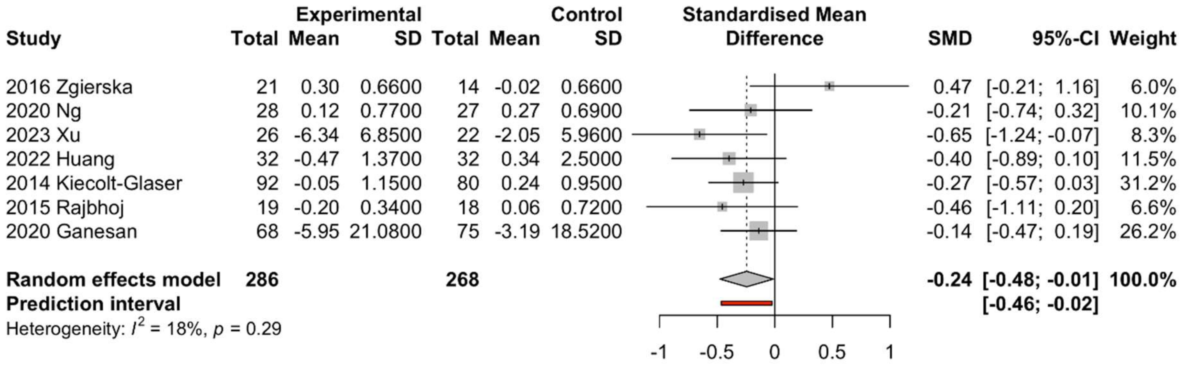

(F) IL-8

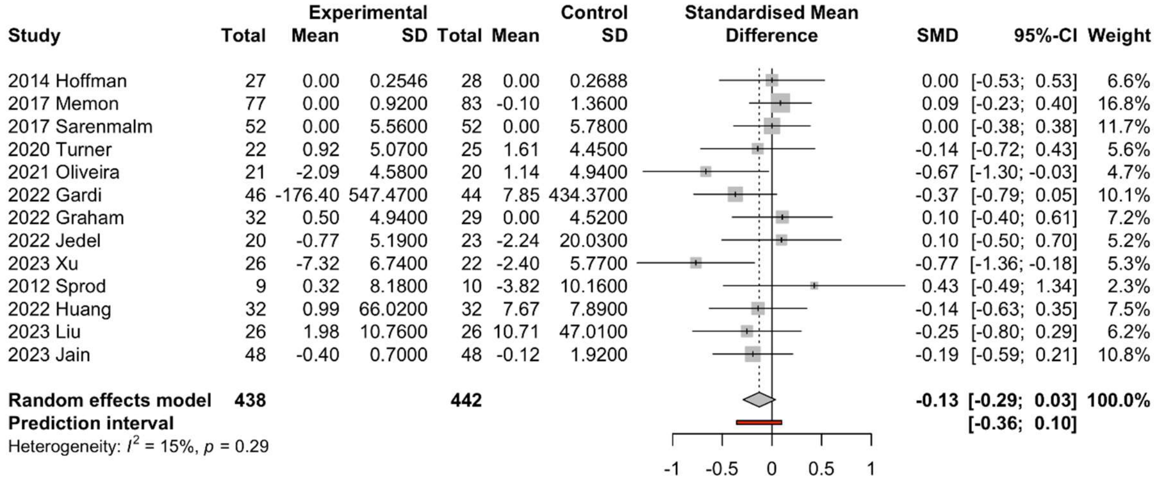

(G) IL-17

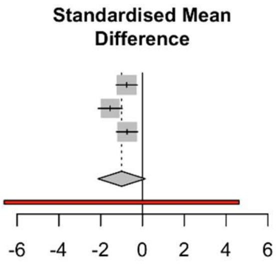

Study	Experimental Total	Experimental Mean	Experimental SD	Control Total	Control Mean	Control SD	SMD	95%-CI	Weight
2022 Huang	32	-0.09	6.1300	32	4.50	5.8600	-0.76	[-1.26; -0.25]	34.3%
2020 Gautam	31	-19.00	25.1000	31	18.70	23.0000	-1.55	[-2.12; -0.97]	31.4%
2023 Gautam	32	-16.50	36.2100	32	19.10	57.1700	-0.73	[-1.24; -0.23]	34.3%
**Random effects model**	**95**			**95**			**-1.00**	**[-2.13; 0.13]**	**100.0%**
**Prediction interval**								**[-6.62; 4.62]**	

Heterogeneity: $I^2 = 63\%$, $p = 0.07$

(H) IL-1ra

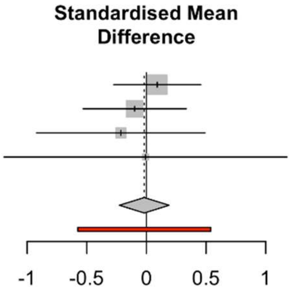

Study	Experimental Total	Experimental Mean	Experimental SD	Control Total	Control Mean	Control SD	SMD	95%-CI	Weight
2020 Järvelä-Reijonen	57	27.10	584.0600	58	-30.10	665.9000	0.09	[-0.28; 0.46]	48.2%
2012 Irwin	46	-16.00	236.0200	37	6.00	198.0400	-0.10	[-0.53; 0.33]	34.4%
2014 Bower	16	10.00	135.0600	15	70.50	369.4400	-0.21	[-0.92; 0.49]	12.9%
2016 Sohl	6	46.76	293.6500	5	49.92	430.7600	-0.01	[-1.19; 1.18]	4.6%
**Random effects model**	**125**			**115**			**-0.02**	**[-0.23; 0.19]**	**100.0%**
**Prediction interval**								**[-0.58; 0.54]**	

Heterogeneity: $I^2 = 0\%$, $p = 0.86$

(I) ESR

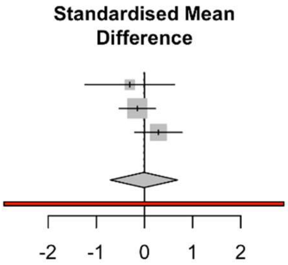

Study	Experimental Total	Experimental Mean	Experimental SD	Control Total	Control Mean	Control SD	SMD	95%-CI	Weight
2023 Dua	9	-3.80	6.6500	9	-0.90	10.8900	-0.31	[-1.24; 0.62]	12.3%
2023 Singh	57	-3.82	41.1200	52	1.38	26.4000	-0.15	[-0.52; 0.23]	51.9%
2021 Ewais	33	-2.00	12.7500	31	-5.00	6.6300	0.29	[-0.20; 0.78]	35.8%
**Random effects model**	**99**			**92**			**-0.01**	**[-0.71; 0.69]**	**100.0%**
**Prediction interval**								**[-2.92; 2.90]**	

Heterogeneity: $I^2 = 14\%$, $p = 0.31$

(J) Cortisol

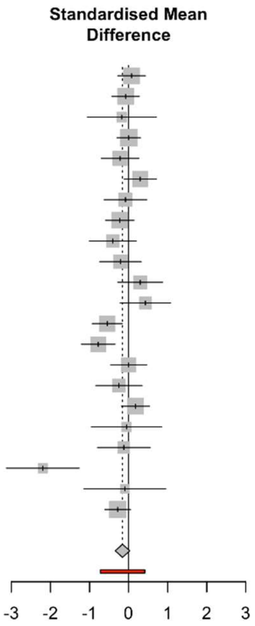

Study	Experimental Total	Experimental Mean	Experimental SD	Control Total	Control Mean	Control SD	SMD	95%-CI	Weight
2008 McCain_R	65	0.13	1.7900	57	-0.01	1.8300	0.08	[-0.28; 0.43]	6.1%
2008 McCain_S	68	-0.15	1.7900	57	-0.01	1.8300	-0.08	[-0.43; 0.28]	6.2%
2010 Oken	10	-1.30	6.1620	10	-0.50	0.9847	-0.17	[-1.05; 0.70]	2.3%
2013 Malarkey	84	0.01	24.3800	85	-0.03	0.2913	0.00	[-0.30; 0.30]	6.8%
2018 Hoge	42	-152.00	256.0000	28	-89.00	328.0000	-0.22	[-0.70; 0.26]	4.9%
2018 Nery	62	-0.04	0.3200	37	-0.12	0.1400	0.30	[-0.11; 0.71]	5.6%
2020 González-Moret	37	-0.41	1.5800	20	-0.24	2.7700	-0.08	[-0.63; 0.46]	4.3%
2020 Järvelä-Reijonen	57	-5.90	60.3600	58	7.20	54.7600	-0.23	[-0.59; 0.14]	6.0%
2020 Mirmahmoodi	22	-0.50	1.7700	22	0.23	1.7700	-0.41	[-1.00; 0.19]	3.9%
2020 Ng	28	0.01	0.4200	27	0.09	0.3900	-0.21	[-0.74; 0.32]	4.4%
2020 Turner	22	10.90	236.6000	25	-64.64	261.1500	0.30	[-0.28; 0.87]	4.0%
2021 Cohen	21	0.27	1.4600	17	-0.35	1.3400	0.43	[-0.22; 1.08]	3.5%
2022 Alhawatmeh	54	-210.34	289.4600	54	-40.25	325.8300	-0.55	[-0.93; -0.16]	5.8%
2022 Gardi	46	-1.18	1.0700	44	-0.22	1.3700	-0.78	[-1.21; -0.35]	5.4%
2022 Saban	39	-0.03	0.0600	34	-0.03	0.3000	0.00	[-0.46; 0.46]	5.0%
2022 Subramanian	22	-210.52	269.3700	22	-134.64	328.0000	-0.25	[-0.84; 0.35]	3.9%
2008 McCain_T	62	0.30	1.6800	57	-0.01	1.8300	0.18	[-0.18; 0.54]	6.1%
2012 Sprod	9	1.98	14.2900	10	2.65	10.3300	-0.05	[-0.95; 0.85]	2.2%
2013 Chan	18	-1.00	6.6800	16	-0.10	7.6600	-0.12	[-0.80; 0.55]	3.3%
2020 Lu	14	-19.07	5.6100	16	-6.42	5.6000	-2.20	[-3.13; -1.26]	2.1%
2009 Banasik	7	-0.19	1.0000	7	-0.11	0.4000	-0.10	[-1.15; 0.95]	1.8%
2020 Ganesan	68	-44.05	91.8300	75	-14.91	113.4700	-0.28	[-0.61; 0.05]	6.4%
**Random effects model**	**857**			**778**			**-0.16**	**[-0.34; 0.03]**	**100.0%**
**Prediction interval**								**[-0.73; 0.41]**	

Heterogeneity: $I^2 = 57\%$, $p < 0.01$

(K) sIgA

Study	Experimental Total	Experimental Mean	Experimental SD	Control Total	Control Mean	Control SD	Standardised Mean Difference	SMD	95%-CI	Weight
2013 Chan	18	29.10	96.5200	16	8.50	121.1200		0.18	[-0.49; 0.86]	37.4%
2010 Fan	17	137.62	173.4600	18	77.15	135.5200		0.38	[-0.29; 1.05]	38.0%
2022 Martínez-Borrás	13	6.26	8.1300	11	1.23	4.7400		0.71	[-0.12; 1.55]	24.6%
**Random effects model**	**48**			**45**				**0.39**	**[-0.23; 1.01]**	**100.0%**
**Prediction interval**									**[-2.29; 3.07]**	

Heterogeneity: $I^2 = 0\%$, $p = 0.63$

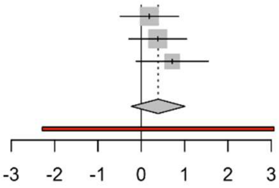

Supplementary Fig. S4 Funnel plot for publication bias of the standardized mean difference of mind-body interventions effect on biomarkers. (A) CRP, (B) IL-6, (C) TNF-α, (D) IL-1, (E) IL-8, (F) Cortisol.

(A) CRP

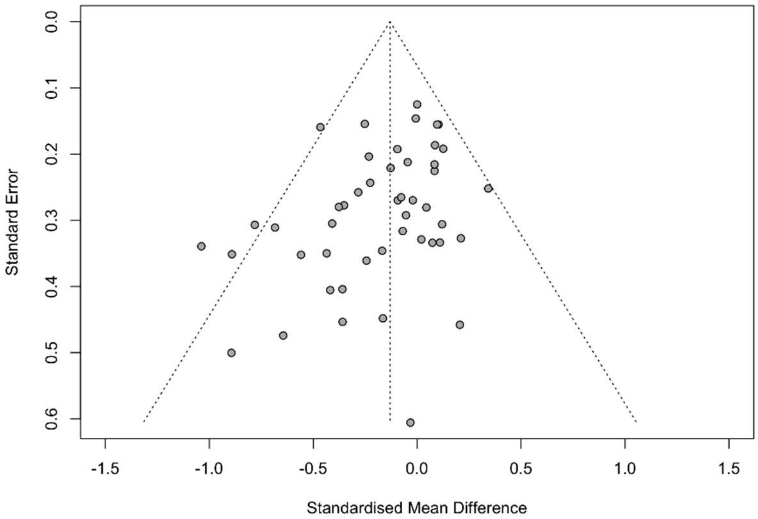

(B) IL-6

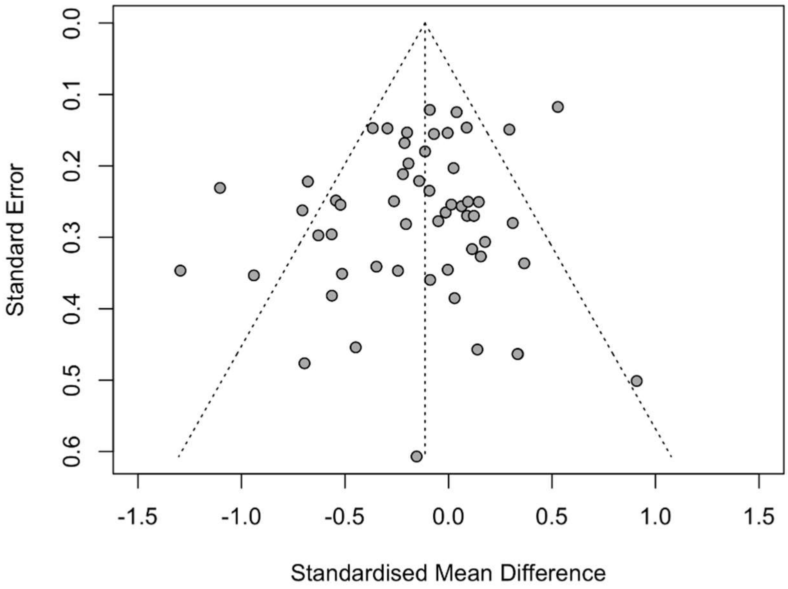

(C) TNF-α

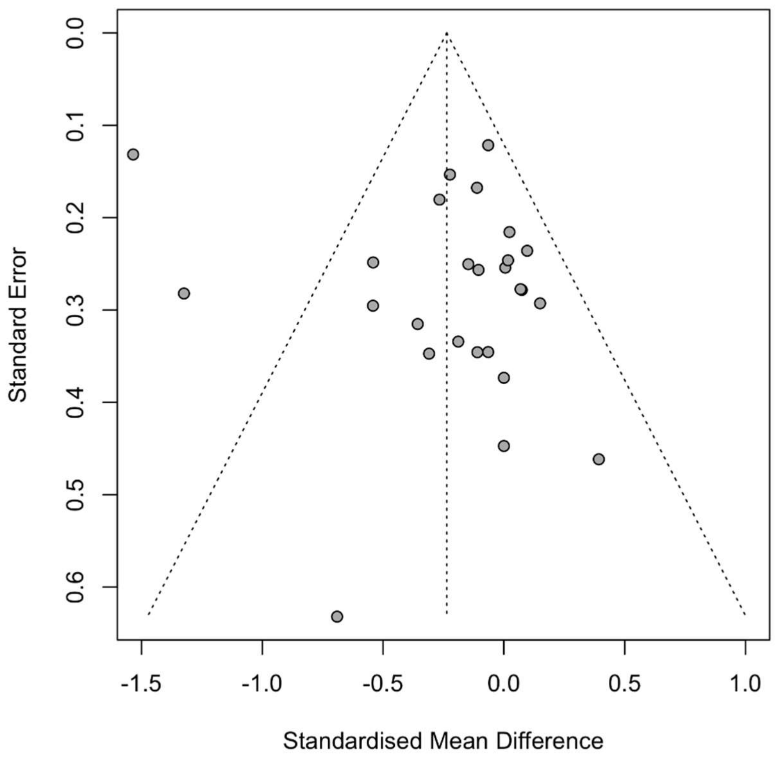
Standard Error
0.0
0.1
0.2
0.3
0.4
0.5
0.6
-1.5
-1.0
-0.5
0.0
0.5
1.0
Standardised Mean Difference

(D) IL-1

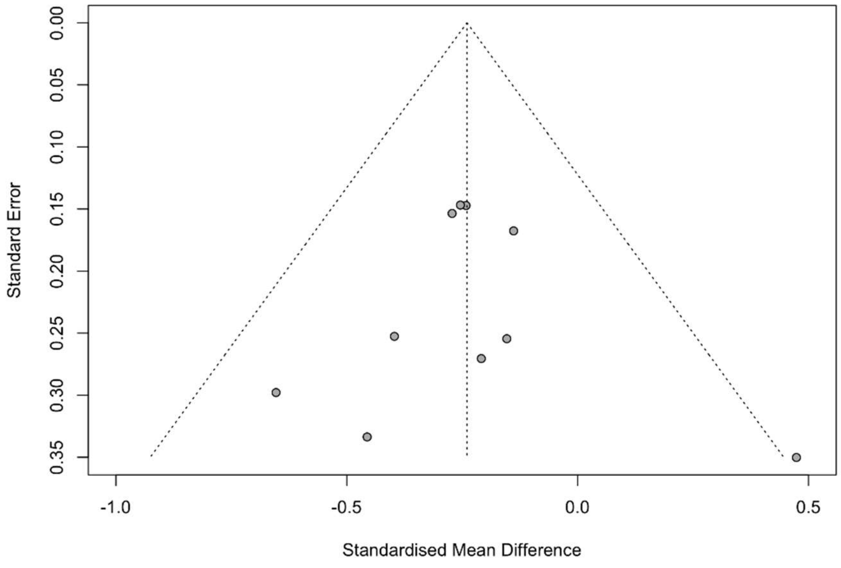
Standard Error
0.00
0.05
0.10
0.15
0.20
0.25
0.30
0.35
-1.0
-0.5
0.0
0.5
Standardised Mean Difference

(E) IL-8

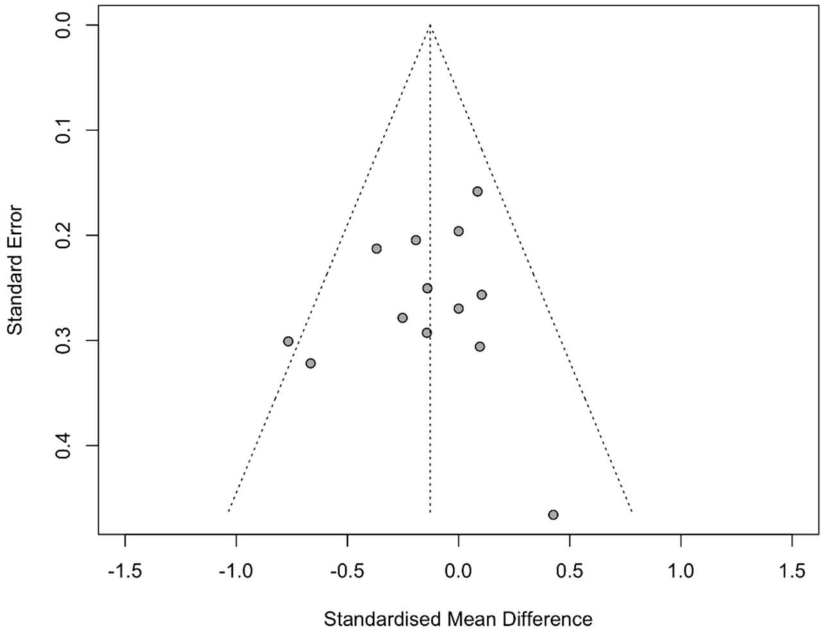

(F) Cortisol

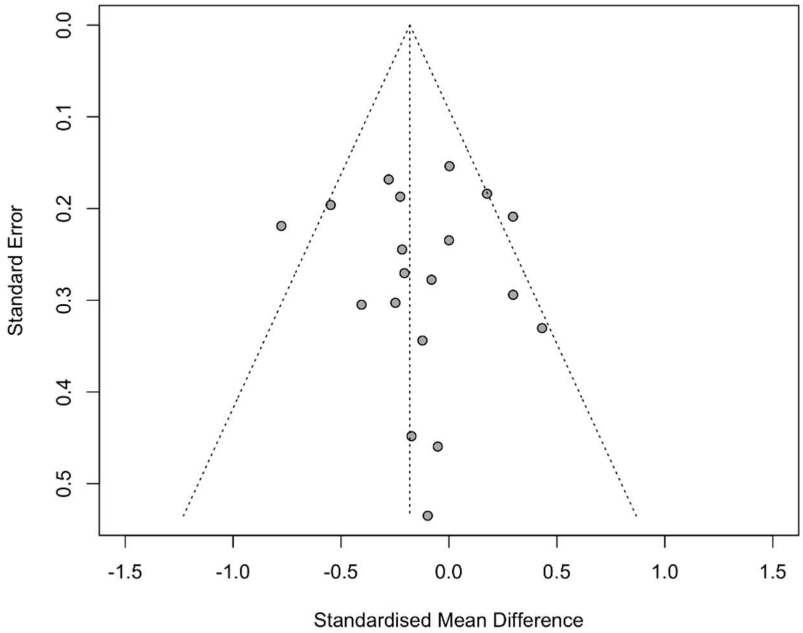

Supplementary Fig. S5 Funnel plot of the Trim-and-fill analysis of mind-body interventions effect on biomarkers. (A) CRP, (B) IL-6, (C) IL-6-CNS, (D) IL-6-cancer, (E) TNF-α, (F) IL-10, (G) IFN-γ, (H) BDNF, (I) Cortisol.

(A) CRP

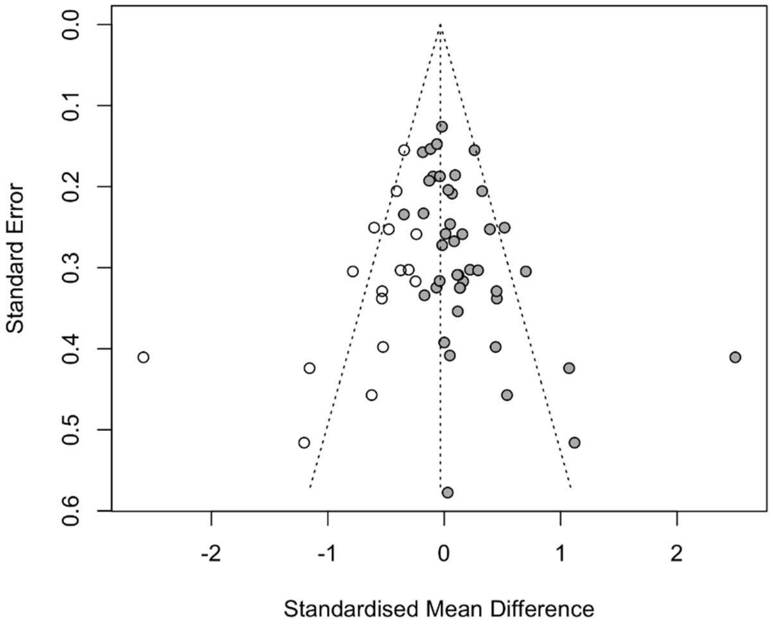

(B) IL-6

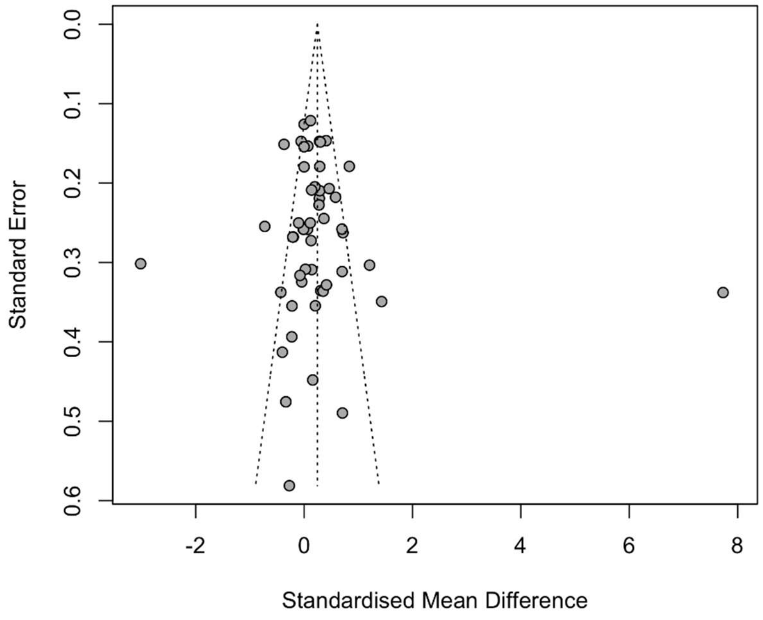

(C) IL-6-CNS

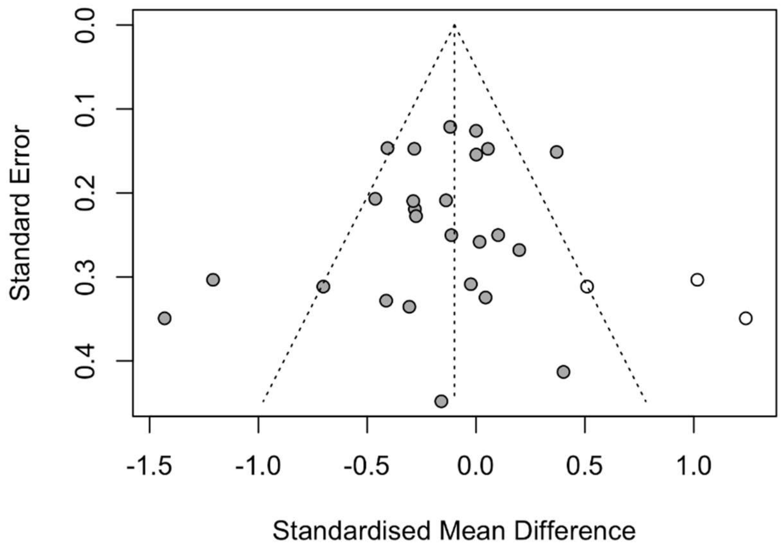

(D) IL-6-cancer

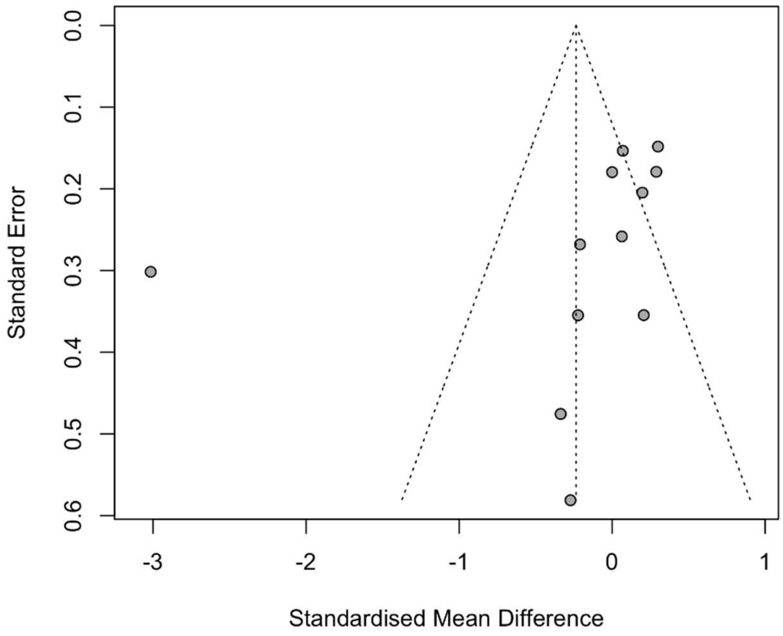

(E) TNF-α

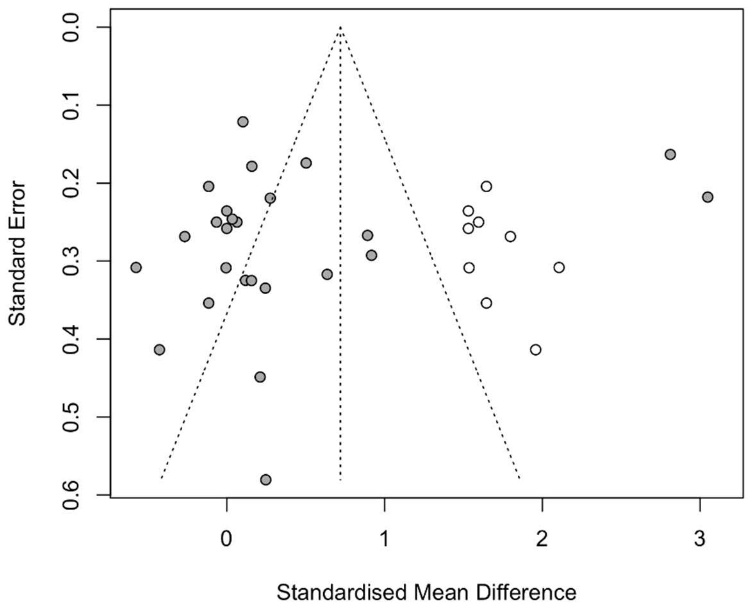

Standard Error
0.0
0.1
0.2
0.3
0.4
0.5
0.6
0
1
2
3
Standardised Mean Difference

(F) IL-10

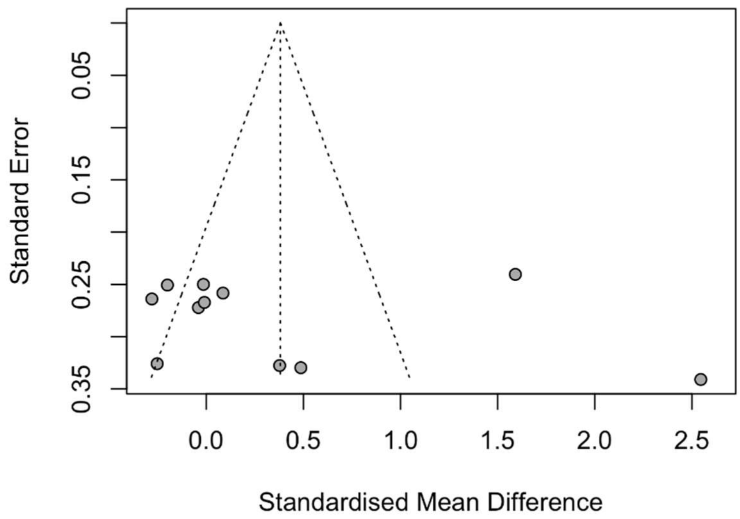

Standard Error
0.05
0.15
0.25
0.35
0.0
0.5
1.0
1.5
2.0
2.5
Standardised Mean Difference

(G) IFN-γ

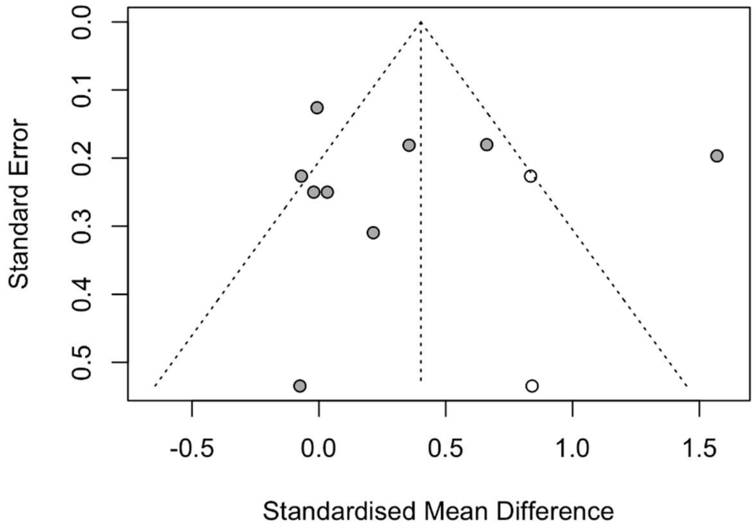
Standard Error
0.0
0.1
0.2
0.3
0.4
0.5
-0.5
0.0
0.5
1.0
1.5
Standardised Mean Difference

(H) BDNF

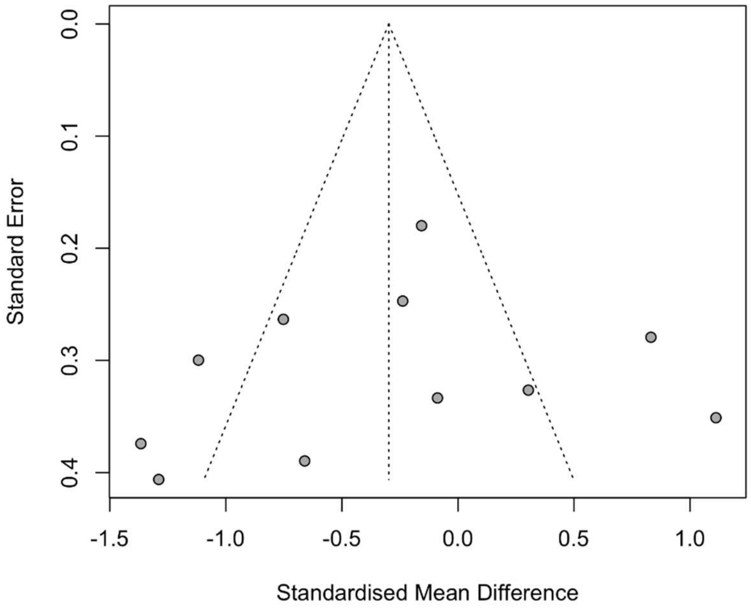
Standard Error
0.0
0.1
0.2
0.3
0.4
-1.5
-1.0
-0.5
0.0
0.5
1.0
Standardised Mean Difference

(I) Cortisol

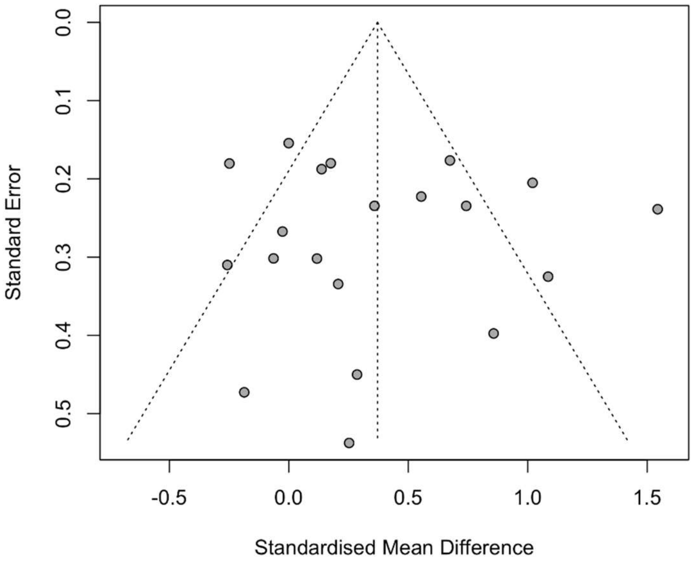

Standard Error
0.0
0.1
0.2
0.3
0.4
0.5
-0.5
0.0
0.5
1.0
1.5
Standardised Mean Difference

Supplementary Table. S1 Statistical information between groups for each biomarker

Study		Pre-MBI		Post-MBI			Pre-Ctrl		Post-Ctrl	
CRP	N1	MD1	SD1	MD2	SD2	N3	MD3	SD3	MD4	SD4
2010 Oken	10	4.3	4	2.4	2.6	10	3.1	4.1	2	1.9
2012 Creswell	20	2.98	5.14	2.09	5.59	20	3.42	5.23	3.06	5.23
2013 Malarkey	84	5.6	3.67	4.7	3.2	85	5.3	3.69	5.67	3.6
2014 Jedel	27	3.58	8.5	3.71	6.01	28	4.37	5.57	5.45	7.87
2015 Bower	30	1.24	1.7	1.22	1.66	22	1.45	1.81	1.32	1.75
2016 Zgierska	21	1.8	2.6	0.9	9.9	14	0.5	2.62	0.6	6.66
2017 Memon	81	2.3	2.4	2.9	3.9	85	1.9	1.9	2.1	2.4
2018 Smith	18	9.595	10.849	5.611	5.56	18	5.74	5.111	5.624	2.26
2019 Andrés-Rodríguez	30	2.92	3.19	2.44	2.89	27	2.44	2.72	2.27	2.35
2019 Meyer	126	1.6	5.1	1.7	5.1	130	1.5	4.61	1.6	5.19
2019 Montero-Marin	19	5.6	7.95	2.89	2.56	15	3.46	4.42	4.07	4.19
2019 Villalba	58	1.36	2.06	1.18	1.75	36	1.05	1.68	0.99	1.68
2019 Wynne	37	1.67	1.48	2.33	2.22	42	2.67	2.96	3	3.7
2019 Wirth	19	1.47	0.13	1.56	1.86	17	1.88	0.64	1.86	0.44
2020 González-Moret	37	1.93	2.47	2.43	3.05	20	2.46	3.81	5.25	7.73
2020 Järvelä-Reijonen	57	1.2	2.26	1.5	3.77	58	1.5	3.8	1.4	3.04
2020 Marciniak	12	1.57	1.65	1.49	1.55	8	1.72	1.66	1.15	1.62
2020 Mirmahmoodi	22	15.5	5.21	14.32	5.26	22	12.09	6.24	11.5	6.28
2020 Ng	28	0.21	0.3334	0.181	0.3387	27	0.217	0.3274	0.197	0.3377
2020 Sanabria-Mazo	19	4.68	6.42	3.85	5.5	15	3.54	4.36	4	4.12
2020 Turner	22	1.84	2.16	1.32	1.23	25	2.15	1.89	1.78	2.24
2021 Cohen	21	1.16	3.82	0.83	1.45	17	2.39	5.75	0.99	1.32
2021 Ewais	33	0.8	4.07	0.6	3.78	31	2	2.37	0.3	0.54
2022 Alhawatmeh	54	334.95	167.82	357.49	178.11	54	367.89	191.96	415.3	195.5
2022 Graham	32	3.42	5.49	1.74	2.39	29	4.1	5.34	4.67	7.83
2022 Henneghan	13	1.47	1.48	0.95	0.64	12	1	0.97	1.16	1.18
2022 Jedel	20	0.21	0.94	0.26	1.48	23	0.45	1.73	0.28	0.62
2022 Oswald	48	1.52	1.79	0.96	1.62	49	1.75	2.3	1.87	2.4
2022 Subramanian	22	29.82	11.51	8.45	2.91	22	25	9.56	11.36	3.79
2023 Bower	85	3.56	4.9	4.23	6.3	81	4	5.7	3.92	5
2023 Lindsay	92	1.94	3.45	2.12	2.11	95	2.54	3.41	2.75	3.61
2011 Lavretsky	33	2.9	2.5	1.8	1.6	35	2.6	2.3	2.2	2.1
2012 Irwin	46	1.66	1.12	1.58	1.24	37	1.56	1.69	1.75	1.88
2020 Redwine	18	3.7	4.8	4.5	4.4	18	2	3	2.2	2.4
2008 Pullen	9	2.18	0.34	1.75	0.39	10	2	0.23	1.9	0.4

2014 Bower	16	2.85	6.39	2.22	3.92	15	1.52	0.28	2.38	3.59
2016 Sohl	6	3.75	6.67	3.55	5.7	5	5.2	3.56	5.6	23.33
2020 Seguin	19	3.5	3.8	3	3.5	18	2.8	3.8	2.2	2.3
2020 Viswanathan	150	56.1	1.1	54.2	1.1	150	173.7	1.1	91.4	1.1
2021 Hiles	13	3	3.31	3	1.65	8	1	10.77	4	5.98
2021 Nugent	48	4.62	5.87	4.41	6.17	39	4.65	7.69	3.7	4.9
2022 Greaney	13	2.75	1.41	1.57	0.53	12	3	1.26	2.52	1.84
2023 Chauhan	23	5.41	4.84	2.57	2.88	23	3.59	3.5	5.17	4.23
2023 Singh	57	17.69	20.68	18.5	22.73	52	16.87	11.83	14.53	11.31

IL6	N1	MD1	SD1	MD2	SD2	N3	MD3	SD3	MD4	SD4
2010 Oken	10	2.2	1.2	2	1.2	10	1.5	1.3	2.8	4
2014 Jedel	27	2	0.06	1.99	0.09	28	1.98	0.08	1.96	0.08
2015 Bower	30	1.24	0.87	1.19	0.67	22	1.16	0.58	1.32	0.63
2016 Creswell	18	1.87	1.32	1.45	1.36	17	1.17	1.32	1.41	1.24
2016 Zgierska	21	3	3.5	2.2	7.01	14	1.8	1.3	2.1	6.17
2017 Sarenmalm	62	1	1.19	1	0.81	52	1.1	1.48	1.2	0.96
2018 Hoge	42	157	145	120	114	28	63	47	96	70
2018 Smith	18	2.933	1.655	2.452	0.92	18	2.155	1.261	2.448	0.675
2019 Andrés-Rodríguez	30	4.09	4.22	4.16	4.36	27	7.88	12.36	8.15	14.98
2019 Janusek	63	1.81	1.61	1.43	0.91	61	1.85	1.5	1.68	1.05
2019 Meyer	126	1.6	3.4	1.6	3.97	131	1.7	4.05	1.5	2.89
2019 Montero-Marin	19	2.9	1.56	2.39	0.7	15	3.18	1.19	3.13	1.6
2020 Marciniak	12	1.57	0.65	2	1.31	8	2.4	1.82	2.48	2.86
2020 Ng	28	0.344	0.5715	0.459	0.5715	27	0.392	0.5352	0.407	0.5924
2020 Sanabria-Mazo	19	3.35	2.63	3.44	1.12	15	3.04	1.18	3.14	1.87
2021 Cohen	21	0.47	0.48	0.46	0.32	17	0.83	1.19	0.66	0.65
2021 Ewais	33	1.02	0.48	2.23	2.28	31	1.59	1.31	2.46	1.85
2021 Oliveira	21	3.87	0.93	2.74	0.58	20	3.25	0.56	3.33	0.37
2022 Diez	30	18.54	29.14	18.82	28.38	32	11.24	29.59	10.96	27.99
2022 Gardi	46	3.06	3.41	2.12	3.04	44	3.16	1.97	3.05	1.7
2022 Graham	32	4.4	6.1	5.1	7.49	29	2.9	3.15	3.1	3.94
2022 Jedel	20	1.97	1.21	2.09	1.83	23	2.54	2.73	2.15	1.82
2022 Lindsay	89	78.18	37.36	92.84	41.42	93	84.41	36.84	82.84	39.83
2022 Ng	93	1.24	0.72	1.05	0.61	93	1.19	0.66	1.3	0.86
2022 Oswald	48	1.88	1.62	1.55	1.69	49	2.18	1.85	1.79	1.78
2022 Saban	39	9.17	1.01	8.89	1	34	8.99	1.14	8.84	0.74
2023 Bower	85	1.04	1.8	0.94	0.9	81	0.8	0.6	0.81	0.6
2023 Lindsay	92	2.71	2.59	2.86	2.88	95	3.69	2.63	3.5	2.83

2023 Xu	26	55.5	3.73	49.18	6.26	22	55.54	2.89	52.72	3.13
2006 Chen	44	138.66	78.63	100.64	47.35	43	114.72	37.81	140.56	56.27
2011 Janelsins	9	2.63	3.96	4.63	6.97	10	2.44	1.79	2.42	1.47
2012 Irwin	46	2.25	1.43	2.07	1.14	37	1.87	1.09	1.95	1.43
2012 Sprod	9	2.63	3.96	4.63	6.96	10	2.44	1.77	2.42	1.74
2015 Campo	28	6.8	14.89	9.1	3.48	24	6.8	5.41	5.3	3.56
2019 Cheung	136	0.25	1.06	0.12	1.12	135	0.12	1.06	0.13	1.12
2020 Redwine	18	1.5	0.8	2	1.4	18	1.3	0.7	1.3	0.7
2021 Qi	22	-0.31	0.31	-0.55	0.36	26	-0.59	0.4	-0.53	0.22
2022 Li	32	2.45	1.51	2.3	1.06	32	2.19	1.62	1.86	1.04
2022 Ng	95	1.29	0.66	1.04	0.56	93	1.19	0.66	1.3	0.86
2008 Pullen	9	17.5	5.9	13.6	4.5	10	18	2	18.1	2
2013 Malarkey	84	1.9	2.75	1.91	16.04	85	1.7	2.77	1.76	1.01
2014 Bower	16	1.03	0.54	1.19	0.83	15	1.37	0.69	1.64	1.22
2014 Kiecolt-Glaser	92	9.83	0.45	9.69	0.48	80	9.84	0.047	9.83	0.052
2016 Sohl	6	1.88	1.9	3.21	6.09	5	2.17	4.67	4.59	4.61
2020 Ganesan	68	114.91	66.98	60.42	62.82	75	112.69	62.72	79.35	84.25
2020 Gautam	31	3.5	1.3	2.4	1.7	31	3.8	1.1	3.7	1.5
2020 Viswanathan	150	22.6	1.2	13.3	1.2	150	22.5	1.2	12.3	1.2
2021 Nugent	48	2.82	2.04	1.95	1.67	39	2.22	2.1	3.79	3.91
2022 Chanta	13	15.42	11.66	17.92	9.58	14	10.83	9.17	12.92	7.91
2022 Sohl	16	5.6	4.13	4.8	3.38	13	4.6	2.98	6.9	4.47
2022 Sharma	34	2.4	0.28	2.3	0.26	31	2.3	0.27	2.3	0.25
2023 Gautam	32	3.4	1.2	2.4	1.6	32	3.7	1.1	3.7	1.4
2023 Jain	48	8.8	0.57	11.09	0.9	48	9.96	0.47	8.28	0.64

IL1	N1	MD1	SD1	MD2	SD2	N3	MD3	SD3	MD4	SD4
2016 Zgierska	21	0.6	1.5	0.9	9.9	14	0.1	0.1	0.08	0.72
2022 Diez	30	2.88	1.37	2.73	1.39	32	1.64	1.07	1.77	1.25
2022 Ng	93	0.13	0.23	0.07	0.15	93	0.1	0.27	0.12	0.27
2020 Ng	28	1.652	0.5344	1.767	0.5503	27	1.619	0.4677	1.889	0.5092
2023 Xu	26	128.44	3.08	122.1	6.12	22	127.7	4.12	125.65	4.3
2022 Li	32	2.78	0.85	2.31	1.07	32	1.42	2.13	1.76	1.31
2014 Kiecolt-Glaser	92	8.39	0.083	8.34	0.086	80	8.25	0.071	8.49	0.079
2015 Rajbhoj	19	0.75	0.3	0.55	0.16	18	0.85	0.46	0.91	0.55
2020 Ganesan	68	11.97	20.4	6.02	5.31	75	12.58	15.4	9.39	10.28

IL8	N1	MD1	SD1	MD2	SD2	N3	MD3	SD3	MD4	SD4
2014 Jedel	27	1.34	0.18	1.34	0.18	28	1.38	0.19	1.38	0.19

2017 Memon	77	1.3	0.6	1.3	0.7	83	1.6	1.1	1.5	0.8
2017 Sarenmalm	62	11.5	3.19	12	4.44	52	12	2.96	12	4.96
2020 Turner	22	8.43	3.29	9.35	3.86	25	7.24	2.54	8.85	3.67
2021 Oliveira	21	8.17	3.84	6.08	2.5	20	7.77	2.93	8.91	3.98
2022 Gardi	46	459.55	437.27	283.15	329.42	44	473.66	260.96	481.51	347.24
2022 Graham	32	3.8	3.05	4.3	3.88	29	3.2	3.68	3.2	2.63
2022 Jedel	20	5.03	4.74	4.26	2.1	23	8.96	17.17	6.72	10.31
2023 Xu	26	128.35	3.41	121.03	5.81	22	129.32	4.31	126.92	3.84
2012 Sprod	9	9.37	5.4	9.69	6.15	10	11.06	8.13	7.24	6.1
2022 Li	32	9.36	65.35	10.35	9.15	32	7.26	6.25	14.93	4.82
2023 Jain	48	17.93	0.46	17.53	0.53	48	16.6	1.31	16.48	1.41

TNF-α	N1	MD1	SD1	MD2	SD2	N3	MD3	SD3	MD4	SD4
2010 Oken	10	1.1	0.4	1	0.5	10	1.2	0.9	1.1	0.6
2016 Zgierska	21	5	2.3	5.03	8.65	14	6.4	6.2	9	5.67
2018 Hoge	42	480	247	417	204	28	317	186	390	235
2019 Janusek	63	1.64	0.96	1.5	0.78	61	1.63	0.91	1.85	1.11
2019 Montero-Marin	19	6.05	2.75	5.61	4.33	15	6.1	2.87	6.23	4.09
2020 Marciniak	12	1.12	0.32	1.29	0.44	8	1.07	0.38	1.02	0.37
2020 Sanabria-Mazo	19	5.92	1.65	5.6	2.3	15	5.99	2.74	5.93	4.13
2020 Turner	22	1.78	0.43	2.12	0.7	25	1.74	0.39	1.97	0.43
2022 Diez	30	9.68	2.4	9.68	2.49	32	8.16	2.31	8.14	2.36
2022 Graham	32	2	1.66	1.9	1.39	29	2	1.05	2.1	1.05
2023 Xu	26	120.39	8.6	112.44	8.47	22	122.87	6.34	120.85	6.33
2015 Campo	28	19.7	75.19	37.8	57.7	24	34.4	63.11	45.1	80.81
2018 Sungkarat	33	8.8	11.6	8.4	11	33	6.3	15.51	5.6	10.15
2019 Cheung	136	1.55	1.89	1.35	2	135	1.31	1.94	1.29	2
2020 Redwine	18	3.3	1.2	3	1.2	18	2.7	0.5	2.7	1.3
2022 Li	32	19.52	7.16	19.91	4.34	32	20	6.14	21.57	4.22
2014 Kiecolt-Glaser	92	8.45	0.048	8.3	0.05	80	8.44	0.043	8.43	0.048
2016 Sohl	6	1.49	0.85	1.27	0.79	5	1.02	0.79	1.69	0.9
2020 Ganesan	68	91.7	71.8	61.05	46.84	75	96.7	81.09	76.6	60.88
2020 Gautam	31	17	3.5	13.7	3.8	31	17.5	6.9	18.5	5.4
2020 Viswanathan	150	13.8	1.1	10.7	1.1	150	9.2	1.1	8.5	1.1
2021 Nugent	48	3.17	2.18	3.43	2.32	39	3.81	2.31	3.99	2.97
2022 Sharma	36	4	1.3	4	0.43	36	4.4	0.32	4.3	0.36
2022 Sohl	16	1.6	0.94	1.7	0.75	13	1.7	0.83	1.8	0.83
2023 Mullapudi	21	12.99	5.67	9.89	3.68	20	11.46	2.34	11.15	8.19

IL17	N1	MD1	SD1	MD2	SD2	N3	MD3	SD3	MD4	SD4
2022 Diez	30	18.89	8.34	19.34	9.5	32	11.56	6.37	12.22	7.43
2022 Li	32	13.06	4.7	12.97	3.93	32	8.96	4.97	13.46	3.1
2020 Gautam	31	195	31	176.6	30.7	31	188.4	38.6	207.2	42.2
2023 Gautam	32	191.4	25.8	174.9	25.4	32	187.5	38.9	206.6	41.9

IL10	N1	MD1	SD1	MD2	SD2	N3	MD3	SD3	MD4	SD4
2014 Jedel	27	1.93	0.26	1.92	0.23	28	1.92	0.18	1.91	0.2
2019 Andrés-Rodríguez	30	8.71	8.56	9.5	9.53	27	13.21	12.45	11.85	11.14
2019 Montero-Marin	19	5.15	0.41	5.41	0.86	15	5.14	0.46	5.34	0.59
2020 Sanabria-Mazo	19	5.59	1.44	5.31	0.53	15	5.13	0.43	5.02	1.41
2022 Gardi	46	0.5	0.44	1.4	0.66	44	0.58	0.39	0.67	0.48
2022 Graham	32	7.3	6.93	6.1	4.72	29	8.1	4.47	8.9	8.68
2015 Campo	28	5.9	11.56	5.8	8.89	24	4.5	3.19	5	3.56
2018 Sungkarat	29	1.2	1.41	0.8	1.41	27	1.38	1.92	0.8	1.13
2022 Li	32	9.9	2.73	9.86	2.66	32	9.19	6.05	8.7	5.34
2015 Rajbhoj	19	3.22	1.13	4.09	2.2	18	3.24	1.43	2.87	1.24
2023 Gautam	32	71.1	12.1	100.9	11	32	66.7	23.4	64.1	24

IL1ra	N1	MD1	SD1	MD2	SD2	N3	MD3	SD3	MD4	SD4
2020 Järvelä-Reijonen	57	356.5	368.2	383.6	453.4	58	417.1	532.07	387	400.48
2012 Irwin	46	254	187	238	144	37	234	143	240	137
2022 Li	32	424	275.81	242.51	87.64	32	190.65	489.92	275.38	80.06
2014 Bower	16	220.9	95	230.9	96	15	279.4	250	349.9	272
2016 Sohl	6	287.42	154.67	334.18	249.61	5	317.16	65.03	367.08	425.82

IFN-γ	N1	MD1	SD1	MD2	SD2	N3	MD3	SD3	MD4	SD4
2008 McCain_R	65	343.32	156.73	466.44	209.46	57	599.51	291.88	366.94	175.99
2008 McCain_S	68	208.31	95.99	450.82	194.94	57	599.51	291.88	366.94	175.99
2016 Zgierska	21	10.4	7.1	11.7	4.52	14	9.4	5.1	9.6	5.94
2019 Meyer	126	141	136.12	140	136.12	131	152	144.63	148	161.99
2022 Graham	32	5.3	6.1	5.5	5.82	29	5.6	6.05	6	4.73
2022 Saban	39	8.57	1.25	8.49	1.03	34	8.31	1.15	8.26	1.08
2008 McCain_T	62	181.34	80.63	212.81	94.72	57	599.51	291.88	366.94	175.99
2011 Janelsins	7	1.34	2.42	1.17	1.78	9	7.79	9.7	10.21	15.55
2022 Li	32	14.18	9.52	13.91	15.74	32	13.23	8.87	12.05	7.69

BDNF	N1	MD1	SD1	MD2	SD2	N3	MD3	SD3	MD4	SD4
2018 Nery	62	9150	1000	9300	900	37	8700	1000	8800	1200

2019 Montero-Marin	19	23.03	6.65	16.34	5.02	15	19.78	6.89	22.8	4.69
2020 Ng	28	7.238	0.8731	6.495	0.889	27	7.311	0.9041	6.455	0.9197
2020 Sanabria-Mazo	19	22.72	8.24	20.47	6.13	15	19.34	6.62	21.54	7.08
2023 Xu	26	184.9	11.72	199.36	13.68	22	176.49	4.03	182.5	4.59
2018 Sungkarat	33	162.3	394	314.4	801.22	33	213.5	458.56	385.6	798.68
2020 Lu	14	33.04	5.95	36.66	4.61	16	33.95	6.77	34.76	6.52
2021 Solianik	15	15.83	10	30.83	12.5	15	16	8	15.5	10
2014 Ikai	18	149.1	258.6	190.7	598.8	18	274.1	471	347.6	652.2
2020 Cekanauskaite	18	643	357	1250	500	15	800	350	750	450
2020 Gautam	31	13.3	3.9	17.2	6.1	31	13.5	4.3	13.8	4.7

sIgA	N1	MD1	SD1	MD2	SD2	N3	MD3	SD3	MD4	SD4
2010 Fan	17	262.16	88.24	311.39	114.48	18	242.69	78.83	245.52	79.15
2022 Martínez-Borrás	13	7.44	3.89	13.7	7.14	11	7.07	3.46	8.3	3.24
2013 Chan	18	43.8	63.14	72.9	73	16	58.9	82.2	67.4	88.95

Cortisol	N1	MD1	SD1	MD2	SD2	N3	MD3	SD3	MD4	SD4
2008 McCain_R	65	0.31	0.59	0.437	1.69	57	0.277	0.61	0.266	1.72
2008 McCain_S	68	0.365	0.62	0.216	1.68	57	0.277	0.61	0.266	1.72
2010 Oken	10	4	4.6	2.7	4.1	10	1.2	0.9	0.7	0.4
2013 Malarkey	84	0.1	0.09	0.11	24.38	85	0.15	0.28	0.12	0.09
2018 Hoge	42	913	252	761	290	28	1040	383	951	430
2018 Nery	62	0.44	0.25	0.4	0.2	37	0.75	0.13	0.63	0.06
2020 González-Moret	37	1.75	1.19	1.34	1.07	20	2.18	1.92	1.94	2.01
2020 Järvelä-Reijonen	57	115.6	45.9	109.7	39.2	58	108.9	35.8	116.1	41.4
2020 Mirmahmoodi	22	18.77	4.05	18.27	4.28	22	16.45	3.78	16.68	3.98
2020 Ng	28	-1.009	0.3281	-1.001	0.2646	27	-0.996	0.2598	-0.873	0.2858
2020 Turner	22	313.65	123.48	324.55	201.82	25	353.96	199.15	289.32	168.97
2021 Cohen	21	-0.22	0.88	0.05	1.16	17	0.27	1.1	-0.08	0.76
2022 Alhawatmeh	54	641.24	234.39	430.9	169.8	54	629.35	236.65	589.1	224
2022 Gardi	46	3.24	0.82	2.06	0.69	44	3.27	1.03	3.05	0.9
2022 Saban	39	0.1	0.04	0.07	0.04	34	0.14	0.29	0.11	0.09
2022 Subramanian	22	505.63	120.04	295.11	241.12	22	444.75	175.52	310.11	277.07
2008 McCain	62	0.349	0.61	0.647	1.57	57	0.277	0.61	0.266	1.72
2012 Sprod	9	21.52	8.73	23.5	11.31	10	26.04	6.58	28.69	7.97
2013 Chan	18	4.3	4.83	3.3	4.63	16	4.4	6.19	4.3	4.5
2020 Lu	14	126.31	23.39	107.24	19.63	16	116.07	19.26	109.65	19.58
2009 Banasik	7	2.52	0.11	2.33	0.99	7	2.56	0.27	2.45	0.29
2020 Ganesan	68	132.85	67.92	88.8	61.8	75	134.61	89.02	119.7	70.37

ESR										
ESR	N1	MD1	SD1	MD2	SD2	N3	MD3	SD3	MD4	SD4
2021 Ewais	33	6	7.41	4	10.37	31	9	5.93	4	2.96
2023 Singh	57	38.27	31.99	34.45	25.84	52	28.87	19.88	30.25	17.37
